# Novel Insights on Benzo[*b*]thiophene Analogues for MAO-B Inhibition and Neuroprotection: Design, Synthesis, Molecular Modelling Studies and Biological Activity

**DOI:** 10.3390/antiox15030346

**Published:** 2026-03-10

**Authors:** Francesca Arrighi, Emanuela Berrino, Paolo Guglielmi, Simone Carradori, Guya Diletta Marconi, Jacopo Pizzicannella, Simone Guarnieri, Tiziano Tuccinardi, Giulio Poli, Federico Pepi, Anna Troiani, Chiara Salvitti, Alessia Di Noi, Michele Coluccia, Giorgio Buttitta, Virginia Pontecorvi, Arianna Granese, Paola Chimenti, Daniela Secci, Anel Petzer, Jacobus Petrus Petzer, Francesca Diomede

**Affiliations:** 1 Department of Drug Chemistry and Technologies, Sapienza University of Rome, P.le A. Moro 5, 00185 Rome, Italy; francesca.arrighi@uniroma1.it (F.A.); e.berrino@unilink.it (E.B.); federico.pepi@uniroma1.it (F.P.); anna.troiani@uniroma1.it (A.T.); chiara.salvitti@uniroma1.it (C.S.); alessia.dinoi@uniroma1.it (A.D.N.); michele.coluccia@uniroma1.it (M.C.); giorgio.buttitta@uniroma1.it (G.B.); virginia.pontecorvi@uniroma1.it (V.P.); arianna.granese@uniroma1.it (A.G.); paola.chimenti@uniroma1.it (P.C.); daniela.secci@uniroma1.it (D.S.); 2 Department of Life Science, Health, and Health Professions, Link Campus University, Via del Casale di San Pio V, 44, 00165 Rome, Italy; 3 Department of Pharmacy, “G. d’Annunzio” University of Chieti-Pescara, Via dei Vestini 31, 66100 Chieti, Italy; 4 Department of Innovative Technologies in Medicine & Dentistry, “G. d’Annunzio” University of Chieti-Pescara, 66100 Chieti, Italy; guya.marconi@unich.it (G.D.M.); jacopo.pizzicannella@unich.it (J.P.); francesca.diomede@unich.it (F.D.); 5 Department of Neuroscience, Imaging and Clinical Sciences, “G. d’Annunzio” University of Chieti-Pescara, Via Luigi Polacchi 11, 66100 Chieti, Italy; simone.guarnieri@unich.it; 6 Department of Pharmacy, Università di Pisa, Via Bonanno 6, 56126 Pisa, Italy; tiziano.tuccinardi@unipi.it (T.T.); giulio.poli@unipi.it (G.P.); 7 Department of Medico-Surgical Sciences and Biotechnologies, Sapienza University of Rome, Corso della Repubblica 79, 04100 Latina, Italy; 8 Centre of Excellence for Pharmaceutical Sciences, North-West University, Potchefstroom 2520, South Africa; anel.petzer@nwu.ac.za (A.P.); jacques.petzer@nwu.ac.za (J.P.P.); 9 Pharmaceutical Chemistry, School of Pharmacy, North-West University, Private Bag X6001, Potchefstroom 2520, South Africa

**Keywords:** benzothiophene, Parkinson’s disease, neuroprotection, ROS, docking, monoamine oxidase

## Abstract

Neurodegenerative disorders (NDs), such as Alzheimer’s disease and Parkinson’s disease (PD), represent a significant challenge for ageing populations, with their prevalence increasing worldwide. Elevated human Monoamine Oxidase B (*h*MAO-B) activity has been related to neurodegenerative progression, where it contributes, among others, to oxidative stress and neuroinflammation. The identification and optimization of selective *h*MAO-B inhibitors is therefore pivotal in addressing the progression of NDs. In this work we introduced 2-aroylbenzothiophene analogues as promising agents to mitigate neurodegeneration. The synthesized compounds were screened against *h*MAO-A and *h*MAO-B, identifying compounds **4**, **11**, and **12** as the most promising. In vitro studies in *h*GF and SH-SY5Y cells revealed distinct toxicity profiles, with compound **4** being the least tolerated at 100 µM. ROS generation was investigated as a possible mechanism underlying this toxicity. Compounds **4** (12.5 µM), **11**, and **12** (100 µM) were further evaluated for neuroprotective effects against 6-hydroxydopamine (6-OHDA)-induced toxicity in SH-SY5Y cells, showing a modest neuroprotective effect after 72 h at a sub-toxic 6-OHDA concentration (250 µM), comparable to the clinically used *h*MAO-B inhibitor (*R*)-(−)-Deprenyl at 100 µM. Finally, molecular modelling studies revealed that compound **4** establishes key stabilizing interactions within *h*MAO-B, accounting for its high inhibitory potency and selectivity over *h*MAO-A.

## 1. Introduction

Monoamine oxidases (MAOs) are flavoenzymes located on the outer mitochondrial membrane that catalyze the oxidative deamination of a wide range of biogenic and dietary amines. In humans, two isoforms have been identified: *h*MAO-A and *h*MAO-B [1,2,3]. These isoenzymes, while structurally similar, sharing conserved flavin adenine dinucleotide (FAD) binding domains, differ significantly in their substrate specificity and tissue distribution [4,5,6]. *h*MAO-A preferentially degrades serotonin and norepinephrine, whereas *h*MAO-B primarily metabolizes phenylethylamine and contributes significantly to dopamine catabolism in the brain [7]. *h*MAO substrates play cooperative roles in modulating diverse behaviours and cognitive functions, including aggression, motivation, and motor control [8,9]. Beyond their primary actions, the metabolites and by-products generated from substrate degradation exert broad effects on neural function [10]. Importantly, the oxidative deamination of these substrates by *h*MAOs produces hydrogen peroxide and reactive primary amines implicated in the molecular pathology of numerous neurological disorders [11,12]. Dysregulation of *h*MAO activity has thus been increasingly linked to the onset and progression of both neuropsychiatric and neurodegenerative diseases [13,14]. Among these, Alzheimer’s disease (AD) and Parkinson’s disease (PD) are the most prevalent, collectively contributing to a significant burden of disability in ageing populations [15,16,17]. AD affects an estimated 35–40 million individuals globally, while PD impacts approximately 11.9 million people worldwide [18,19,20,21]. Elevated *h*MAO-B activity has been particularly associated with PD progression, where it contributes to oxidative stress and dopaminergic neuronal loss in the substantia nigra [22]. Emerging evidence also implicates *h*MAO-B in the activation of the NLRP3 inflammasome, highlighting its contribution to neuroinflammatory processes [23,24]. Notably, elevated astrocytic *h*MAO-B expression has been shown to drive PD-like pathology in animal models, while altered expression patterns of both *h*MAO-A and *h*MAO-B have been reported in parkinsonian syndromes, including multiple system atrophy (MSA) and progressive supranuclear palsy (PSP), suggesting a broader role for *h*MAO dysregulation in the pathophysiology of these disorders [25]. Given the growing evidence of *h*MAO involvement in neurodegeneration, the search for selective and effective *h*MAO inhibitors remains a key therapeutic strategy [26,27].

Recent advances in the development of *h*MAO inhibitors have led to the discovery of novel chemical entities with improved pharmacological profiles [28]. These compounds are broadly classified based on isoform selectivity (*h*MAO-A vs. *h*MAO-B) and their mode of inhibition (reversible vs. irreversible) [29,30,31,32,33]. Selective *h*MAO-A inhibitors (*h*MAO-AIs) have demonstrated efficacy in the treatment of depressive disorders, whereas *h*MAO-B inhibitors (*h*MAO-BIs) are clinically valuable in the management of PD, particularly in combination with L-DOPA or dopamine agonists, and are increasingly being explored for therapeutic benefit in Alzheimer’s disease [34,35,36,37].

**Figure 1:**
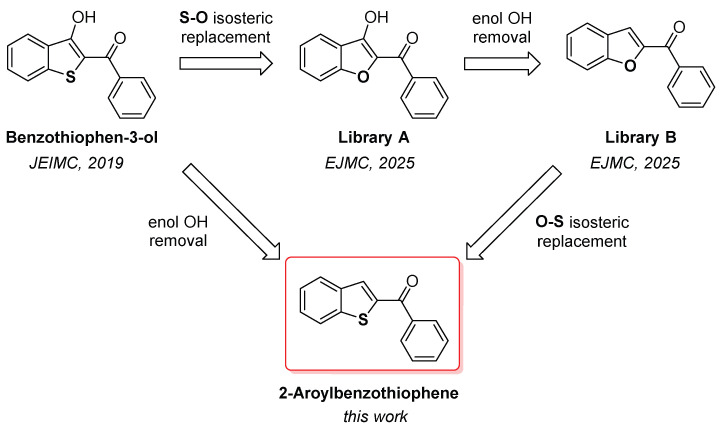
Design and development of the benzothiophene-based scaffold [38,39].

Continuing our efforts in the development of *h*MAO inhibitors, here we report the design, synthesis, and biological evaluation of novel benzo[*b*]thiophene analogues as potential *h*MAO inhibitors. The design of the novel *h*MAO inhibitors was based on two scaffolds, 2-aroylbenzofuran-3-ol and 2-aroylbenzofuran, previously investigated by our group (Figure 1) [38,39]. Interestingly, the isosteric replacement of the sulfur atom in 2-aroylbenzothiophen-3-ol with oxygen resulted in benzofuran analogues with limited activity (Figure 1, Library A) [39]. However, subsequent removal of the 3-hydroxyl group yielded 2-aroyl benzofuran derivatives with potent activity and high selectivity toward *h*MAO-B (Figure 1, Library B) [39]. Based on these results, we developed the 2-aroylbenzothiophene scaffold, which can be conceptually derived either (i) from the removal of the 3-hydroxyl group from the benzothiophene-3-ol core, or (ii) as an oxygen-to-sulfur isosteric replacement of the 2-aroylbenzofuran framework (Figure 1).

The designed 2-aroylbenzothiophenes were evaluated against both *h*MAO isoforms, and the most promising compounds were subsequently investigated at the cellular scale to evaluate their effects on two distinct cell lines (SH-SY5Y and *h*GF). Due to the prominent role that reactive oxygen species (ROS) play in neurodegenerative diseases [40,41,42,43], 2-aroylbenzothiophenes were also evaluated in the SH-SY5Y cell line for their effect on ROS levels. Evaluation of the compound’s neuroprotective activity in a cellular Parkinson’s disease model was also conducted (SH-SY5Y cells treated with 6-hydroxydopamine, 6-OHDA). Finally, in order to predict the binding mode of these compounds, molecular modelling studies were performed.

## 2. Materials and Methods

### 2.1. General

Unless otherwise stated, all reactions were conducted under a positive nitrogen atmosphere (balloon pressure) using washed, oven-dried glassware. Solvents and reagents were used as received without further purification. ^1^H and ^13^C NMR spectra were recorded at 400 and 101 MHz, respectively, on a Bruker spectrometer at room temperature, using CDCl_3_ and DMSO-*d*_6_ as solvents. The final concentration of the analyzed samples was approximately 30 mg/mL. Chemical shifts are reported in δ (ppm). ^1^H spectra are reported as follows: *δ*_H_ (spectrometer frequency, solvent): chemical shift/ppm (multiplicity, *J*-coupling constant(s), number of protons, assignment). ^13^C spectra are reported as follows: *δ*_C_ (spectrometer frequency, solvent): chemical shift/ppm (*J*-coupling constant C-F, assignment). The following abbreviations have been used to indicate multiplicity: br—broad; s—singlet; d—doublet; t—triplet; q—quartet; m—multiplet. Coupling constants *J* are given in Hertz (Hz). MestReNova software (version 14) was used for the processing and analysis of the NMR spectra. Accurate mass measurements were accomplished by means of an Orbitrap Exploris 120 mass spectrometer (Thermo Fisher Scientific, Waltham, MA, USA) operating at a resolving power of 120,000 at *m*/*z* 200. The instrument was calibrated daily using FlexMix Calibration Solution (Thermo Fisher Scientific), and the radical cation of fluoranthene at *m*/*z* 202.07770 was used as a lock mass through a scan-*to*-scan recalibration procedure. Mass spectra were acquired in positive ion mode over a 150–500 *m*/*z* range with an injection time of 100 ms, and the raw files thus obtained were processed using FreeStyle software v. 1.8 (Xcalibur package). Delta values < 1 ppm between theoretical and experimental masses were obtained for all the compounds analyzed. Depending on the ionization properties of these compounds, two different analytical strategies were employed, one of which was adapted from a procedure reported elsewhere [44]. Further details are provided in the Supplementary Information. Column chromatography was performed by employing silica gel (Sigma-Aldrich^®^, St. Louis, MO, USA, high purity grade, pore size 60 Å, 230–400 mesh particle size). The progress of all reactions and purifications was checked by thin-layer chromatography (TLC) performed on 0.2 mm silica gel–aluminum-backed plates, and visualization was performed under ultraviolet irradiation (254 nm). ChemBioDraw Ultra 12.0 was used to generate the systematic names of the reported compounds in accordance with IUPAC conventions. Recombinant *h*MAO-A and *h*MAO-B (5 mg protein/mL) and kynuramine dihydrobromide were obtained from Sigma-Aldrich.

### 2.2. Chemistry

Detailed synthetic methodologies are fully described in the Supporting Information.

#### ^1^H and ^13^C Characterization Data for the Title Compounds **1**–**15**

In the following ^1^H and ^13^C NMR data, the signals corresponding to benzothiophene are denoted as BT. The reported yield values refer to the overall yields across the three synthetic steps.

***Benzo[b]thiophen-2-yl(phenyl)methanone (1).*** White powder, yield 47%. ^1^H NMR (400 MHz, CDCl_3_) δ 7.32–7.37 (m, 1H, BT), 7.39–7.43 (m, 1H, BT), 7.44–7.46 (m, 2H, 1H BT + 1H Ar), 7.54–7.58 (m, 1H, BT), 7.79–7.86 (m, 5H, Ar). ^13^C NMR (101 MHz, CDCl_3_): δ 122.9 (C_BT_), 125.0 (C_BT_), 125.1 (C_BT_), 126.0 (C_BT_), 127.5 (C_BT_), 128.55 (2 × Ar), 129.3 (2 × Ar), 132.2 (Ar), 137.8 (Ar), 139.0 (C_BT_), 142.7 (C_BT_), 143.1 (C_BT_), 189.7 (C=O). Measured monoisotopic mass [C_15_H_10_O^32^S+H]^+^: *m*/*z* 239.0526 (Δ: 0.30 ppm)—Theoretical monoisotopic mass: *m*/*z* 239.05251.

***Benzo[b]thiophen-2-yl(p-tolyl)methanone (2).*** White powder, yield 20%. ^1^H NMR (400 MHz, CDCl_3_): δ 2.50 (s, 3H, CH_3_), 7.35–7.37 (d, J = 7.8 Hz, 2H, Ar), 7.42–7.46 (m, 1H, BT), 7.51–7.54 (m, 1H, BT), 7.86–7.89 (m, 3H, 1H BT + 2H Ar) 7.91–7.95 (m, 2H, BT). ^13^C NMR (101 MHz, CDCl_3_): δ 21.70 (CH_3_), 122.9 (C_BT_), 125.0 (C_BT_), 125.9 (C_BT_), 127.3 (C_BT_), 129.2 (2 × Ar), 129.5 (2 × Ar), 131.8 (Ar), 135.1 (C_BT_), 139.1 (C_BT_),142.6 (Ar), 143.3 (C_BT_), 143.3 (C_BT_), 189.3 (C=O). Measured monoisotopic mass [C_16_H_12_O^32^S+H]^+^: *m*/*z* 253.0682 (Δ: 0.04 ppm)—Theoretical monoisotopic mass: *m*/*z* 253.06816.

***Benzo[b]thiophen-2-yl(3-methoxyphenyl)methanone (3).*** White powder, yield 54%. ^1^H NMR (400 MHz, CDCl_3_): δ 3.82 (s, 3H, -OCH_3_), 7.09–7.12 (m, 1H, Ar), 7.35–7.45 (m, 5H, 2H BT + 3H Ar) 7.81–7.86 (m, 3H, 2H BT + 1H Ar). ^13^C NMR (101 MHz, CDCl_3_): δ 55.5 (OCH_3_), 113.8 (Ar), 118.8 (Ar), 121.8 (C_BT_), 122.9 (Ar), 125.0 (C_BT_), 126.1 (C_BT_), 127.4 (C_BT_), 129.5 (Ar), 132.2 (C_BT_), 139.0 (Ar), 139.1 (C_BT_), 142.7 (C_BT_), 143.2 (C_BT_), 159.7 (Ar), 189.4 (C=O). Measured monoisotopic mass [C_16_H_12_O_2_^32^S+H]^+^: *m*/*z* 269.0632 (Δ: 0.34 ppm)—Theoretical monoisotopic mass: *m*/*z* 269.06308.

***Benzo[b]thiophen-2-yl(4-methoxyphenyl)methanone (4).*** White powder, yield: 57%. ^1^H NMR (400 MHz, CDCl_3_): δ 3.84 (s, 3H, CH_3_) 6.93–6.96 (m, 2H, Ar), 7.32–7.36 (m, 1H, BT), 7.38–7.41 (m, 1H, BT), 7.78–7.91 (m, 5H, 3H BT + 2H Ar). 13C NMR (101 MHz, CDCl_3_): δ 55.5 (-OCH3), 113.8 (2 × Ar), 122.8 (CBT), 124.9 (CBT), 125.8 (CBT), 127.1 (CBT), 130.4 (Ar), 131.2 (CBT), 131.7 (2 × Ar), 139.0 (CBT), 142.4 (CBT), 143.7 (CBT), 163.3 (Ar), 188.2 (C=O). Measured monoisotopic mass [C_16_H_12_O_2_^32^S+H]^+^: *m*/*z* 269.0631 (Δ: 0.06 ppm)—Theoretical monoisotopic mass: *m*/*z* 269.06308.

***Benzo[b]thiophen-2-yl(3-fluorophenyl)methanone (5).*** White powder, yield 37%. ^1^H NMR (400 MHz, CDCl_3_): δ 7.34–7.38 (m, 1H, BT), 7.44–7.58 (m,3H, 1H BT+ 2H Ar), 7.62–7.65 (m, 1H, BT), 7.73–7.75 (m, 1H, BT), 7.90–7.96 (m, 3H, 1H BT + 2H Ar). ^13^C NMR (101 MHz, CDCl_3_): δ 116.0 (Ar), 119.4 (Ar), 119.6 (C_BT_), 122.9 (C_BT_), 125.02 (C_BT_), 125.05 (C_BT_), 125.2 (Ar), 130.3 (Ar), 132.4 (C_BT_), 138.9 (Ar), 139.8 (C_BT_), 142.5 (C_BT_), 142.8 (C_BT_), 161.33 (Ar), 188.2 (C=O). Measured monoisotopic mass [C_15_H_9_FO^32^S+H]^+^: *m*/*z* 257.0432 (Δ: 0.24 ppm)—Theoretical monoisotopic mass: *m*/*z* 257.04309.

***Benzo[b]thiophen-2-yl(4-fluorophenyl)methanone (6).*** White powder, yield 60%. ^1^H NMR (400 MHz, CDCl_3_): δ 7.23–7.28 (m, 2H, BT), 7.44- 7.46 (m, 1H, BT), 7.50–7.55 (m, 1H, BT), 7.87 (s, 1H, BT), 7.91–8.01 (m, 4H, Ar). ^13^C NMR (101 MHz, CDCl_3_): δ 115.7 (Ar), 122.9 (C_BT_), 125.1 (C_BT_), 126.0 (C_BT_), 127.5 (C_BT_), 131.9 (Ar), 134.0 (C_BT_), 139.0 (C_BT_), 142.6 (C_BT_), 142.8 (C_BT_), 188.1 (C=O). Measured monoisotopic mass [C_15_H_9_FO^32^S+H]^+^: *m*/*z* 257.0431 (Δ: 0.12 ppm)—Theoretical monoisotopic mass: *m*/*z* 257.04309.

***Benzo[b]thiophen-2-yl(3-chlorophenyl)methanone (7).*** White powder, yield 41%. ^1^H NMR (400 MHz, CDCl_3_): δ 7.46–7.54 (m, 3H, 2H BT + 1H Ar), 7.62–7.64 (m, 1H, Ar), 7.82–7.83 (d, J = 7.6, 1.4 Hz, 1H, BT), 7.88 (s, 1H, BT), 7.91–7.96 (m, 3H, 1H BT + 2H Ar). ^13^C NMR (101 MHz, CDCl_3_): δ 122.9 (C_BT_), 125.2 (C_BT_), 126. 2 (C_BT_), 127.3 (C_BT_), 127.7 (Ar), 129.2 (C_BT_), 129.9 (Ar), 132.4 (Ar) 132.5 (Ar), 134.7 (Ar), 138.9 (Ar), 139.4 (C_BT_), 142.5 (C_BT_), 142.8 (C_BT_), 188.2 (C=O). Measured monoisotopic mass [C_15_H_9_^35^ClO^32^S+H]^+^: *m*/*z* 273.0135 (Δ: −0.07 ppm)—Theoretical monoisotopic mass: *m*/*z* 273.01354.

***Benzo[b]thiophen-2-yl(4-chlorophenyl)methanone (8).*** White powder, yield 59%. ^1^H NMR (400 MHz, CDCl_3_): δ 7.44–7.47 (m, 1H, BT), 7.51–7.56 (m, 3H, 2H Ar + 1H BT), 7.87 (s, 1H, BT), 7.89–7.96 (m, 4H, 2H Ar + 2H BT). ^13^C NMR (101 MHz, CDCl_3_): δ 122.9 (C_BT_), 125.1 (C_BT_), 126.1 (C_BT_), 127.6 (C_BT_), 128.9 (2 × Ar), 130.7 (2 × Ar), 132.1 (C_BT_), 136.1 (Ar), 138.9 (Ar), 142.6 (C_BT_), 142.7 (C_BT_), 188.4 (C=O). Measured monoisotopic mass [C_15_H_9_^35^ClO^32^S+H]^+^: *m*/*z* 273.0136 (Δ: 0.26 ppm)—Theoretical monoisotopic mass: *m*/*z* 273.01354.

***Benzo[b]thiophen-2-yl(3-bromophenyl)methanone (9).*** White powder, yield 45%. ^1^H NMR (400 MHz, CDCl_3_): δ 7.43–7.47 (m, 2H, 1H BT + 1H Ar), 7.51–7.54 (m, 1H, BT), 7.77–7.79 (m, 1H, Ar), 7.85–7.87 (m, 2H, 1H BT + 1H Ar), 7.92–7.95 (m, 2H, Ar), 8.06–8.07 (m, 1H, BT). ^13^C NMR (101 MHz, CDCl_3_): δ 122.7 (C_BT_), 122.9 (C_BT_), 125.2 (C_BT_), 126.2 (C_BT_), 127.7 (C_BT_ + Ar), 130.1 (Ar), 132.5 (Ar), 135.8 (Ar), 138.9 (Ar), 139.6 (C_BT_), 142.4 (C_BT_), 142.8 (C_BT_), 188.1 (C=O). Measured monoisotopic mass [C_15_H_9_^79^BrO^32^S+H]^+^: *m*/*z* 316.9631 (Δ: 0.15 ppm)—Theoretical monoisotopic mass: *m*/*z* 316.96303.

***Benzo[b]thiophen-2-yl(4-bromophenyl)methanone (10).*** White powder, yield 56%. 1H NMR (400 MHz, CDCl_3_): δ 7.44–7.48 (m, 1H, BT), 7.50–7.54 (m, 1H, BT), 7.70–7.72 (m, 2H, Ar), 7.81–7.84 (m, 2H, Ar), 7.86 (s, 1 H, BT), 7.90–7.95 (m, 2H, BT). 13C NMR (101 MHz, CDCl_3_) δ 122.9 (CBT), 125.2 (CBT), 126.1 (CBT), 127.5 (CBT), 127.5 (Ar), 127.6 (CBT), 130.8 (2 × Ar), 131.8 (2 × Ar), 132.2 (Ar), 136.5 (CBT), 138.9 (CBT), 142.6 (CBT), 188.5 (C=O). Measured monoisotopic mass [C_15_H_9_^79^BrO^32^S+H]^+^: *m*/*z* 316.9631 (Δ: 0.27 ppm)—Theoretical monoisotopic mass: *m*/*z* 316.96303.

***Benzo[b]thiophen-2-yl(3-nitrophenyl)methanone (11).*** Yellow powder, yield 51%. ^1^H NMR (400 MHz, CDCl_3_): δ 7.36–7.40 (m, 1H, BT), 7.44–7.46 (m, 1H, BT), 7.67–7.71 (m, 1H, Ar), 7.80 (s, 1H, BT), 7.86–7.88 (m, 2H, BT), 8.16–8.19 (m, 1H, Ar) 8.40–8.43 (m, 1H, Ar), 8.68–8.69 (m, 1H, Ar). ^13^C NMR (101 MHz, CDCl_3_): δ 123.0 (Ar), 124.0 (C_BT_), 125.4 (C_BT_), 126.4 (C_BT_), 126.8 (C_BT_), 128.1 (C_BT_), 129.9 (Ar), 132.8 (Ar), 134.8 (Ar), 138.9 (Ar), 139.0 (C_BT_), 141.8 (C_BT_), 143.0 (C_BT_), 148.1 (Ar), 188.1 (C=O). Measured monoisotopic mass [C_15_H_9_NO_3_^32^S+H]^+^: *m*/*z* 284.0376 (Δ: 0.13 ppm)—Theoretical monoisotopic mass: *m*/*z* 284.03759.

***Benzo[b]thiophen-2-yl(4-nitrophenyl)methanone (12).*** Yellow powder, yield 49%. ^1^H NMR (400 MHz, CDCl_3_): δ 7.36–7.40 (m, 1H, BT), 7.44–7.48 (m, 1H, BT), 7.76 (s, 1H, BT), 7.82–7.88 (m, 2H, BT), 7.97–8.00 (m, 2H, Ar), 8.31–8.34 (m, 2H, Ar). ^13^C NMR (101 MHz, CDCl_3_): δ 123.0 (C_BT_), 123.8 (2 × Ar), 125.4 (C_BT_), 126.3 (C_BT_), 128.2 (C_BT_), 130.0 (2 × Ar), 133.1 (C_BT_), 138.8 (C_BT_), 142.0 (C_BT_), 143.0 (Ar), 143.1 (C_BT_), 149.9 (Ar), 187.8 (C=O). Measured monoisotopic mass [C_15_H_9_NO_3_^32^S]^+^: *m*/*z* 283.0297 (Δ: −0.15 ppm)—Theoretical monoisotopic mass: *m*/*z* 283.02977.

***4-(Benzo[b]thiophene-2-carbonyl)benzonitrile (13).*** White powder, yield 31%. ^1^H NMR (400 MHz, CDCl_3_): δ 7.45–7.49 (m, 1H, BT), 7.53–7.57 (m, 1H, BT), 7.85–7.87 (m, 2H, Ar), 7.88 (s, 1H, BT), 7.91–7.97 (m, 2H, Ar), 8.01–8.03 (m, 2H, Ar). ^13^C NMR (101 MHz, CDCl_3_): δ 115.8 (Ar), 117.9 (CN), 123.0 (C_BT_), 125.4 (C_BT_), 126.3 (C_BT_), 128.1 (C_BT_), 129.6 (2 × Ar), 132.2 (2 × Ar), 132.9 (C_BT_), 138.8 (C_BT_), 141.4 (C_BT_), 142.0 (Ar), 143.0 (C_BT_), 189.2 (C=O). Measured monoisotopic mass [C_16_H_9_NO^32^S]^+^: *m*/*z* 263.0400 (Δ: 0.28 ppm)—Theoretical monoisotopic mass: *m*/*z* 263.03994.

***[1,1′-Biphenyl]-4-yl(benzo[b]thiophen-2-yl)methanone (14).*** Yellow powder, yield 39%. ^1^H NMR (400 MHz, CDCl_3_): δ 7.43–7.48 (m, 2H, 1H BT + 1H Ar), 7.51–7.55 (m, 3H, 1H BT + 2H Ar), 7.69–7.71 (m, 2H, Ar), 7.78–7.80 (m, 2H, Ar), 7.92–7.97 (m, 3H, 1H BT + 2H Ar), 8.04–8.06 (m, 2H, Ar). ^13^C NMR (101 MHz, CDCl_3_): δ 122.9 (C_BT_), 125.0 (C_BT_), 126.0 (C_BT_), 127.2 (2 × Ar), 127.3 (2 × Ar), 127.4 (Ar), 128.2 (Ar), 129.0 (2 × Ar), 129.9 (2 × Ar), 132.0 (Ar), 136.5 (C_BT_), 139.1 (C_BT_), 139.9 (C_BT_), 142.7 (Ar), 143.2 (C_BT_), 145.3 (C_BT_), 189.2 (C=O). Measured monoisotopic mass [C_21_H_14_O^32^S+H]^+^: *m*/*z* 315.0839 (Δ: 0.14 ppm)—Theoretical monoisotopic mass: *m*/*z* 315.08381.

***Benzo[b]thiophen-2-yl(naphthalen-2-yl)methanone (15).*** Yellow powder, yield 55%. ^1^H NMR (400 MHz, CDCl_3_): δ 7.34–7.38 (m, 1H, BT), 7.41–7.45 (m, 1H, BT), 7.52–7.59 (m, 2H, Ar), 7.82–7.88 (m, 4H H, 2H, BT + 2H Ar), 7.91–7.93 (3H, Ar) 8.38 (s, 1H, BT). ^13^C NMR (101 MHz, CDCl_3_): δ 122.9 (C_BT_), 125.0 (C_BT_), 125.3 (Ar), 126.1 (Ar), 126.9 (Ar), 127.4 (Ar), 127.9 (Ar), 128.3 (Ar), 128.6 (Ar), 129.4 (Ar), 130.7 (2 δ Ar), 132.2 (C_BT_), 132.3 (CBT), 135.1 (C_BT_), 135.3 (C_BT_), 139.0 (C_BT_), 142.7 (C_BT_), 143.2 (C_BT_), 189.6 (C=O). Measured monoisotopic mass [C_19_H_12_O^32^S+H]^+^: *m*/*z* 289.0682 (Δ: 0.15 ppm)—Theoretical monoisotopic mass: *m*/*z* 289.06816.

### 2.3. Biological Assays

#### 2.3.1. Determination of IC_50_ Values

IC_50_ values for the inhibition of *h*MAO-A and *h*MAO-B were determined according to a previously reported protocol using commercially available recombinant enzymes as the enzyme sources [45]. Kynuramine was employed as the substrate for both *h*MAO isoforms. Its oxidation by *h*MAOs produces 4-hydroxyquinoline, which was quantified by fluorescence spectrophotometry.

*h*MAO activities were evaluated in the presence of varying concentrations of inhibitors (0.003–100 µM), and IC_50_ values were calculated accordingly. The enzyme reactions (200 µL) were performed in white 96-well microtiter plates (Eppendorf) using potassium phosphate buffer (100 mM, pH 7.4) and contained kynuramine (50 µM), test inhibitors spanning at least three orders of magnitude (0.003–100 µM), and either *h*MAO-A (0.0075 mg protein/mL) or *h*MAO-B (0.015 mg protein/mL). Reactions were initiated by enzyme addition, incubated for 20 min at 37 °C, and terminated by the addition of 2N NaOH (80 µL). The fluorescence intensity of 4-hydroxyquinoline was measured at an excitation wavelength of 310 nm and an emission wavelength of 400 nm. Sigmoidal plots of catalytic activity versus the logarithm of inhibitor concentration were generated, and IC_50_ values were determined and expressed as the mean ± standard deviation (SD) of triplicate measurements.

#### 2.3.2. Lineweaver–Burk Plots

The Lineweaver–Burk plots were constructed by employing *h*MAO-A and *h*MAO-B at the concentrations of 0.0075 mg protein/mL and 0.015 mg protein/mL, respectively, and a final volume of 500 μL. The first plot was obtained in the absence of inhibitor, while the remaining five plots were constructed in the presence of the following concentrations: ¼ × IC_50_, ½ × IC_50_, ¾ × IC_50_, 1 × IC_50_ and 1¼ × IC_50_. For each experimental condition, kynuramine was applied over a concentration range of 15–250 μM. The inhibition constant (*K*_i_) was determined by plotting the slopes derived from Lineweaver–Burk analyses against inhibitor concentration, with the *x*-axis intercept corresponding to −*K*_i_ [46].

#### 2.3.3. Cell Culture

The human gingival fibroblast (*h*GF) cell line was acquired from ATCC (PCS-201-018 ATCC, Manassas, VA, USA) and cultured with Fibroblast Basal Medium (485 mL; ATCC PCS-201-030) included in the growth kit (ATCC PCS-201-041) [47,48]. The human neuroblastoma cell line (SH-SY5Y) was purchased from ATCC (ATCC^®^ No. CRL-2266) and cultured with Dulbecco’s Modification of Eagle’s Medium with 1 g/L glucose, L-glutamine, sodium pyruvate and 10% of Fetal Bovine Serum. Both cell lines were maintained in an incubator at 37 °C with 5% CO_2_ and 95% air. When cells reached 75–80% confluence, they were split to obtain subcultures [39].

#### 2.3.4. Cell Metabolic Activity

MTS assay (CellTiter 96^®^ Aqueous One Solution Cell Proliferation Assay, Promega, Madison, WI, USA) [49,50,51] was employed to assess the cell metabolic activity of *h*GF and SH-SY5Y cells. Specifically, 2.4 × 10^3^ *h*GF/well and 5× 10^4^ SH-SY5Y/well were seeded in a 96-multiwell plate and maintained in culture medium in an incubator for 24 h. *h*GF and SH-SY5Y cells were exposed to compounds **4**, **11**, and **12**, as well as (*R*)-(−)-Deprenyl, employed as a control, at final concentrations of 12.5, 25, 50, and 100 µM for a 24 h period. Subsequently, 20 µL of MTS reagent was added to each well, and the plates were incubated at 37 °C for an additional 3 h. Cell metabolic activity, proportional to cell viability, was quantified by measuring absorbance at 490 nm using a Synergy™ HT multi-mode microplate reader (BioTek, Winooski, VT, USA) [36,50].

An identical procedure was applied to evaluate the viability of SH-SY5Y cells treated with 6-hydroxydopamine (6-OHDA, 500 µM, Merck, Darmstadt, Germany) either alone or in combination with compounds **4**, employed at 12.5 µM concentration, and **11**, **12** or (*R*)-(−)-Deprenyl (100 µM). Co-treatments were carried out for 12 h and 24 h.

#### 2.3.5. Statistical Analysis

Statistical significance was established with GraphPad 5.01 (GraphPad, San Diego, CA, USA) software utilizing one-way ANOVA followed by post hoc Tukey’s multiple comparisons analysis. Values of *p* < 0.05 were considered statistically significant.

#### 2.3.6. ROS Assessment and Quantification

ROS levels were evaluated using confocal microscopy (Zeiss LSM 800, Carl Zeiss, Jena, Germany) with the specific probe 2′,7′-dichlorodihydrofluorescein diacetate (H2DCF-DA, Thermo Fisher Scientific) [52]. SH-SY5Y cells were incubated in Normal Esternal Solution (NES: 140 mM NaCl, 2.8 mM KCl, 2 mM CaCl_2_, 2 mM MgCl_2_, 10 mM glucose, 10 mM Hepes, pH 7.3) for 40 min at 37 °C with 10 µM H2DCF-DA or for 15 min at 37 °C. Fluorescence signals for H2DCF-DA were acquired, setting excitation at 488 nm and emission at 535 nm. For each sample, 5–7 randomly selected fields were acquired. Fluorescence intensity was quantified using Fiji Image J software (version 1.54). The ROS levels were calculated by outlining the regions corresponding to the cell area using ROIs in the acquired fields. For each cell, the mean fluorescence intensity value, expressed as arbitrary units (F), and area were calculated; the data were expressed as mean fluorescence per area unit (F/μm^2^).

### 2.4. Molecular Modelling Studies

The X-ray structures of *h*MAO-A in complex with harmine (PDB code 2Z5X) [2] and *h*MAO-B in complex with a monosubstituted chalcone (PDB code 7B0V) [53] were downloaded from the Protein Data Bank and used in these studies [54]. The structures of the analyzed compounds were built using MolBook UNIPI software (version 1.0) [55], and were then subjected to docking calculations, which were carried out with AUTODOCK 4.2 [56] using a robust procedure already applied in pose prediction analyses [57]. Autodock Tools were used to assess the torsion angles in the ligands, add the solvent model and assign the Kollman atomic charges to the proteins, whereas ligand charges were calculated with the Gasteiger method. A grid spacing of 0.375 Å and a distance-dependent function of the dielectric constant were used for the energetic map calculations. Each docked compound was subjected to 200 runs of the AUTODOCK search using the Lamarckian Genetic Algorithm, performing 10,000,000 steps of energy evaluation. The number of individuals in the initial population was set to 500, and a maximum of 10,000,000 generations were simulated during each docking run. All other settings were left as their defaults, and the best docked conformation belonging to the best cluster of solutions was considered for each ligand in each docking study. The predicted ligand–protein complexes were then refined through molecular dynamics (MD) simulations, which were performed with Amber 22. All MD simulations were carried out using the ff14SB force field for the protein, while GAFF (General Amber force field) was used for the ligands, whose partial charges were calculated using the AM1-BCC method through the antechamber suite of AMBER 22. The complexes were included in a parallelepiped box and solvated with a 15 Å water cap using the TIP3P explicit solvent model, while sodium ions were added as counterions to neutralize the systems. Prior to the MD simulations, 5000 steps of energy minimization, including 2000 steps of steepest descent (SD) followed by 3000 steps of conjugate gradient (CG), were initially performed, applying a position restraint of 10 kcal/mol·Å^2^ on the protein α-carbons, for energy minimization of the entire systems. The energy-minimized systems were used as input for an MD simulation protocol adapted from previous studies [58], in which particle mesh Ewald (PME) electrostatics and periodic boundary conditions were used, with a cut-off of 10 Å for the non-bonded interactions. A constant-volume MD simulation was carried out for 1 ns, during which the temperature of the systems was increased from 0 to 300 K. The systems were then pre-equilibrated through 5 ns of constant-pressure simulation, which was carried out while keeping the temperature at the constant value of 300 K by using the Langevin thermostat. A time step of 2.0 fs was employed in the simulations since all bonds involving hydrogen atoms were kept fixed using the SHAKE algorithm. A second-long constant-pressure equilibration stage of 500 ns was then performed at 300 K to reach an optimal relaxation of the protein binding site conformation and full equilibration. This second equilibration stage was performed using the hydrogen mass repartition (HMR) scheme [59] and a time step of 4.0 fs, since this technique proved to be useful for reducing the simulation time while maintaining an unbiased MD protocol [60]. Both the heating and the two equilibration stages were performed, maintaining a position restraint of 10 kcal/mol·Å^2^ on the protein α-carbons. The fully equilibrated systems were then subjected to 500 ns of constant-pressure MD at 300 K, maintaining the HMR scheme and a time step of 4.0 fs, in which no restraint was applied to the protein, thus leaving the whole systems free. The average structures obtained from the last 250 ns of the MD trajectories were generated using the Cpptraj program implemented in AMBER 22.

## 3. Results and Discussion

### 3.1. Chemistry

**Scheme 1:**
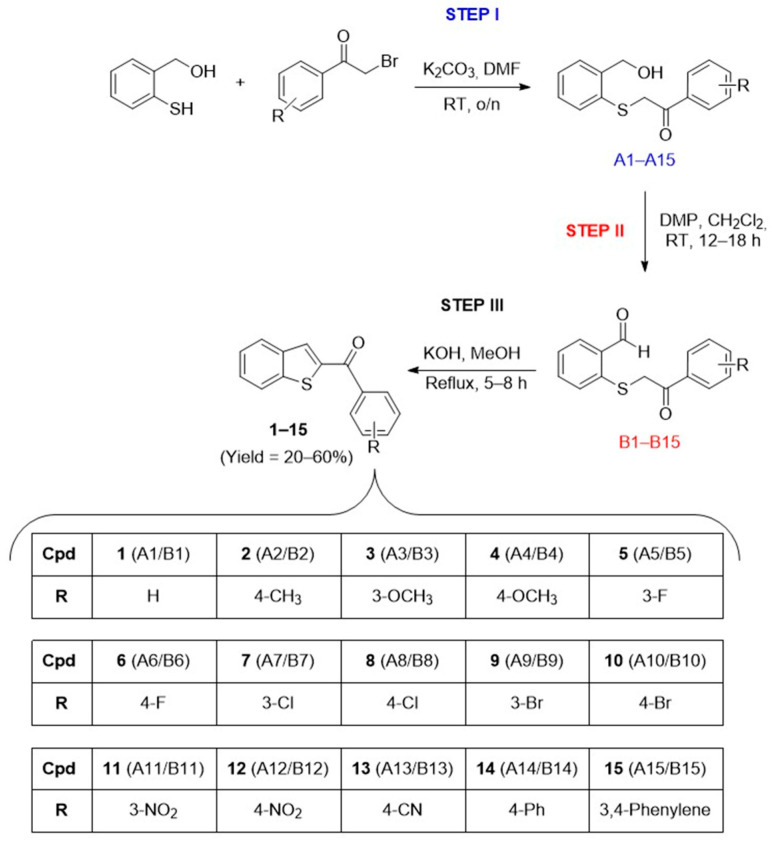
Multi-step synthetic approach employed for the synthesis of compounds **1**–**15**.

For the synthesis of compounds **1**–**15**, we employed a three-step synthetic approach inspired by the procedure reported by Hsiao and colleagues [61], with some modifications (Scheme 1). The overall strategy was designed to generate aldehyde intermediates B1–B15, which underwent an intramolecular aldol reaction to yield the final 2-aroylbenzofuran analogues **1**–**15** (Scheme 1, STEP I–III). The first step involved the synthesis of alcohol intermediates **A1**–**A15** via the reaction between 2-mercaptobenzyl alcohol and the appropriate (un)substituted α-bromoacetophenone in dry *N*,*N*′-dimethylformamide (DMF) at room temperature (r.t.), using potassium carbonate (K_2_CO_3_) as a base (Scheme 1, Step I). A liquid–liquid extraction afforded the alcohols in good yields and purity, without the need for further purification. These intermediates were then selectively oxidized to the corresponding aldehydes **B1**–**B15** using Dess–Martin Periodinane (DMP) in dichloromethane at room temperature (Scheme 1, Step II, [62,63]). The final step involved an intramolecular aldol reaction in refluxing methanolic potassium hydroxide solution, affording the title compounds **1**–**15** in moderate to high yields after purification (Scheme 1, Step III). The synthesized compounds were all structurally characterized through ^1^H-NMR, ^13^C-NMR and HR-MS before their evaluation in the in vitro assays (further details are provided in the Section 2 and/or the Supplementary Materials).

### 3.2. Biological Assays

#### 3.2.1. *h*MAO-A and *h*MAO-B Inhibition Studies

The inhibitory activity of compounds **1**–**15**, expressed as IC_50_ values, was evaluated against both *h*MAO-A and *h*MAO-B and is summarized in Table 1. Selectivity index (SI) values, calculated as the ratio (IC_50_ *h*MAO-A)/(IC_50_ *h*MAO-B), are also presented in Table 1.

Globally, these compounds preferentially inhibited *h*MAO-B, the few exceptions to this general trend being compounds **1** and **13**, endowed with an unsubstituted phenyl ring and a *para*-CN-substituted phenyl ring, respectively (Table 1). Another general observation is that these derivatives were more potent than the 2-aroylbenzothiophen-3-ols (Table S1) [38], but less effective than the corresponding 2-aroylbenzofuran analogues (Table S2) [39], although the actual comparison with benzofuran’s inhibitory data cannot be performed due to the employment of different methods for IC_50_ determination [39]. However, it is evident that the removal of the 3-hydroxyl group from the benzothiophene core of the 2-aroylbenzo[*b*]thiophen-3-ol scaffold dramatically improved the activity, an intriguing behaviour that closely mirrors the trend observed between 2-aroylbenzofuran-3-ols and 2-aroylbenzofurans [39].

Analysis of the structure–activity relationship (SAR) of the 2-aroylbenzothiophenes indicates that structural modifications, especially functional group variations, markedly affect both potency and selectivity. The simplest compound of the series (compound **1**) is characterized by micromolar affinity against both *h*MAO isoforms with a negligible preference for *h*MAO-A (IC_50_ for *h*MAO-A = 2.46 µM; IC_50_ for *h*MAO-B = 2.71 µM; SI = 0.91). The introduction of electron-donating substituents, such as methyl or methoxy groups on the 2-aroylphenyl ring, enhanced *h*MAO-B inhibition (Table 1). Notably, compound **4**, bearing the *para*-methoxy group (IC_50_ for *h*MAO-B = 0.057 µM), was three times more potent than compound **2**, which featured a *para*-methyl substituent (IC_50_ for *h*MAO-B = 0.177 µM). This effect correlates with the electronic properties of the substituents, as reflected by their negative Hammett *σ* constants, with a stronger electron-donating group (*σ_para_* OCH_3_ = −0.27) producing a greater increase in inhibitory potency than a weaker donor (*σ_para_* CH_3_ = −0.16). Overall, both substituents had a minimal impact on *h*MAO-A inhibition (Table 1). Indeed, compared to derivative **1**, the *para*-methyl derivative **2** demonstrated a slight increase in affinity for the *h*MAO-A isoform (IC_50_ for *h*MAO-A = 2.13 µM), whereas the *para*-methoxy analogue **4** exhibited a modest reduction in inhibitory activity against the same isoform (IC_50_ for *h*MAO-A = 2.59 µM). These outcomes affected the SI value, which was higher for compound **4** (SI = 45.4) compared to analogue **2** (SI = 12.0). Interestingly, the effect of the methoxy group differed when located in the *meta* position (derivative **3**), resulting in enhanced *h*MAO-A inhibition (IC_50_ for *h*MAO-A = 1.38 µM) but reduced *h*MAO-B affinity (IC_50_ for *h*MAO-B = 0.325 µM), thus further reducing the SI value (SI = 4.0). This behaviour can be rationalized by the electronic properties of the methoxy substituent in the *meta* position, where resonance donation is ineffective and the inductive electron-withdrawing effect predominates (*σ_meta_* OCH_3_ = +0.11).

Even for the electron-withdrawing substituents, a tight correlation between electronic properties and influence on inhibitory activity and selectivity was observed (Table 1). Halogens are intriguing substituents that exhibit a complex interplay between a pronounced electron-withdrawing inductive effect, which is slightly offset by a weaker electron-donating resonance arising from lone-pair donation to aromatic systems. In general, the presence of a halogen on the 2-aroylphenyl ring (compounds **5**–**10**) enhanced *h*MAO-B inhibition compared to the unsubstituted compound **1**. Notably, halogen substitution at the *meta* position (compounds **5**, **7** and **9**) resulted in greater *h*MAO-B inhibition compared to their *para*-substituted counterparts (derivatives **6**, **8** and **10**). This trend was less pronounced in the case of fluoro-substituted compounds (**5** and **6**). Although fluorine displays a markedly stronger electron-withdrawing effect in the *meta* compared to the *para* position (*σ_meta_* F = +0.34; *σ_para_* F = +0.05), this difference did not translate into a significant variation in *h*MAO-B inhibition, suggesting that electronic effects alone do not govern activity among the halogen-substituted analogues. The analysis of the IC_50_ values for 2-aroylbenzothiophenes *meta*-substituted with halogens revealed a clearer trend, with *h*MAO-B affinity increasing with halogen size and polarizability. Indeed, the *meta*-fluoro-substituted derivative **5** (IC_50_ for *h*MAO-B = 0.431 µM, *σ_meta_* F = +0.34) was less effective than its *meta*-chloro analogue, compound **7** (IC_50_ for *h*MAO-B = 0.187 µM, *σ_meta_* Cl = +0.37), which in turn was less potent than compound **9**, featuring a *meta*-bromo substitution (IC_50_ for *h*MAO-B = 0.131 µM, *σ_meta_* Br = +0.40). Given the relatively similar *σ_meta_* values of Cl and Br, this trend is more plausibly attributed to steric and hydrophobic factors rather than to electronic effects alone. This behaviour was not observed for derivatives bearing halogens in the *para* position, among which the chloro-substituted analogue **8** (IC_50_ for *h*MAO-B = 0.266 µM) was slightly more potent than the *para*-bromo **10** (IC_50_ for *h*MAO-B = 0.378 µM) and *para*-fluoro **6** (IC_50_ for *h*MAO-B = 0.483 µM) derivatives, despite the comparable electron-withdrawing inductive effects of Cl (*σ_para_* Cl = +0.22) and Br (*σ_para_* Br = +0.23) in the *para* position. Halogen substitution also influenced *h*MAO-A inhibition in a position-dependent manner. Globally, the presence of a halogen in the *meta* position led to impairment of affinity against *h*MAO-A, with high micromolar inhibition for *meta*-chloro **7** (IC_50_ for *h*MAO-A = 9.54 µM) and *meta*-bromo **9** (IC_50_ for *h*MAO-A = 9.54 µM), thus contributing to high SI values (**7**, SI = 51.0; **9**, SI = 61.2), while fluorine was better tolerated in this position (**5**, IC_50_ for *h*MAO-A = 1.71 µM). In contrast, when placed in the *para* position, fluorine led only to micromolar inhibition of *h*MAO-A (**9**, IC_50_ = 5.76 µM), whereas *para*-chloro and *para*-bromo substituents exerted comparable and positive effects on *h*MAO-A inhibition, yielding sub-micromolar IC_50_ values and consequently less selective compounds (**8**, IC_50_ = 0.933 µM; **10**, IC_50_ = 0.948 µM). Continuing the discussion on the effects of electron-withdrawing substituents, the introduction of a nitro group at either the *meta* or *para* position led to a clear increase in potency against *h*MAO-B (Table 1). Indeed, compounds **11** and **12**, bearing *meta*- and *para*-nitro substituents, respectively, exhibited *h*MAO-B inhibition in the nanomolar range, with the *meta*-substituted derivative (**11**, IC_50_ for *h*MAO-B = 0.021 µM) being more potent than its *para*-substituted counterpart (**12**, IC_50_ for *h*MAO-B = 0.045 µM). Concerning *h*MAO-A inhibition, while compound **11** displayed sub-micromolar inhibitory activity (**11**, IC_50_ for *h*MAO-A = 0.918 µM; SI = 43.7), compound **12** exhibited similar potency against *h*MAO-A (**12**, IC_50_ for *h*MAO-A = 0.069 µM), thus contributing to the scarce selectivity of this compound (**12**, SI = 1.4). The Hammett σ constants for the nitro group are very similar at the *meta* and *para* positions *(σ*_meta_ NO_2_ = +0.73 and *σ*_para_ NO_2_ = +0.78). Accordingly, the differences observed, particularly in *h*MAO-A inhibition, cannot be attributed solely to the electronic effects of the substituent, with steric hindrance also playing a significant role. Similar behaviour was observed with the *para*-CN analogue (**13**), albeit exerting a relatively minor electron-withdrawing inductive effect (*σ*_para_ CN = +0.67), which showed comparable inhibitory activity against both the isoforms (**13**, IC_50_ for *h*MAO-A = 0.064 µM; IC_50_ for *h*MAO-B = 0.068 µM) and a negligible preference for the A isoform (**13**, SI = 0.94).

The insertion of bulky groups yielded opposing results. The presence of the *para*-phenyl substituent (**14**), characterized by an extended aromatic surface but limited three-dimensional bulkiness (low B_1_ and B_5_ Sterimol parameters [65]), was poorly tolerated, leading to micromolar inhibition for both *h*MAO isoforms. On the contrary, the presence of the 2-naphthyl moiety, which combines increased steric volume and greater molecular width (higher B_5_ and overall steric bulkiness), resulted in the most selective *h*MAO-B inhibitor (**15**, SI = 65.4). This high selectivity stemmed from a significant reduction in *h*MAO-A affinity (IC_50_ = 15.9 µM), despite potent inhibition of *h*MAO-B (IC_50_ for *h*MAO-B = 0.243 µM).

**Figure 2:**
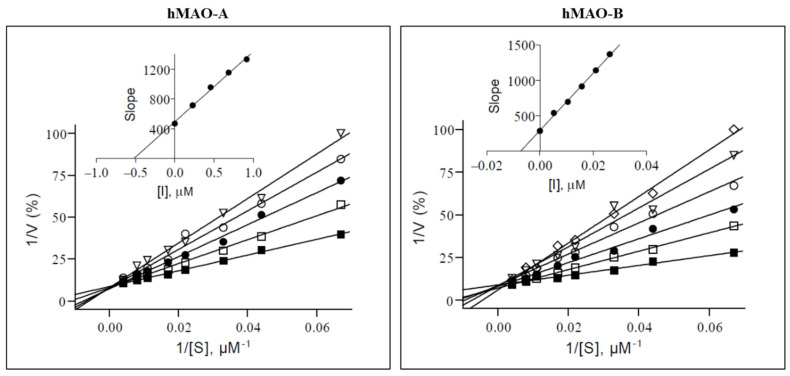
Lineweaver–Burk plots for the inhibition of *h*MAO-A (**left**) and *h*MAO-B (**right**) by compound **11**. The insets are replots of the slopes of the Lineweaver–Burk plots vs. inhibitor concentration from which enzyme–inhibitor dissociation constants (*K*_i_) were estimated as 0.52 and 0.0072 µM for *h*MAO-A and *h*MAO-B, respectively.

The inhibition profile of compound **11** (R = 3-NO_2_), which showed the best activity on *h*MAO-B, with an IC_50_ value of 21 nM and moderate activity on *h*MAO-A (IC_50_ = 0.918 µM), has been further evaluated by Lineweaver–Burk plots. From the graph (Figure 2), it appears that the slope of the curve with no inhibitor is lower than those of the curves with increasing inhibitor concentrations. Furthermore, the y-intercept is approximately the same for all curves, meaning that V_max_ (at an infinite substrate concentration (1/[S] = 0) the substrate molecules occupy all the available enzymes) does not depend on the substrate concentration. This profile is characteristic of competitive inhibitors, which bind in the same place as the substrate, thus competing for binding with the enzyme [66]. As the substrate concentration increases, it forces the ligand out of the active site. The type of enzyme inhibition evaluated for **11** cannot be automatically applied to all the other compounds, which must be characterized in an analogous way. When plotting the slopes obtained from the linear regressions from the Lineweaver–Burk plot as a function of the inhibitor concentration, a linear curve is obtained. From the analysis of this latter secondary plot, *K*_i_ can be obtained as the X-intercept, and the values obtained are 520 nM and 7.2 nM for *h*MAO-A and -B, respectively.

#### 3.2.2. Cell Metabolic Activity: Effects of *h*MAO-A and *h*MAO-B Inhibitors on *h*GF and SH-SY5Y Cell Lines

**Figure 3:**
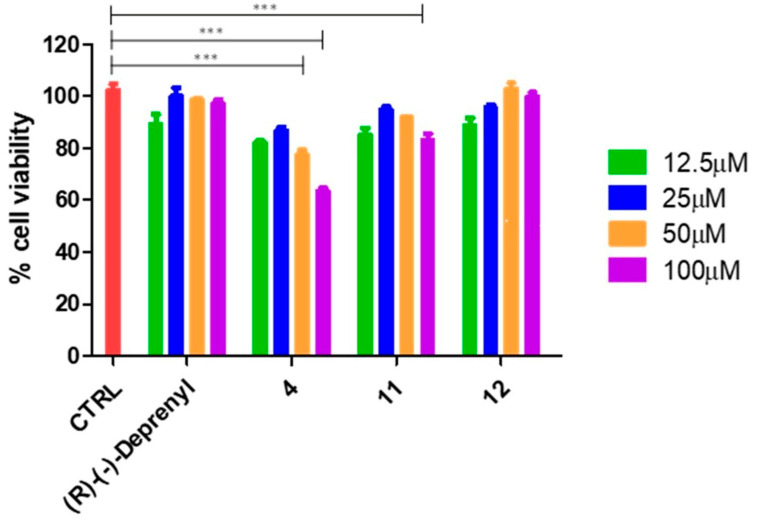
Cell viability evaluated using MTS assay and normalized to control cells treated with DMSO (0.2% as final concentration). Histograms represent viability dose–response of *h*GF cells exposed to **4**, **11** and **12**, (*R*)-(−)-Deprenyl at 12.5 µM, 25 µM, 50 µM and 100 µM for 24 h. *** *p* < 0.001.

**Figure 4:**
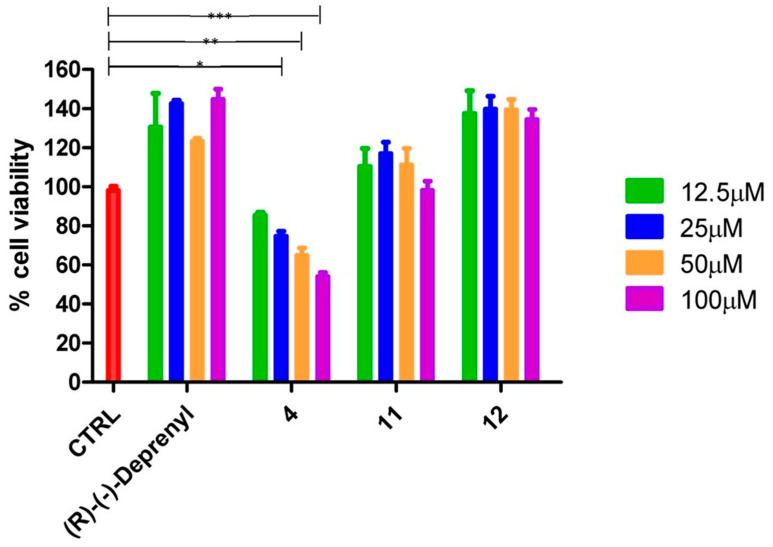
Cell viability evaluated using MTS assay and normalized to control cells treated with DMSO (0.2% as final concentration). Histograms represent viability dose–response of SH-SY5Y cells exposed to compounds **4**, **11** and **12**, (*R*)-(−)-Deprenyl at 12.5 µM, 25 µM, 50 µM and 100 µM for 24 h. * *p* < 0.05, ** *p* < 0.01, *** *p* < 0.001.

The compounds exhibiting the most promising results (**4**, **11** and **12**) were further investigated in cellulo. Whereas derivatives **4** and **11** exhibited potent and selective inhibition of *h*MAO-B, compound **12** effectively inhibited both isoforms (Table 1). With the aim of evaluating the effects of compounds **4**, **11** and **12** on cell viability, MTS assays were performed. In detail, we evaluated the selected compounds on human gingival fibroblast (*h*GF) and human neuroblastoma (SH-SY5Y) cell lines [39]. Derivatives **4**, **11** and **12** were tested individually and compared to the clinically available *h*MAO-B inhibitor (*R*)-(−)-Deprenyl, employed as a positive control. *h*GFs and SH-SY5Y cells were incubated for 24 h with the test compounds at concentrations between 12.5 and 100 μM (Figure 3 and Figure 4). In *h*GFs (Figure 3), compound **4** exhibited a significant decrease in terms of cell viability at 50 and 100 µM, evidencing reductions of ≃40% and 20%, respectively. Similarly, a significant reduction in terms of cell viability could be observed for compound **11** at 100 µM, showing a reduction of ≃15% after 24 h of treatment. On the other hand, the non-selective compound **12** was well tolerated, preserving cellular viability up to 100 µM.

In SH-SY5Y cells (Figure 4), compound **4** showed similar behaviour by inducing a significant decrease in cell viability at 100, 50, and 25 µM, while it was better tolerated at 12.5 µM, although it still exhibited slight cytotoxicity. Instead, none of the other tested compounds, such as **11** and **12**, affected cell viability in SH-SY5Y after 24 h of treatment at the tested concentrations.

#### 3.2.3. ROS Assessment

**Figure 5:**
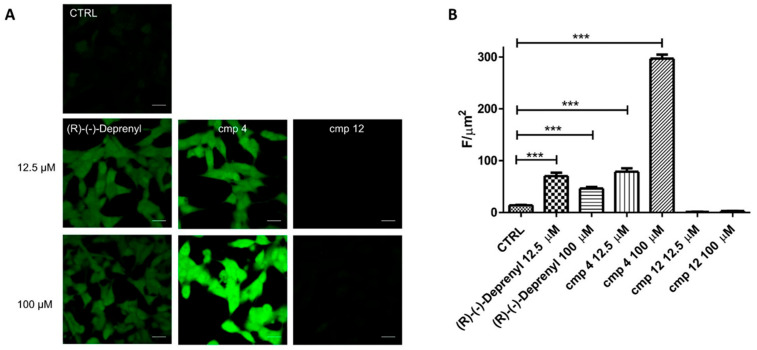
ROS assessment in SH-SY5Y. (**A**) Representative images under tested conditions. (**B**) Quantitative analysis of ROS production calculated as arbitrary units of fluorescence per cell surface unit (F/µm^2^). Data are presented as mean ± S.E.M (CTRL; cmp **4** (12,5 µM); cmp **12** (100 µM); cmp **12** (100 µM); cmp **12** (12.5 µM); (*R*)-(−)-Deprenyl (100 µM) and (*R*)-(−)-Deprenyl (12.5 µM). Control cells (CTRL) treated with DMSO (0.2% as final concentration). *** *p* < 0.001. Scale bar: 20 μm.

To elucidate one of the possible mechanisms responsible for the divergent behaviours of compound **4** compared to **11** and **12** in the SH-SY5Y cell viability assay, we conducted a ROS assessment. The ROS production was quantified using an approach based on live imaging in confocal microscopy using H2DCFDA-loaded SH-SY5Y. Compounds **4** and **12** were selected for this study, being the most toxic and the least toxic, respectively. (*R*)-(−)-Deprenyl was also included as a reference drug. Images in panel A of Figure 5 show representative images of the results obtained after treatment with (*R*)-(−)-Deprenyl and compounds **4** and **12**, both at 12.5 µM and at 100 µM. In the control condition (CTRL), cells exhibit very low fluorescence, reflecting baseline production. Treatment with (*R*)-(−)-Deprenyl at 12.5 µM leads to a clear increase in fluorescence, which does not differ from that at 100 µM. A different trend is observed with compound **4**, which also increases fluorescence at both concentrations, indicating a dose-dependent effect. On the other hand, cells treated with derivative **12** do not show any visible increase in fluorescence, regardless of concentration, suggesting that this compound has no effect on ROS production under the tested conditions. In Figure 5 panel B we quantify the fluorescence intensity in terms of F/µm^2^ to support the observations from the microscopy images. In summary, the results demonstrate that the tested compound **4** is the most effective in enhancing ROS production, followed by (*R*)-(−)-Deprenyl, while compound **12** appears to have no biological activity in this assay. These findings suggest that compound **4** may modulate specific cellular processes potentially linked to mitochondrial function or oxidative stress response that are not affected by compound **12**. The significant enhancement in ROS production upon treatment with compound **4** in a dose-dependent manner could partially explain the observed reduced cellular viability of SH-SY5Y cells (Figure 4).

Interestingly, compounds **4** and **12** showed opposite behaviours, which appears to contrast with the general notion that antioxidant effects are often favoured by electron-donating substituents, such as the 4-OCH_3_ group in compound **4** (*σ_para_* OCH_3_ = −0.27), rather than by electron-withdrawing groups, such as 4-NO_2_ in derivative **12** (*σ*_para_ NO_2_ = +0.78) [67,68,69]. However, this trend is typically established using dedicated chemical antioxidant assays, which probe the direct radical-scavenging/redox behaviour of compounds and therefore their intrinsic redox properties. In the present work, ROS levels were assessed in a cellular setting and must be interpreted in the context of a complex biological system, where the readout reflects broader downstream effects on intracellular ROS formation. Accordingly, although the ROS outcomes did not correlate with substituent electronic properties (Hammett *σ* constants), they are more plausibly explained by broader cellular effects, including the distinct MAO inhibition profiles. Indeed, while this interpretation remains speculative, the less selective compound **12** produced a greater overall reduction in ROS than the more *h*MAO-B-selective compound **4**, possibly because it more effectively limits H_2_O_2_ production by inhibiting both MAO isoforms rather than *h*MAO-B alone.

The ability of compounds to reduce ROS production, and more generally to counteract oxidative stress, is an important feature of candidate neuroprotective agents. Indeed, oxidative stress is widely recognized as a key contributor to the pathogenesis of neurodegenerative disorders, particularly Parkinson’s disease, by promoting mitochondrial dysfunction and the progressive degeneration of dopaminergic neurons [43,70,71]. Oxidative stress arises from an imbalance between the generation of reactive oxygen species (ROS) and the capacity of biological systems to detoxify these reactive intermediates [72]. As mentioned above, oxidative deamination catalyzed by *h*MAOs leads to the formation of hydrogen peroxide (H_2_O_2_), which is normally detoxified by enzymes such as catalase and glutathione peroxidase [73]. However, increased *h*MAO activity, particularly of the B isoform during ageing [74], can contribute both to reduced dopamine levels and to excessive H_2_O_2_ production that overwhelms cellular detoxification mechanisms, thereby promoting oxidative stress. Moreover, in the presence of reduced metals such as ferrous iron (Fe^2+^), H_2_O_2_ can participate in the well-known Fenton reaction, generating the highly reactive hydroxyl radical, one of the most damaging ROS [75].

#### 3.2.4. Cell Viability of SH-SY5Y Cells Treated with 6-OHDA

**Figure 6:**
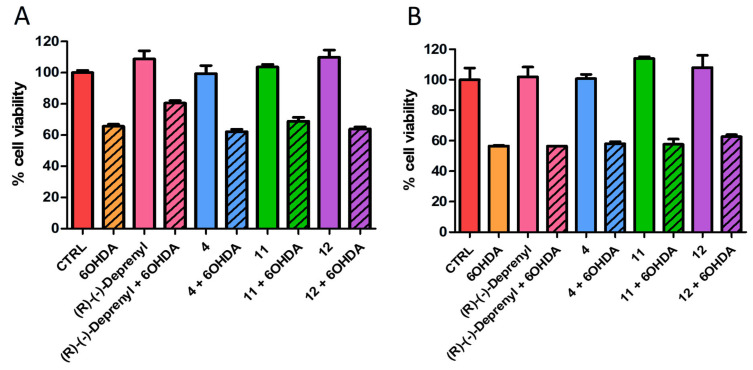
Cell viability using MTS assay and normalized to control cells treated with DMSO (0.2% as final concentration). (**A**) Histograms represent cell viability of 6-OHDA-treated SH-SY5Y cells (500 µM) alone or co-incubated with (*R*)-(−)-Deprenyl, **11** and **12** at 100 µM and with **4** at 12.5 µM for 12 h. (**B**) Histograms represent the cell viability of 6-OHDA-treated SH-SY5Y cells (500 µM) alone or co-incubated with (*R*)-(−)-Deprenyl, **11** and **12** at 100 µM and with **4** at 12.5 µM for 24 h.

Compound **4** at a concentration of 12.5 μM and derivatives **11**, **12** and (*R*)-(−)-Deprenyl at a concentration of 100 μM were evaluated in co-treatment with 500 μM 6-OHDA (Figure 5), used as a model to recapitulate PD [39,76,77,78]. Interestingly, after 12 h of co-treatment with 6-OHDA, only (*R*)-(−)-Deprenyl evidenced an improved cell viability of the SH-SY5Y cell line (≃15%) compared to 6-OHDA alone (Figure 6). In contrast, after 24 h of exposure, all evaluated compounds failed to mitigate 6-OHDA-induced toxicity. Only compound **12** exhibited a slight protective tendency, although this effect did not achieve statistical significance (Figure 6).

**Figure 7:**
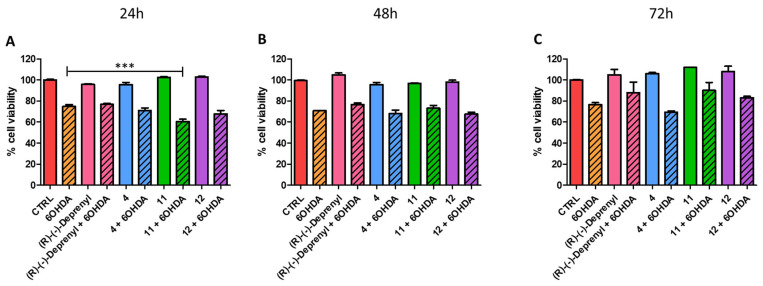
Cell viability using MTS assay and normalized to control cells treated with DMSO (0.2% as final concentration). (**A**–**C**) Histograms represent cell viability of 6-OHDA-treated SH-SY5Y cells (250 µM) alone or co-incubated with (*R*)-(−)-Deprenyl, **11** and **12** at 100 µM and with **4** at 12.5 µM for 24 h, 48 h and 72 h. *** *p* < 0.001.

In light of the results obtained upon co-treatment with 6-OHDA at 500 µM for 24 h, we further investigated whether these compounds could counteract 6-OHDA-induced neurotoxicity under different conditions. Specifically, we used a sub-toxic concentration of 6-OHDA (250 µM) and extended the observation period to 72 h, while keeping the concentrations of the tested compounds unchanged (compound **4** at 12.5 µM; compounds **11** and **12** and (*R*)-(−)-Deprenyl at 100 µM). Consistent with our previously reported work, after 24 h, the compounds failed to mitigate 6-OHDA-induced toxicity; a similar outcome was also observed for the previously reported *p*-NO_2_ benzofuran analogue **BF20** [39] included to compare the efficiency of the two different chemical scaffolds (Figure S1). Interestingly, although all compounds showed comparable effects after 48 h, at the longer observation time (i.e., 72 h) compounds **11** and **12** displayed a slight protective trend, comparable to that of the clinically used drug (*R*)-(−)-Deprenyl (100 μM), although this did not reach statistical significance (Figure 7 and Figure S1).

### 3.3. Molecular Modelling Studies

**Figure 8:**
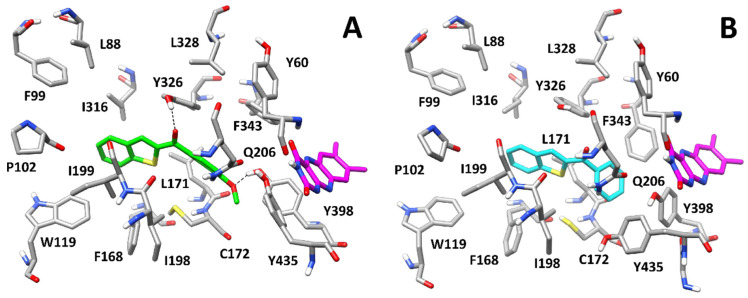
Predicted binding modes of compounds **4** (**A**) and **1** (**B**) with *h*MAO-B. Compounds **4** and **1** are shown in green and cyan, respectively. For clarity, only the flavin group of the cofactor is shown in magenta. Ligand–protein H-bonds are shown as black dashed lines.

With the aim of predicting the potential binding mode of this series of compounds, molecular modelling studies including docking and molecular dynamics (MD) simulations were performed. Compound **4**, which showed one of the best activities against *h*MAO-B (IC_50_ = 0.057 µM) and selectivity over *h*MAO-A (SI = 45.4) of the whole series, was used as a reference for these studies. Its binding mode was compared to the one proposed for the parent unsubstituted compound **1**, which weakly inhibited both isoforms in the micromolar range (see Table 1). Both compounds were docked into the X-ray structures of the two *h*MAO isoforms using a robust docking procedure, and the generated binding poses were then refined through a long MD simulation protocol (see Materials and Methods for details). Figure 8A shows the binding modes predicted for compound **4** with *h*MAO-B based on the whole molecular modelling protocol. The ligand is anchored to the binding site of the protein through a H-bond formed between its carbonyl group and the side chain of Y326, which is placed in the middle of the binding cavity. The benzothiophene moiety of compound **4** is placed in a lipophilic pocket located in the terminal portion of the binding site, mainly delimited by P102, W119, F168, L171, I199, I316 and Y326, forming hydrophobic interactions with all these residues, particularly with I199 and I316, between which the terminal aromatic ring of the ligand is sandwiched. The methoxyphenyl group of **4** is oriented towards the flavin group of the cofactor and forms hydrophobic interactions mainly with L171, C172, Y398, and Y435. Finally, a H-bond is observed between the methoxyl group of the ligand and the side chain of Y398. Although such interaction does not appear to be particularly stable during the simulation, likely due to the conjugation of the methoxyl group of the ligand with the adjacent benzoyl moiety, it certainly contributes to stabilizing the ligand in the binding pocket of *h*MAO-B and to increasing its inhibitory activity against it. As displayed in Figure 8B, although compound **1** assumes a similar orientation with *h*MAO-B to that predicted for **4**, the ligand shows key differences in its binding mode. In fact, due to the absence of the methoxyl substituent of **4**, compound **1** protrudes deeper towards the flavin group of the cofactor, forming extensive hydrophobic interactions with L171, C172, F343, Y398, and Y435. However, besides lacking the H-bond with Y398, the ligand cannot even form the H-bond with the side chain of Y326, being too far from this residue. Moreover, the benzothiophene moiety of **1** forms hydrophobic interactions with F168, L171, I199, and I316, but loses its contacts with P102 and W119. These results may explain the reduced activity of compound **1** against *h*MAO-B compared to compound **4** (2.71 µM vs. 0.057 µM, respectively).

**Figure 9:**
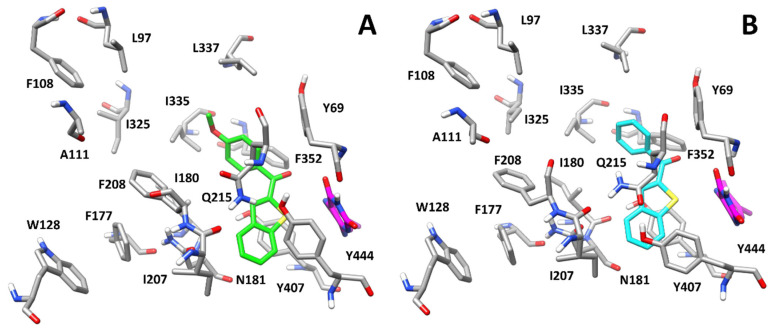
Predicted binding modes of compounds **4** (**A**) and **1** (**B**) with *h*MAO-A. Derivatives **4** and **1** are depicted in green and cyan, respectively. For clarity, only the flavin group of the cofactor is shown in magenta.

The binding mode predicted for compound **4** with *h*MAO-A (Figure 9A) is remarkably different from that obtained with *h*MAO-B. This is mainly due to the presence of two non-conserved residues that significantly change the shape of the binding cavity and the ligand anchoring points. In fact, the hydrophobic pocket located in the terminal portion of the binding site is mainly occupied by the side chain of F208, which replaces I199 of *h*MAO-B. Furthermore, the presence of I335 in place of Y326 in *h*MAO-B creates an open cavity in the upper part of the site, towards L97 and L337. Finally, the absence of Y326 prevents the ligand from forming the key H-bond observed in *h*MAO-B. For these reasons, the ligand shows a vertical orientation, compared to that shown in *h*MAO-B, with its benzothiophene moiety located next to the flavin group of the cofactor, forming π-π stacking with Y407 and Y444, as well as hydrophobic interactions with I207, N181 and the cofactor. The substituted phenyl ring of **4** is placed in the upper cavity of the binding site, forming hydrophobic interactions with Y69, I335 and F352. Finally, the terminal methoxyl group of the ligand forms neither H-bonds nor relevant hydrophobic interactions. The results agree with the reduced inhibitory potency of compound **4** against *h*MAO-A with respect to *h*MAO-B, and thus with its selectivity for *h*MAO-B. Interestingly, the binding mode predicted for compound **1** with *h*MAO-A (Figure 9B) is very similar to that of its derivative **4**. Accordingly, the ligand–protein interactions predicted for the two ligands are comparable. In particular, the benzothiophene group of compound **1** is placed between Y407 and Y444, with which it interacts through π-π stacking, and forms hydrophobic interactions with I207, N181 and the cofactor. Moreover, the ligand forms hydrophobic contacts with Y69, I335 and F352 through its phenyl ring, oriented in the upper cavity of the binding pocket. The results of this analysis may explain the comparable inhibitory activity of compounds **1** and **4** against *h*MAO-A (2.46 µM vs. 2.59 µM, respectively).

With the aim of rationalizing the influence of the position of the methoxy group of the ligands on the activity and selectivity, the same computational protocol, including docking and MD simulations, already performed to evaluate the binding mode of compounds **1** and **4** was also applied to compound **3**, the *m*-methoxy analogue of **4**. As shown in Figure S2, compound **3** was predicted to assume an orientation within *h*MAO-B catalytic site very similar to that of compound **4**, maintaining both the key H-bond with the phenolic group of Y326, established by its central carbonyl fragment, and all hydrophobic interactions with P102, W119, F168, L171, I199, I316 and Y326, formed by the bicyclic benzothiophene core. Nevertheless, the *m*-methoxyphenyl moiety of compound **3** cannot form the additional H-bond with the side chain of Y398 predicted for compound **4** (Figure 8A), being only able to establish hydrophobic interactions with L171, C172, I198, Y398, and Y435, which is consistent with the reduced activity of compound **3** against *h*MAO-B (IC_50_ = 0.325 µM) compared with that observed for compound **4** (IC_50_ = 0.057 µM). On the other hand, the binding mode with *h*MAO-A predicted for compound **3** (Figure S2B) is comparable to that of compound **4** (Figure 9A), as the same π-π stacking with Y407 and Y444 and hydrophobic interactions with I207, N181 and the cofactor are mainly formed by the ligand’s bicyclic core. The only slight difference may be constituted by the extended lipophilic contacts with I335 and L337 observed for the *m*-methoxyphenyl ring, which may explain the slightly higher activity against *h*MAO-A of compound **3** (IC_50_ = 1.38 µM) with respect to **4** (IC_50_ = 2.59 µM).

Finally, with the aim of comparing the new series of benzothiophene ligands with the parent benzofuran analogues from the point of view of their potential interactions with the targets, and thus evaluating how the isosteric replacement could have influenced the binding modes of the two compound series, our docking–MD protocol was finally applied to compound **BF13** (Table S2), which represents the benzofuran analogue of compound **4**. Interestingly, our computational analysis suggested a different orientation of the benzofuran core of **BF13** (Figure S3A) compared to that of the benzothiophene scaffold of **4** (Figure 8). Consistently with the lower aromaticity level and hydrophobicity of the furan ring and with the higher electronegativity and polarity of the oxygen, the benzofuran core of **BF13** was predicted to be oriented with the oxygen facing the solvent-accessible region of the *h*MAO-B catalytic pocket, which allowed the formation of a network of water-mediated H-bonds with the backbone oxygen atoms of P102 and I119 (Figure S3A). Notably, the formation of such H-bond network did not hamper the establishment of the key interactions also observed for compound **4**, i.e., the H-bonds with the OH groups of Y326 and Y398, the lipophilic interactions formed with W119, F168, L171, I199, I316 and Y326 by the bicyclic core, and the hydrophobic contacts with L171, C172, I198, and Y398 shown by the *p*-methoxyl group of the ligand. These results can explain the higher *h*MAO-B inhibitory activity of **BF13** (IC_50_ = 0.0085 µM) compared to compound **4** (IC_50_ = 0.057 µM). In contrast, the binding mode predicted for **BF13** with *h*MAO-A was found to be almost identical to that of compound **4**, as the same orientation and conformation of the ligand within the protein catalytic site, as well as the same pattern of hydrophobic interactions, were observed. The only difference may be constituted by the interaction of the two ligands with the cofactor. The more polar, less aromatic and hydrophobic benzofuran ring may not be able to form equally strong hydrophobic interactions with the protein cofactor, which might explain the slightly lower activity of **BF13** against *h*MAO-A (IC_50_ = 5.58 µM) compared to compound **4** (IC_50_ = 2.59 µM).

## 4. Conclusions

Neurodegenerative disorders exhibit complex etiopathology and limited therapeutic options, largely restricted to symptomatic treatments. Owing to its role in dopaminergic metabolism, *h*MAO-B is a validated target in Parkinson’s disease and is increasingly recognized for its involvement in other neurodegenerative conditions. In pursuit of novel potent and selective *h*MAO-B inhibitors, in this work we reported the design, synthesis, binding mode prediction, and preliminary in vitro neuroprotective evaluation of a library of 2-aroylbenzothiophenes within a PD cell-based model. The design of the 2-aroylbenzothiophenes was based (i) on the oxygen-to-sulfur isosteric replacement of the 2-aroylbenzofuran scaffold and (ii) on the removal of the 3-hydroxyl group from the benzothiophene-3-ol core. Potency and selectivity against *h*MAO-A and *h*MAO-B were revealed to be strongly influenced by the different substituents inserted into the aryl moiety. Substitutions of the parent compound **1** generally improved potency against isoform B, although the degree of improvement varied depending on the specific substituent used. For example, *para*-methoxy **4**, *para*-cyano **13** and *meta*/*para*-nitro derivatives (**11** and **12**, respectively) showed inhibition values in the nanomolar range (IC_50_ = 21–68 nM). As for *h*MAO A, compounds bearing *para*-nitro and *para*-cyano substitutions (**12** and **13**, respectively) demonstrated the highest inhibitory activity. Molecular modelling studies were then conducted to predict the binding mode of this novel class. Specifically, the *para*-methoxy-substituted compound **4** and its parent unsubstituted compound **1** were selected to elucidate the differing potency observed between these two analogues toward isoform B. The results indicated that both compounds exhibited comparable orientations within the binding pocket. However, the extensive network of interactions established by compound **4**, which are not present in the unsubstituted analogue **1**, resulted in increased potency of compound **4** against *h*MAO B. Moreover, we compared the new benzothiophene series with the parent benzofuran analogues in terms of their potential target interactions in order to evaluate how the isosteric replacement could have influenced the binding modes of the bioisosteres. Interestingly, comparison between compound **4** and its benzofuran counterpart **BF13** revealed that oxygen-to-sulfur isosteric replacement significantly affects ligand orientation and interaction patterns with *h*MAO-B, while preserving key stabilizing interactions. On the basis of their inhibitory profiles, compounds **4**, **11** and **12** were selected for the preliminary cellular investigation using two different cell lines. Cellular viability analysis using the MTS assay revealed that, except for compound **4**, the compounds are well tolerated up to 100 μM after 24 h of incubation, with profiles comparable to that of (*R*)-(−)-Deprenyl. The high toxicity shown for compound **4** may be partially ascribed to its effect of dramatically increasing ROS production at 100 μM (demonstrated using H2DCFDA-loaded SH-SY5Y), suggesting that this compound may modulate specific cellular processes targeting mitochondrial function. Interestingly, compound **12** was revealed to have the opposite effect, reducing the baseline ROS production in this cell line, with no fluorescence increase detected regardless of the concentration used. In light of these promising results, the selected compounds and the reference drug (*R*)-(−)-Deprenyl were studied for their potential neuroprotective effects on 6-OHDA-treated SH-SY5Y cells, in different conditions. After 24 h of co-treatment with 500 µM 6-OHDA, none of the tested compounds fully prevented 6-OHDA-induced toxicity, although compound **12** showed modest protective effects. Similarly, (*R*)-(−)-Deprenyl failed to provide neuroprotection under these conditions. To further explore their potential neuroprotective effects, compounds were tested at a sub-toxic 6-OHDA concentration (250 µM) for 24, 48, and 72 h. Under these conditions and after 72 h, although not statistically significant, derivatives **11** and **12** demonstrated a neuroprotective profile comparable to that of clinically used (*R*)-(−)-Deprenyl. Nevertheless, these preliminary results provide a foundation for a further investigation into this novel class of MAO inhibitors, with the objective of more comprehensively assessing their therapeutic potential in the context of neurodegenerative disorders.

## Figures and Tables

**Table 1:** Inhibitory activity of compounds **1**–**15** against *h*MAO-A and *h*MAO-B. ^a^ Values are the mean ± SD of triplicate determinations. ^b^ The selectivity index for the MAO-B isoform, given as the ratio (IC_50_ *h*MAO-A)/(IC_50_ *h*MAO-B). ^c^ Values taken from reference [64]. ^d^ Values from reference [36].

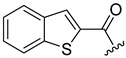		IC_50_ ± SD (µM) ^a^		SI ^b^
Name	R	*h*MAO-A	*h*MAO-B	
**1**	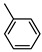	2.46 ± 0.725	2.71 ± 0.204	0.9
**2**	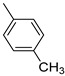	2.13 ± 0.106	0.177 ± 0.054	12.0
**3**	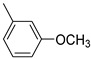	1.38 ± 0.063	0.325 ± 0.051	4.2
**4**	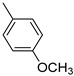	2.59 ± 0.283	0.057 ± 0.024	45.4
**5**	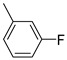	1.71 ± 0.501	0.431 ± 0.029	3.97
**6**	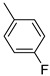	5.76 ± 1.12	0.483 ± 0.047	11.9
**7**	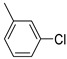	9.54 ± 1.30	0.187 ± 0.010	51.0
**8**	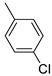	0.933 ± 0.151	0.266 ± 0.111	3.5
**9**	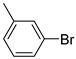	8.02 ± 0.479	0.131 ± 0.028	61.2
**10**	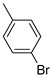	0.948 ± 0.365	0.378 ± 0.035	2.5
**11**	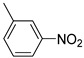	0.918 ± 0.210	0.021 ± 0.008	43.7
**12**	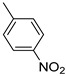	0.062 ± 0.005	0.045 ± 0.006	1.4
**13**	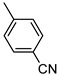	0.064 ± 0.009	0.068 ± 0.018	0.9
**14**	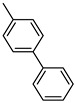	3.64 ± 0.199	1.54 ± 0.966	2.4
**15**	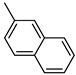	15.9 ± 0.269	0.243 ± 0.022	65.4
**Toloxatone**	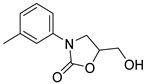	3.92 ± 0.15 ^c^	/	
**Lazabemide**	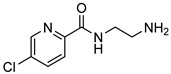	/	0.091 ± 0.15 ^c^	
**(** ***R*** **)-(−)-** **Deprenyl**	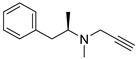	/	0.041 ± 0.006 ^d^	

## Data Availability

The original contributions presented in this study are included in the article/Supplementary Materials. Further inquiries can be directed to the corresponding authors.

## Supplementary Materials

The following supporting information can be downloaded at https://www.mdpi.com/article/10.3390/antiox15030346/s1. Chemistry. Table S1. Inhibitory activity (IC50) and selectivity index (SI) of benzothiophen-3-oles **PM1-PM20** towards hMAO-A and hMAO-B; Table S2. Inhibitory activity (IC50) and selectivity index (SI) of benzofurans **1**–**21** towards hMAO-A and hMAO-B; Figure S1. Cell viability using MTS assay and normalized to control cells treated with DMSO (0.2% as final concentration); Figure S2. Predicted binding modes of compound 3 with *h*MAO-B (A) and *h*MAO-A (B); Figure S3. Predicted binding modes of compound BF13 with *h*MAO-B (A) and *h*MAO-A (B). NMR and mass analyses of newly synthesized compounds.
