# Supplementary material for: Novel Insights on Benzo[b]thiophene Analogues for MAO-B Inhibition and Neuroprotection: Design, Synthesis, Molecular Modelling Studies and Biological Activity
Source: Antioxidants (Basel). 2026 Mar 10;15(3):346. doi: 10.3390/antiox15030346 (PMC13023857; doi:10.3390/antiox15030346)
Supplement: Supplementary file 1 [file antioxidants-15-00346-s001.zip › Supporting Information - proof.pdf]

Novel insights on benzo[*b*]thiophene analogues for MAO-B inhibition and neuroprotection: design, synthesis, molecular modelling studies and biological activity

Francesca Arrighi [1,#], Emanuela Berrino [1,2,#], Paolo Guglielmi [1,*], Simone Carradori [3,*], Guya Diletta Marconi [4], Jacopo Pizzicannella [4], Simone Guarnieri [5], Tiziano Tuccinardi [6], Giulio Poli [6], Federico Pepi [1], Anna Troiani [1], Chiara Salvitti [1], Alessia Di Noi [1], Michele Coluccia [1], Giorgio Buttitta [1], Virginia Pontecorvi [1,7], Arianna Granese [1], Paola Chimenti [1], Daniela Secci [1], Anel Petzer [8,9], Jacobus Petrus Petzer [8,9], Francesca Diomede [4]

[1] Department of Drug Chemistry and Technologies, Sapienza University of Rome, P.le A. Moro 5, 00185 Rome, Italy; francesca.arrighi@uniroma1.it (F.A.); paolo.guglielmi@uniroma1.it (P.G); federico.pepi@uniroma1.it (F.P.); anna.troiani@uniroma1.it (A.T.); chiara.salvitti@uniroma1.it (C.S.); alessia.dinoi@uniroma1.it (A.D.N.); michele.coluccia@uniroma1.it (M.C.); giorgio.buttitta@uniroma1.it (G.B.); virginia.pontecorvi@uniroma1.it (V.P.); arianna.granese@uniroma1.it (A.G.); paola.chimenti@uniroma1.it (P.C.); daniela.secci@uniroma1.it (D.S.);

[2] Department of Life Science, Health, and Health Professions, Link Campus University, Via del Casale di San Pio V, 44 – 00165, Rome, Italy; e.berrino@unilink.it (E.B.)

[3] Department of Pharmacy, "G. d'Annunzio" University of Chieti-Pescara, Via dei Vestini 31, 66100 Chieti, Italy; simone.carradori@unich.it (S.C.)

[4] Department of Innovative Technologies in Medicine & Dentistry, "G. d'Annunzio" University of Chieti-Pescara, Chieti, Italy; guya.marconi@unich.it (G.D.M.); francesca.diomede@unich.it (F.D.)

[5] Department of Neuroscience, Imaging and Clinical Sciences, "G. d'Annunzio" University of Chieti-Pescara, Via Luigi Polacchi 11, 66100 Chieti, Italy; simone.guarnieri@unich.it (S.G.)

[6] Department of Pharmacy, Università di Pisa, via Bonanno 6, 56126 Pisa, Italy; tiziano.tuccinardi@unipi.it (T.T.); giulio.poli@unipi.it (G.P.)

[7] Department of Medico-Surgical Sciences and Biotechnologies, Sapienza University of Rome, Corso della Repubblica 79, 04100, Latina, Italy; virginia.pontecorvi@uniroma1.it (V.P.)

[8] Centre of Excellence for Pharmaceutical Sciences, North-West University, Potchefstroom, 2520, South Africa; Anel.Petzer@nwu.ac.za (A.P.); jacques.petzer@nwu.ac.za (J.P.P.)

[9] Pharmaceutical Chemistry, School of Pharmacy, North-West University, Potchefstroom 2520, South Africa; Anel.Petzer@nwu.ac.za (A.P.); jacques.petzer@nwu.ac.za (J.P.P.)

*Correspondence: simone.carradori@unich.it (S.C.); paolo.guglielmi@uniroma1.it (P.G)

[#]These authors contributed equally to this work.

Summary

Chemistry.. 4

Table S1. Inhibitory activity (IC_{50}) and selectivity index (SI) of benzothiophen-3-oles **PM1**-**PM20** towards *h*MAO-A and *h*MAO-B [1].. 5

Table S2. Inhibitory activity (IC_{50}) and selectivity index (SI) of benzofurans **1**-**21** towards *h*MAO-A and *h*MAO-B [2]... 7

Figure S1. Cell viability assay using MTS assay and normalized to control cells treated with DMSO (0.2% as final concentration). A), B) and C) Histograms represent the cell viability of 6-OHDA-treated SH-SY5Y cells (250 µM) alone or co-incubated with (*R*)-(-)-Deprenyl, **11**, **12**, **BF20** at 100 µM and **4** at 12.5 µM, for 24 h,48 h and 72 h. 9

Figure S2. Predicted binding mode of compound **3** into *h*MAO-B (A) and *h*MAO-A (B). Compound 3 is shown in orange. For clarity, only the flavin group of the cofactor is shown in magenta. Ligand-protein H-bonds are shown as black dashed lines.. 10

Figure S3. Predicted binding mode of compound **BF13** into *h*MAO-B (A) and *h*MAO-A (B). Compound **BF13** is shown in blue. For clarity, only the flavin group of the cofactor is shown in magenta. Ligand-protein H-bonds are shown as black dashed lines. 11

^{1}H NMR of compound 1.. 12

^{13}C NMR of compound 1 13

^{1}H NMR of compound 2.. 14

^{13}C NMR of compound 2 15

^{1}H NMR of compound 3.. 16

^{13}C NMR of compound 3 17

^{1}H NMR of compound 4.. 18

^{13}C NMR of compound 4 19

^{1}H NMR of compound 5.. 20

^{13}C NMR of compound 5 21

^{13}C NMR of compound 6 23

^{1}H NMR of compound 7.. 24

^{13}C NMR of compound 7 25

^{1}H NMR of compound 8.. 26

^{13}C NMR of compound 8 27

^{1}H NMR of compound 9.. 28

^{13}C NMR of compound 9 29

^{1}H NMR of compound 10.. 30

^{13}C NMR of compound 10... 31

^{1}H NMR of compound 11.. 32

^{13}C NMR of compound 11 33
^{1}H NMR of compound 12 34
^{13}C NMR of compound 12 35
^{1}H NMR of compound 13 36
^{13}C NMR of compound 13 37
^{1}H NMR of compound 14 38
^{13}C NMR of compound 14 39
^{1}H NMR of compound 15 40
^{13}C NMR of compound 15 41
Mass spectrometric analysis 42
Mass Spectrum of Compound 1: 43
Mass Spectrum of Compound 2: 44
Mass Spectrum of Compound 3: 45
Mass Spectrum of Compound 4: 46
Mass Spectrum of Compound 5: 47
Mass Spectrum of Compound 6: 48
Mass Spectrum of Compound 7: 49
Mass Spectrum of Compound 8: 50
Mass Spectrum of Compound 9: 51
Mass Spectrum of Compound 10: 52
Mass Spectrum of Compound 11: 53
Mass Spectrum of Compound 12: 54
Mass Spectrum of Compound 13: 55
Mass Spectrum of Compound 14: 56
Mass Spectrum of Compound 15: 57
References 58

Chemistry

Synthesis of the 2-((2-(hydroxymethyl)phenyl)thio)-1-phenylethan-1-ones (compounds A1-A15)

To a stirred solution of mercaptobenzyl alcohol (1 eq.) in anhydrous DMF, potassium carbonate (2 eq.) was added, followed by α-bromoacetophenone (1.2 eq.). The reaction mixture was stirred at room temperature for 3 hours, after which complete consumption of the starting material was observed (as monitored by TLC 7:3, cyclohexane: ethyl acetate). Upon completion, the reaction was quenched by the addition of ice-cold water followed by acidification with hydrochloric acid. After extraction of the mixture with ethyl acetate (3×25mL), the organic layers were combined, washed with brine, and dried over anhydrous sodium sulphate. The drying agent was removed by *vacuum* filtration, and the organic phase was concentrated under reduced pressure. The resulting crude oil was used directly in the subsequent step without further purification.

Synthesis of the 2-((2-oxo-2-phenylethyl)thio)benzaldehydes (compounds B1-B15)

To a stirred solution of the alcohol obtained in the previous step (**A1**-**A15**) (1 eq.) in dry dichloromethane (DCM), Dess–Martin periodinane (DMP, 1.3 eq.) was dropwise added at 0 °C. The reaction mixture was then allowed to warm to room temperature and stirred for an additional 2 hours. Upon completion, as monitored by TLC (7:3, cyclohexane: ethyl acetate), the reaction was quenched with a saturated aqueous solution of sodium thiosulfate to decompose excess oxidant. The resulting mixture was extracted with DCM (3x25mL), and the combined organic layers were dried over anhydrous sodium sulphate and concentrated under reduced pressure. The crude solid was purified by chromatography employing as the eluent proper mixtures of cyclohexane and ethyl acetate.

Synthesis of the benzo[*b*]thiophen-2-yl(phenyl)methanones (compounds 1-15)

To a stirred solution of the aldehyde (**B1**-**B15**) (1 equiv.) in methanol, potassium hydroxide (2 equiv.) was added, and the reaction mixture was heated at 60 °C under a nitrogen atmosphere. After 2 hours, completion of the reaction was confirmed by thin-layer chromatography (TLC, 7:3 cyclohexane/ethyl acetate). The reaction mixture was then quenched with ice-cold water, and the pH was carefully adjusted to 3 using hydrochloric acid. The aqueous phase was extracted with ethyl acetate (3x25mL), and the combined organic extracts were washed with brine, dried over anhydrous sodium sulphate, and concentrated under reduced pressure to afford the crude product. The resulting solid was purified by column chromatography (9:1 cyclohexane: ethyl acetate), leading to the obtainment of a white solid. All the compounds (**1**-**15**) were further crystallized from petrol, affording transparent crystals.

Table S1. Inhibitory activity (IC_{50}) and selectivity index (SI) of benzothiophen-3-oles **PM1-PM20** towards *h*MAO-A and *h*MAO-B [1].

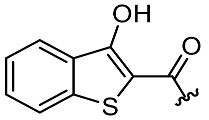		IC_{50} ± SD (µM)[a]		SI[b]
**Compound**	**Substituent**	**hMAO-A**	**hMAO-B**	
**PM1**	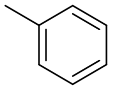	13.3 ± 0.29	7.39 ± 0.15	1.8
**PM2**	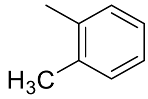	18.7 ± 2.99	23.4 ± 3.03	0.8
**PM3**	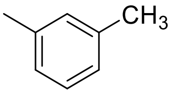	12.6 ± 1.58	1.81 ± 0.13	6.9
**PM4**	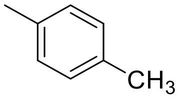	2.71 ± 0.14	0.47 ± 0.02	5.8
**PM5**	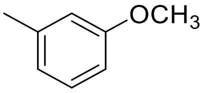	33.0 ± 0.88	0.78 ± 0.07	42.3
**PM6**	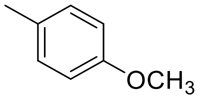	4.18 ± 0.77	0.28 ± 0.03	14.9
**PM7**	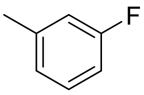	25.1 ± 2.17	1.44 ± 0.40	17.4
**PM8**	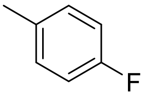	13.4 ± 0.92	2.28 ± 0.18	5.8
**PM9**	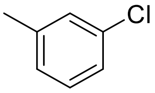	51.0 ± 1.72	0.55 ± 0.09	92.7
**PM10**	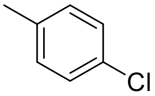	13.8 ± 0.51	0.89 ± 0.07	15.5
**PM11**	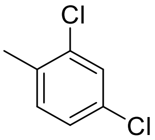	43.4 ± 2.81	37.3 ± 1.01	1.2
**PM12**	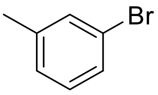	63.2 ± 3.48	0.35 ± 0.08	180
**PM13**	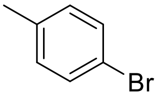	21.1 ± 1.98	0.88 ± 0.08	23.9

**PM14**	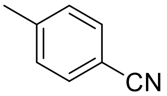	21.2 ± 3.27	4.51 ± 0.13	4.7
**PM15**	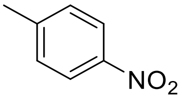	18.8 ± 1.53	2.75 ± 0.02	6.8
**PM16**	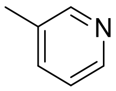	50.1 ± 2.56	49.7 ± 1.90	1.0
**PM17**	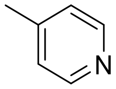	53.8 ± 2.90	56.9 ± 5.62	0.9
**PM18**	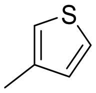	6.44 ± 0.68	7.56 ± 0.70	0.8
**PM19**	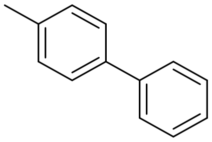	36.5 ± 1.07	5.59 ± 0.74	6.5
**PM20**	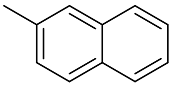	42.5 ± 1.88	1.08 ± 0.097	39.4

[a]Values are the mean ± SD of triplicate determinations. [b]Selectivity index for the *h*MAO-B isoform, given as the ratio: (IC_{50} *h*MAO-A)/(IC_{50} *h*MAO-B).

Table S2. Inhibitory activity (IC_{50}) and selectivity index (SI) of benzofurans **1-21** towards *h*MAO-A and *h*MAO-B [2].

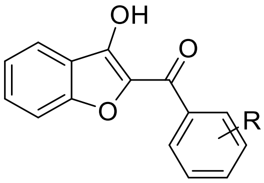

Compound	R	IC_{50} ± SD (μM)[a]		SI[b]
		*h*MAO-A	*h*MAO-B	
**BF1**	H	> 10 (0 %) [c]	> 10 (0.4 %) [c]	NA [d]
**BF2**	4-CH_3	> 10 (0 %) [c]	> 10 (40.7 %) [c]	NA [d]
**BF3**	3-OCH_3	> 10 (0 %) [c]	> 10 (25.0 %) [c]	NA [d]
**BF4**	4-OCH_3	> 10 (0 %) [c]	> 10 (35.9%) [c]	NA [d]
**BF5**	4-F	> 10 (0 %) [c]	> 10 (0 %) [c]	NA [d]
**BF6**	3-Cl	> 10 (0 %) [c]	4.0835 ± 0.5234	> 2.4
**BF7**	4-Cl	> 10 (43.5%) [c]	> 10 (29.8%) [c]	NA [d]
**BF8**	3-Br	> 10 (0 %) [c]	> 10 (47.6%) [c]	NA [d]
**BF9**	4-Br	> 10 (0 %) [c]	> 10 (10.5 %) [c]	NA [d]
**BF10**	4-Ph	> 10 (0 %) [c]	> 10 (32.7%) [c]	NA [d]
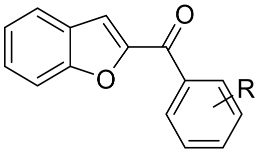				
**BF11**	H	9.0420 ± 1.7801	0.2117 ± 0.0223	43.4
**BF12**	4-CH_3	5.1064 ± 0.2941	0.0121 ± 0.0004	422.0
**BF13**	4-OCH_3	5.5852 ± 0.8187	0.0085 ± 0.0007	626.3
**BF14**	4-F	> 10 (45.8%) [c]	0.2296 ± 0.0127	> 43.7 [e]
**BF15**	3-Cl	3.4923 ± 0.2313	0.0188 ± 0.0003	186.6
**BF16**	4-Cl	7.2054 ± 0.2243	0.0268 ± 0.0016	268.8
**BF17**	2,4-diCl	7.2176 ± 0.9568	0.591 ± 0.0986	12.2
**BF18**	3-Br	6.4293 ± 0.4828	0.0135 ± 0.0009	494.6
**BF19**	4-Br	6.0640 ± 0.6323	0.0166 ± 0.0006	356.5
**BF20**	4-NO_2	> 10 (41.1%) [c]	0.0082 ± 0.0008	> 1221.0 [e]

**BF21**	4-Ph	> 10 (13.2%) [c]	0.1357 ± 0.0099	> 73.7 [e]
(*R*)-(-)-Deprenyl		> 10 (21.2%) [c]	0.0414 ± 0.0057	> 244.0 [e]
Clorgyline		0.0023 ± 0.0009	3.32 ± 0.4235	0.00069

[a] Values are the mean ± SD of triplicate determinations. [b] Selectivity index for the *h*MAO-B isoform, given as the ratio: (IC_{50} *h*MAO-A)/(IC_{50} *h*MAO-B). [c] The values in brackets indicate the percentage of inhibition observed during the initial screening at a fixed concentration of 10 μM. [d] Not assessable. [e] Values obtained assuming that the corresponding IC_{50} value against *h*MAO-A is the highest concentration tested (10 μM).

Figure S1. Cell viability assay using MTS assay and normalized to control cells treated with DMSO (0.2% as final concentration). A), B) and C) Histograms represent the cell viability of 6-OHDA-treated SH-SY5Y cells (250 μM) alone or co-incubated with (*R*)-(-)-Deprenyl, **11**, **12**, **BF20** at 100 μM and **4** at 12.5 μM, for 24 h,48 h and 72 h.

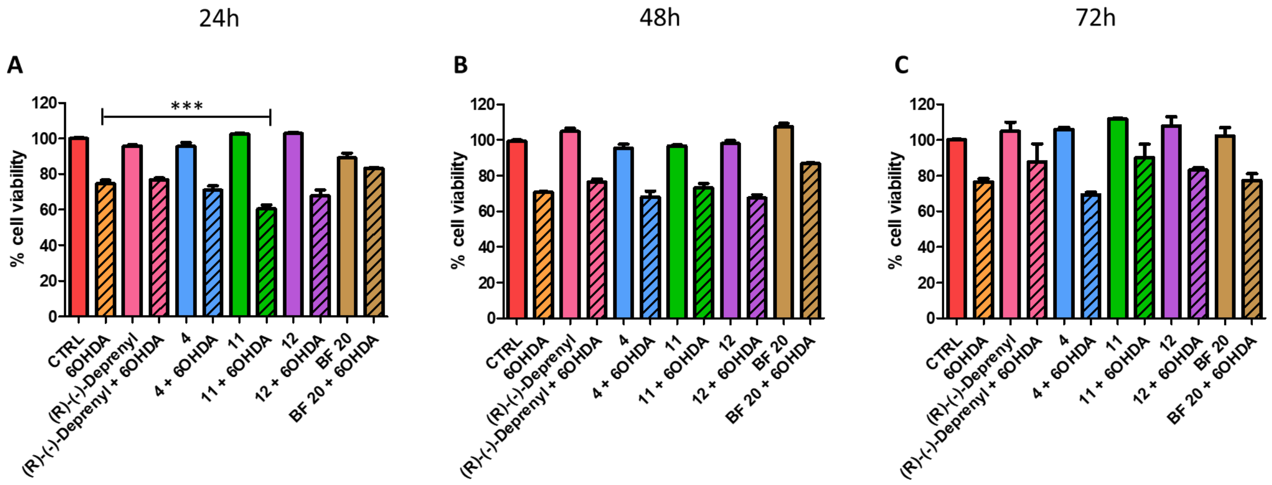

Figure S2. Predicted binding mode of compound **3** into *h*MAO-B (A) and *h*MAO-A (B). Compound 3 is shown in orange. For clarity, only the flavin group of the cofactor is shown in magenta. Ligand-protein H-bonds are shown as black dashed lines.

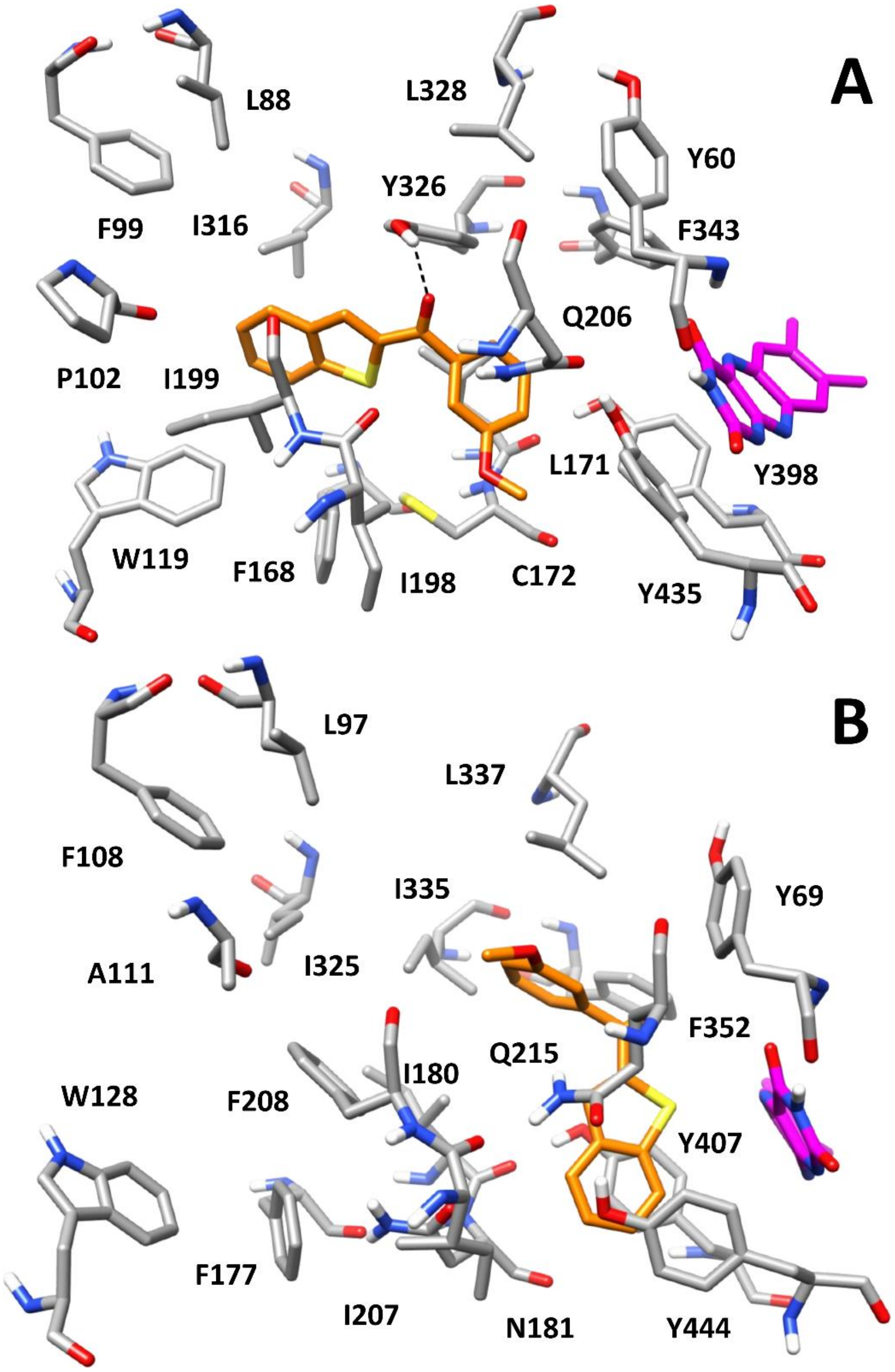

Figure S3. Predicted binding mode of compound **BF13** into *h*MAO-B (A) and *h*MAO-A (B). Compound **BF13** is shown in blue. For clarity, only the flavin group of the cofactor is shown in magenta. Ligand-protein H-bonds are shown as black dashed lines.

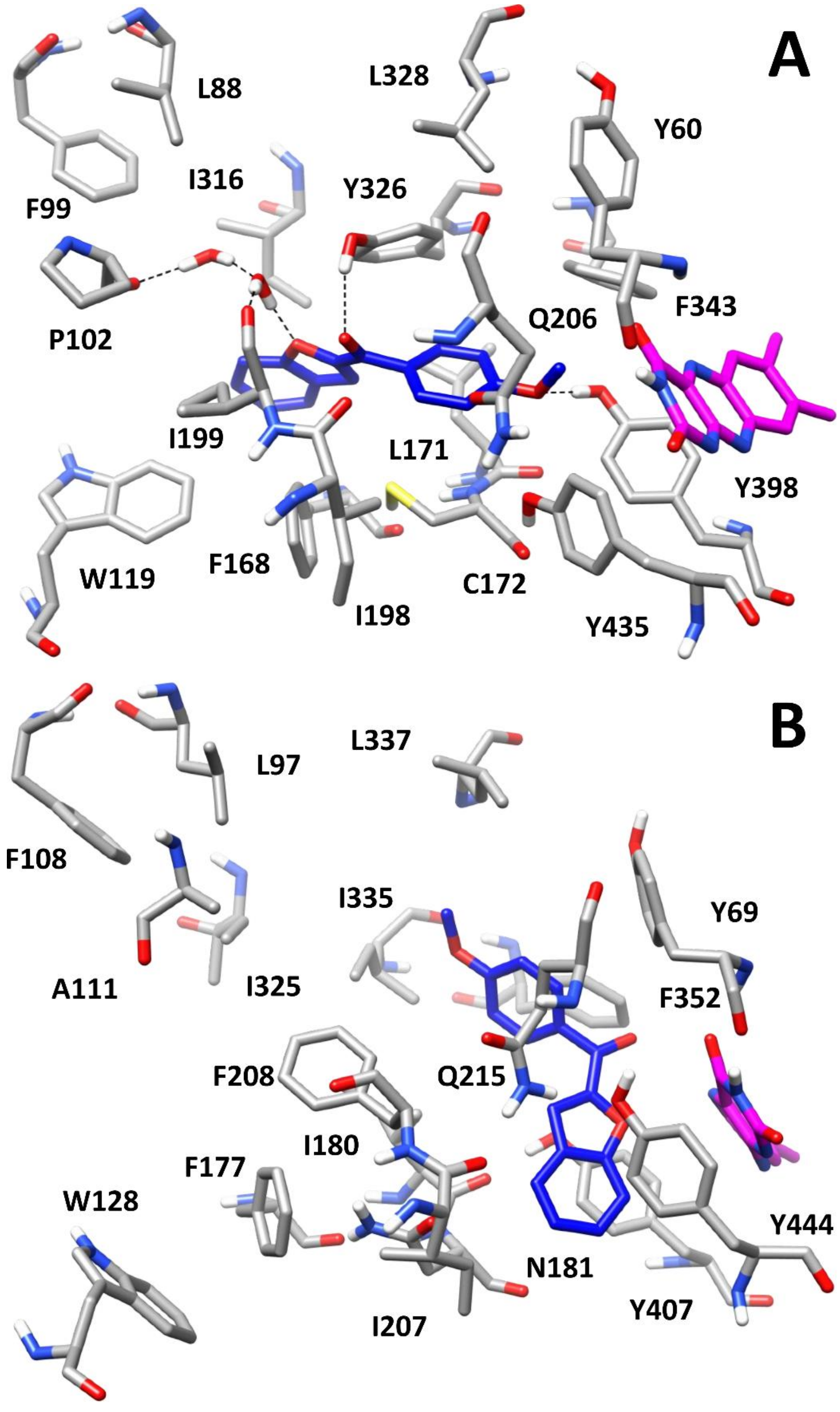

^{1}H NMR of compound 1

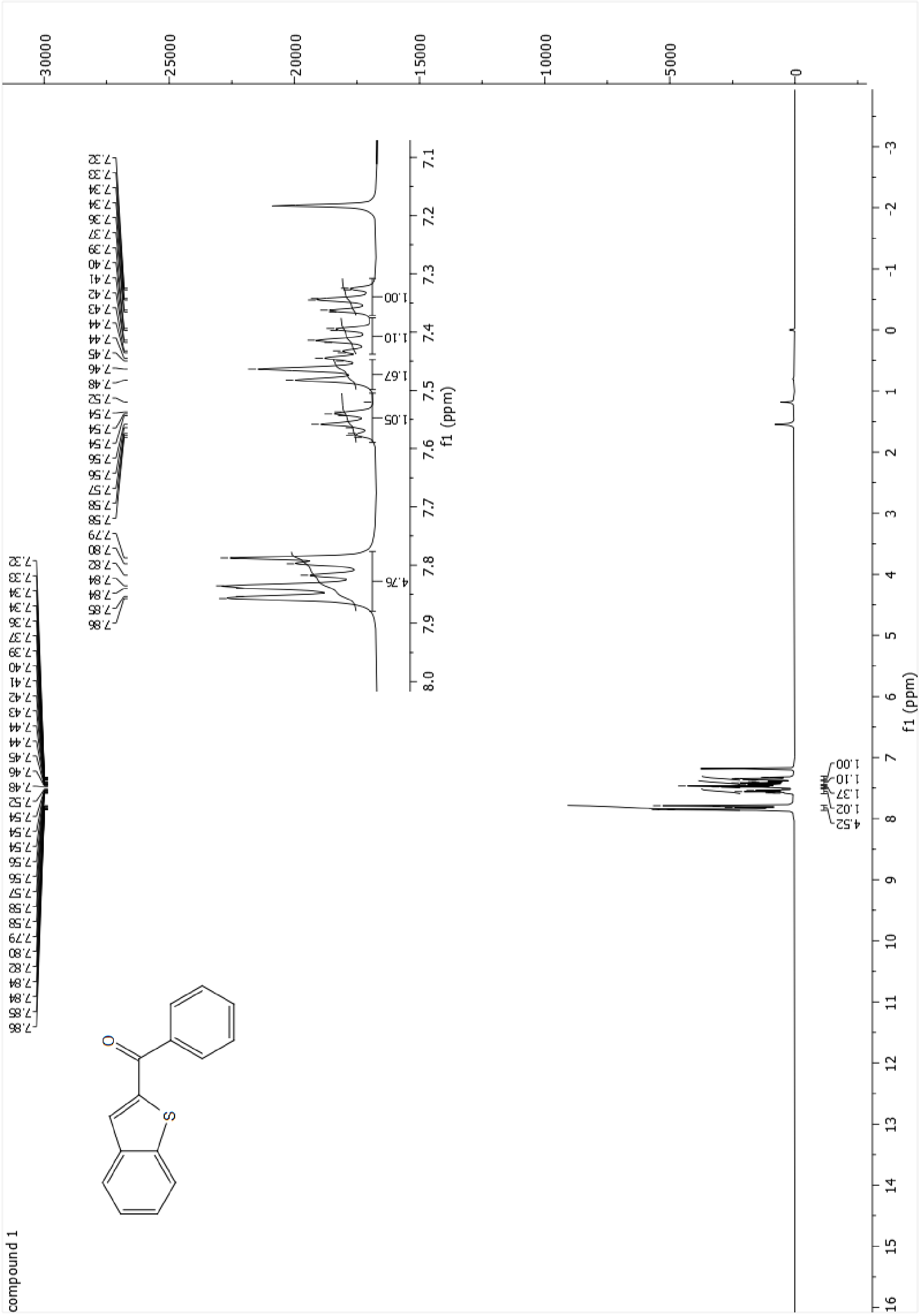

^{13}C NMR of compound 1

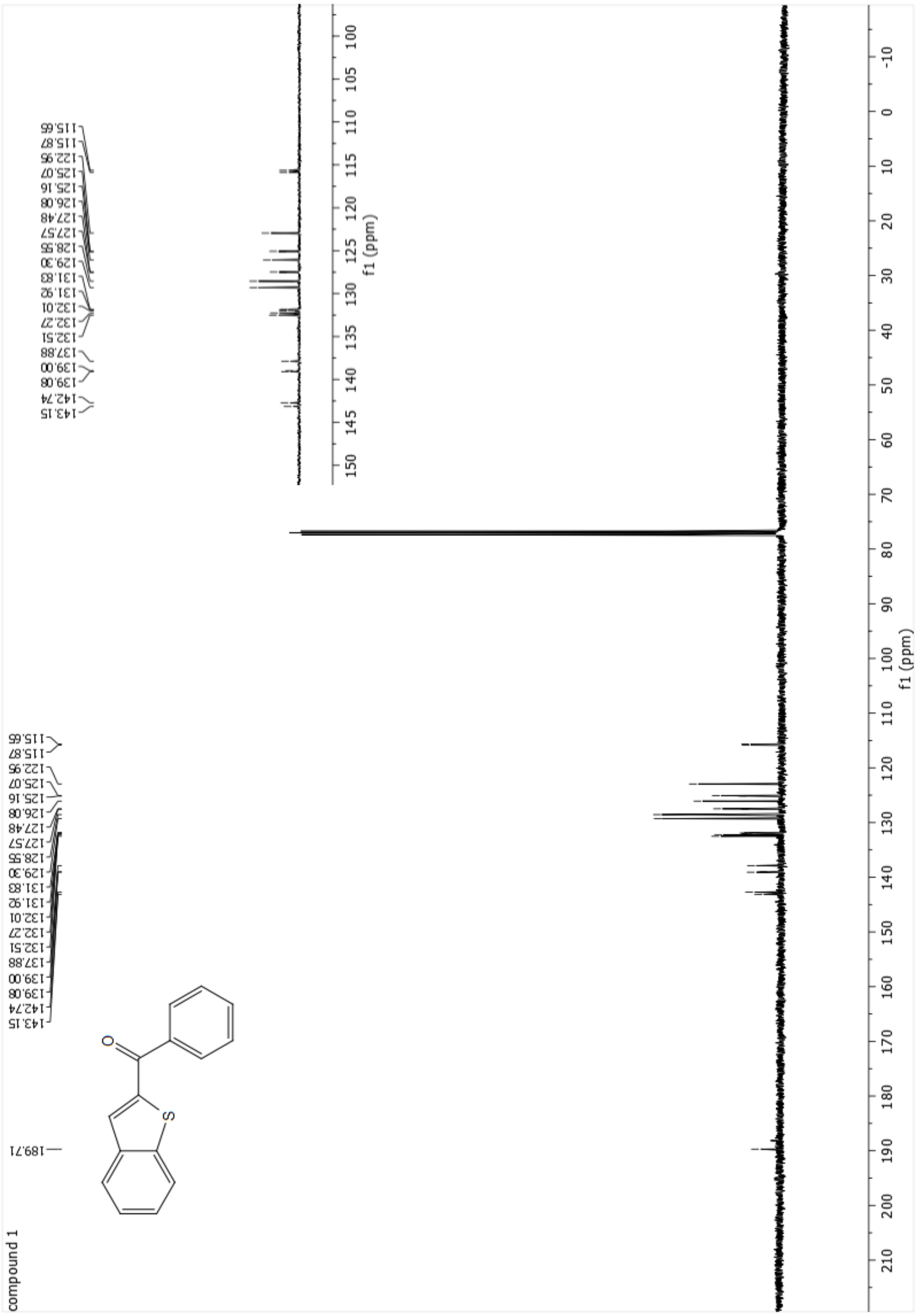

^{1}H NMR of compound 2

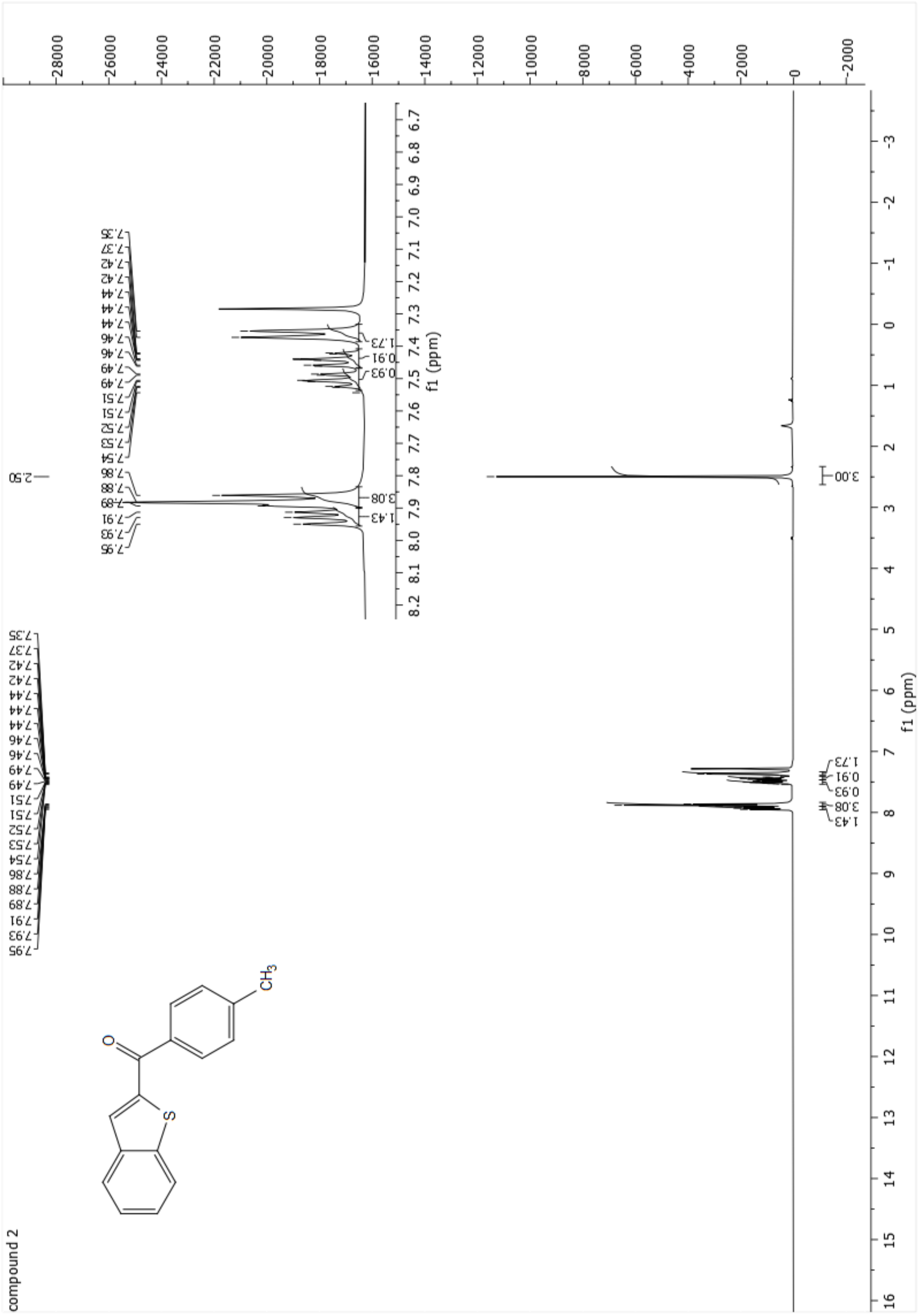

^{13}C NMR of compound 2

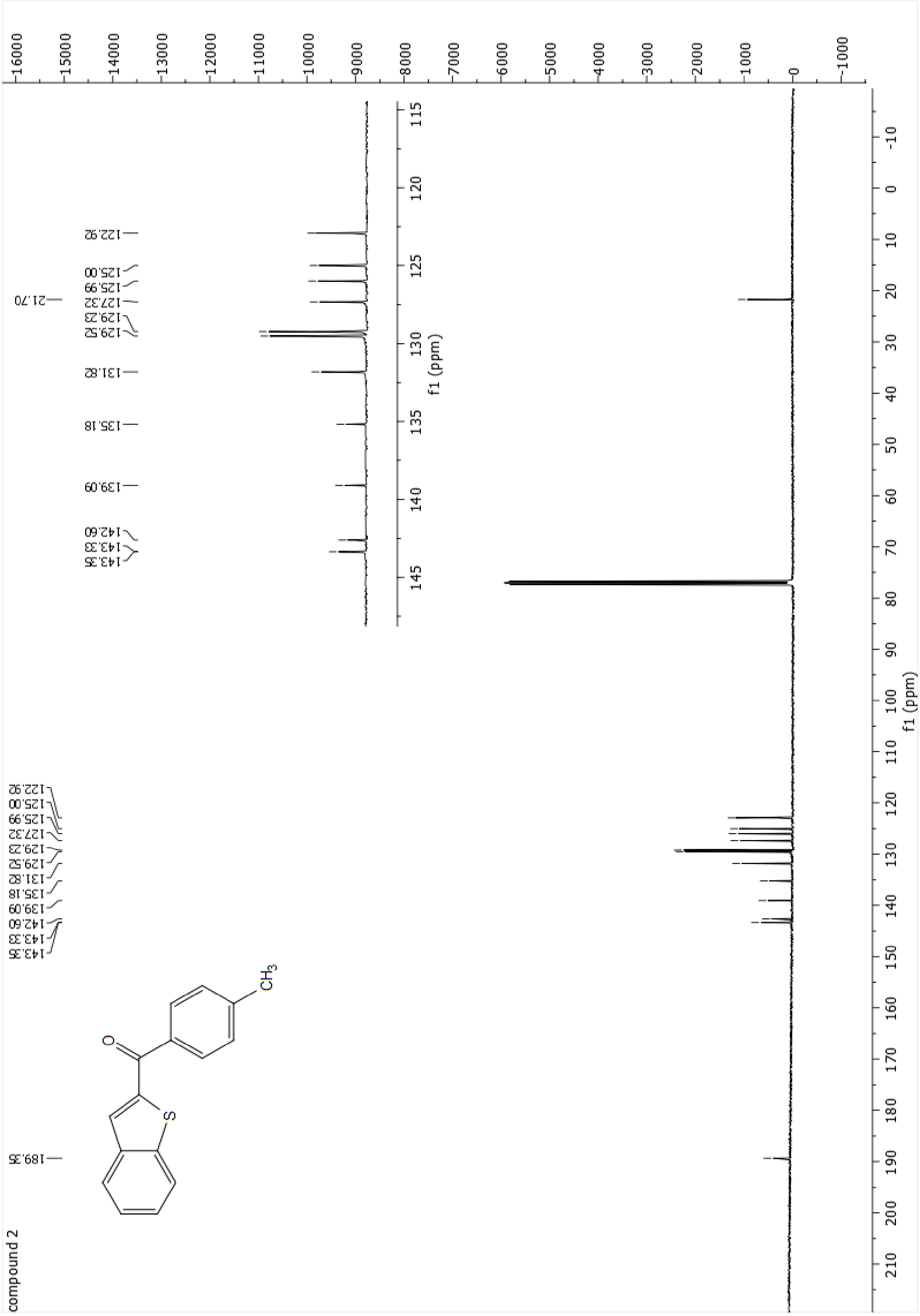

^{1}H NMR of compound 3

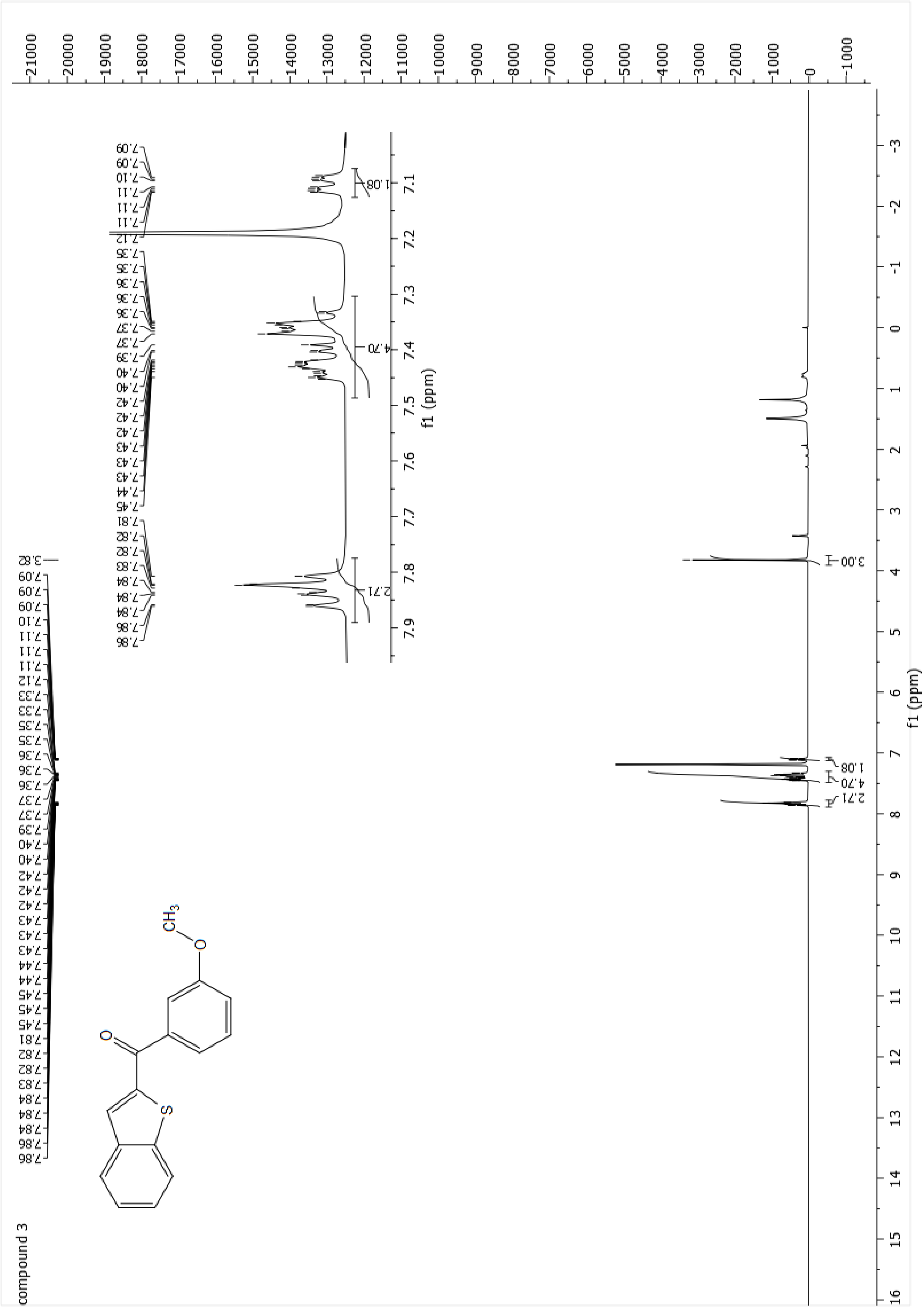

^{13}C NMR of compound 3

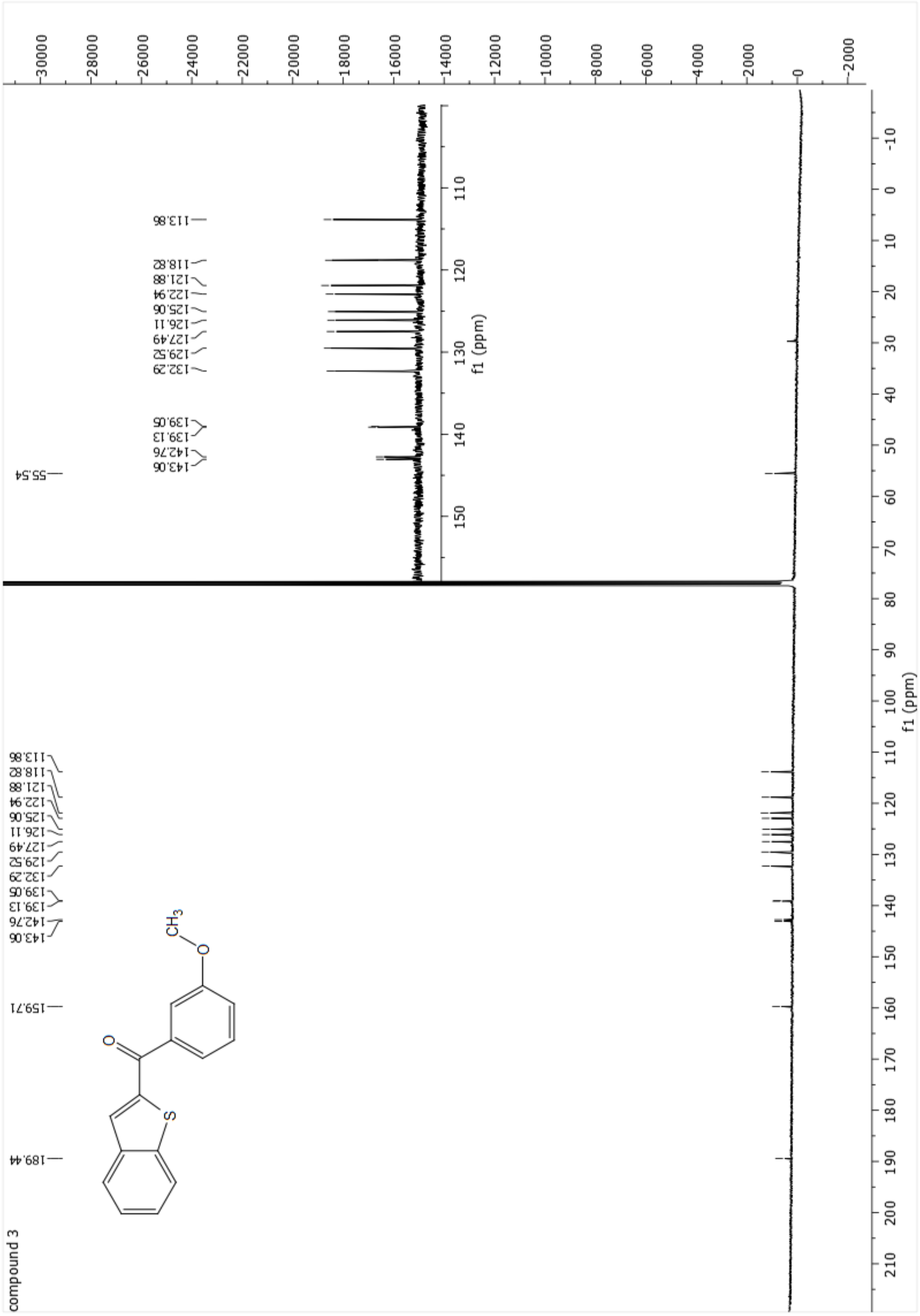

^{1}H NMR of compound 4

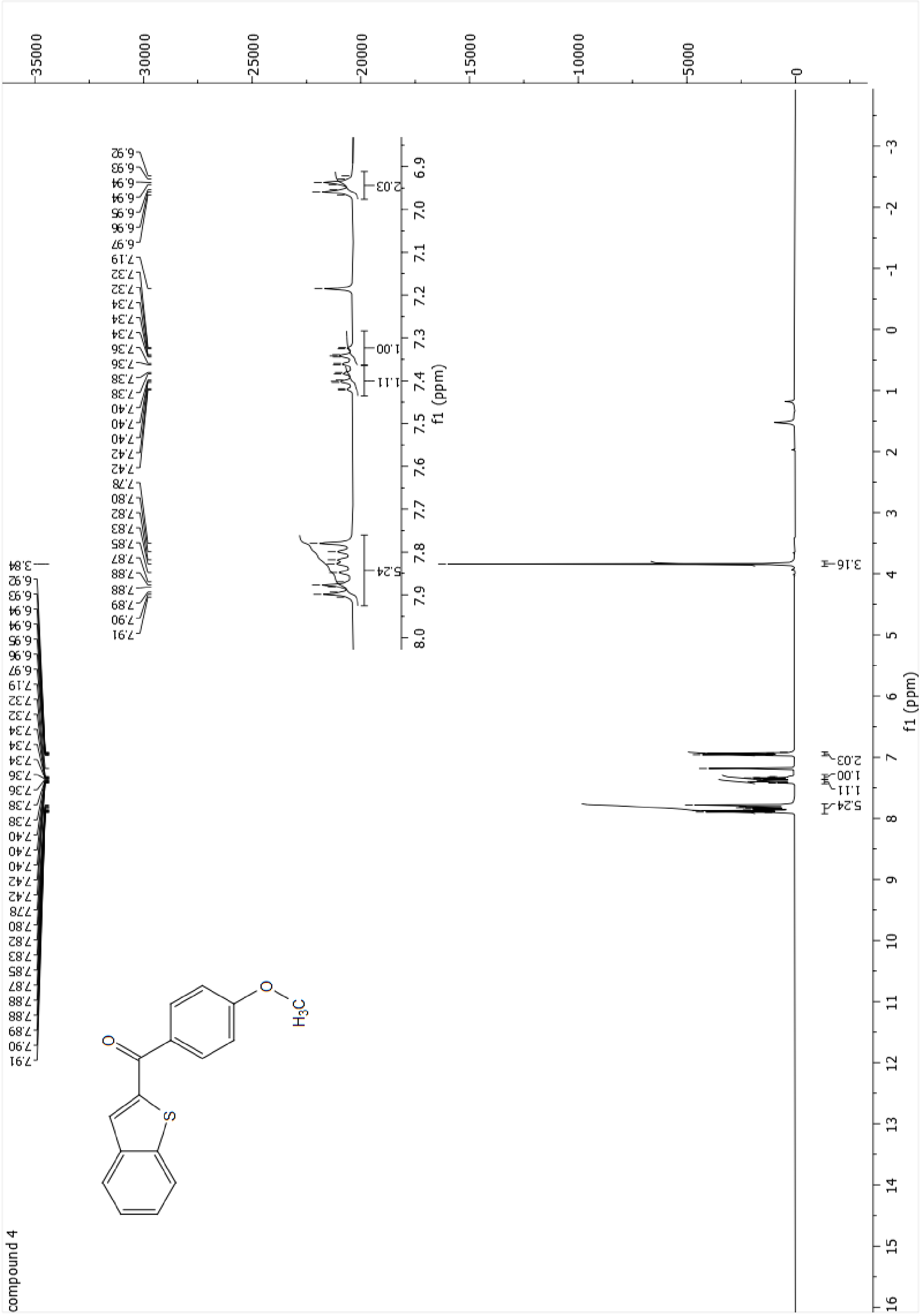

^{13}C NMR of compound 4

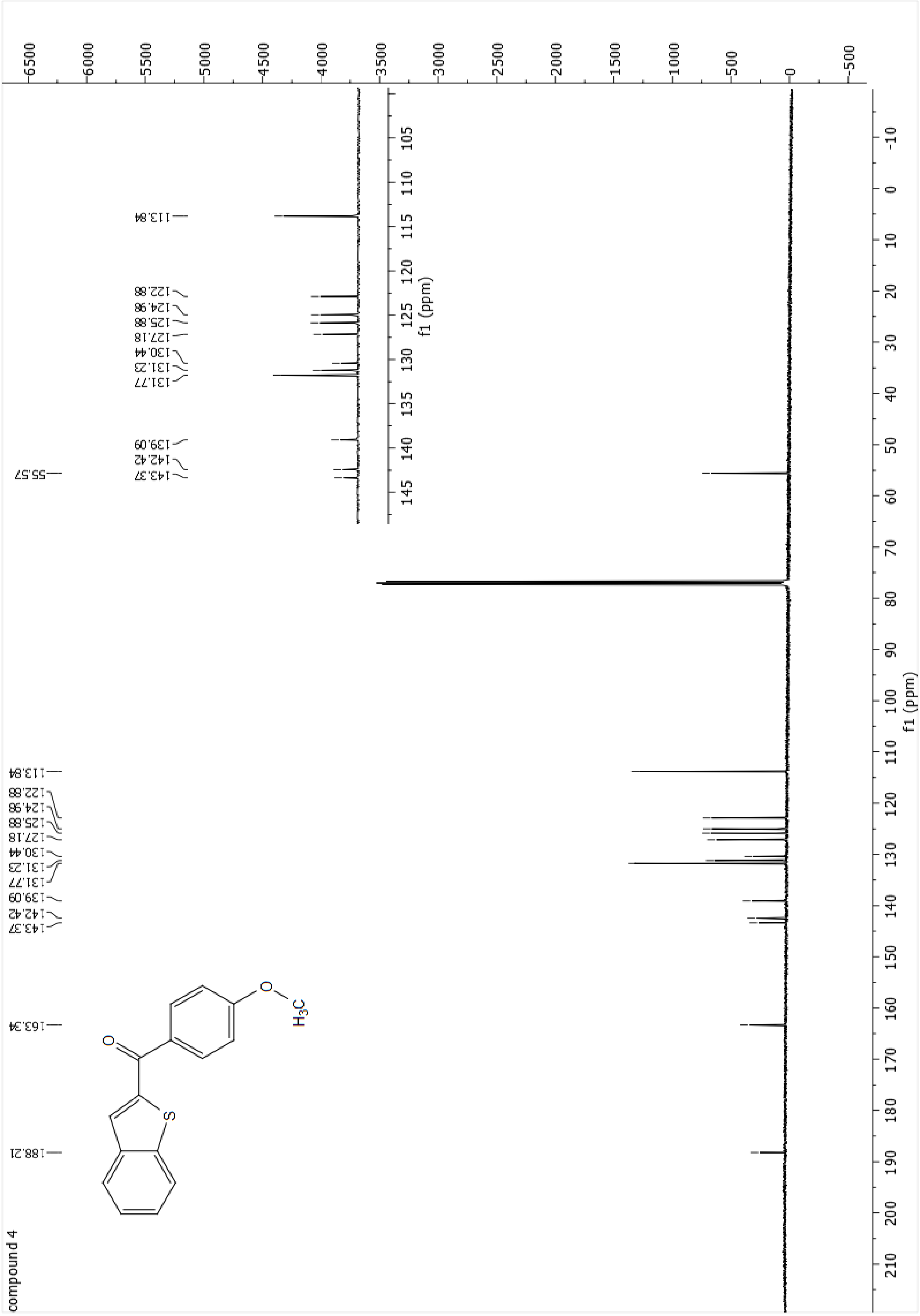

^{1}H NMR of compound 5

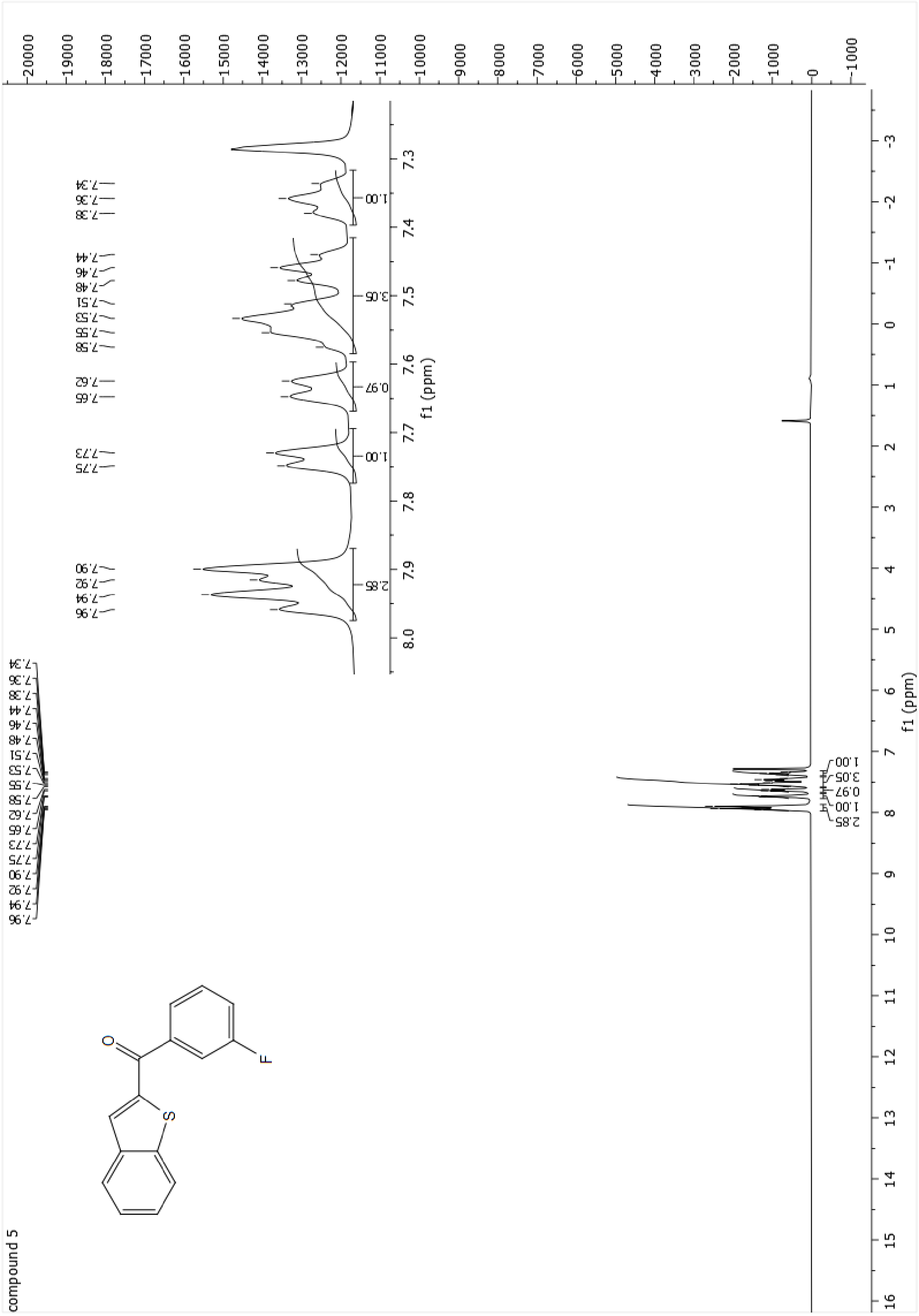

^{13}C NMR of compound 5

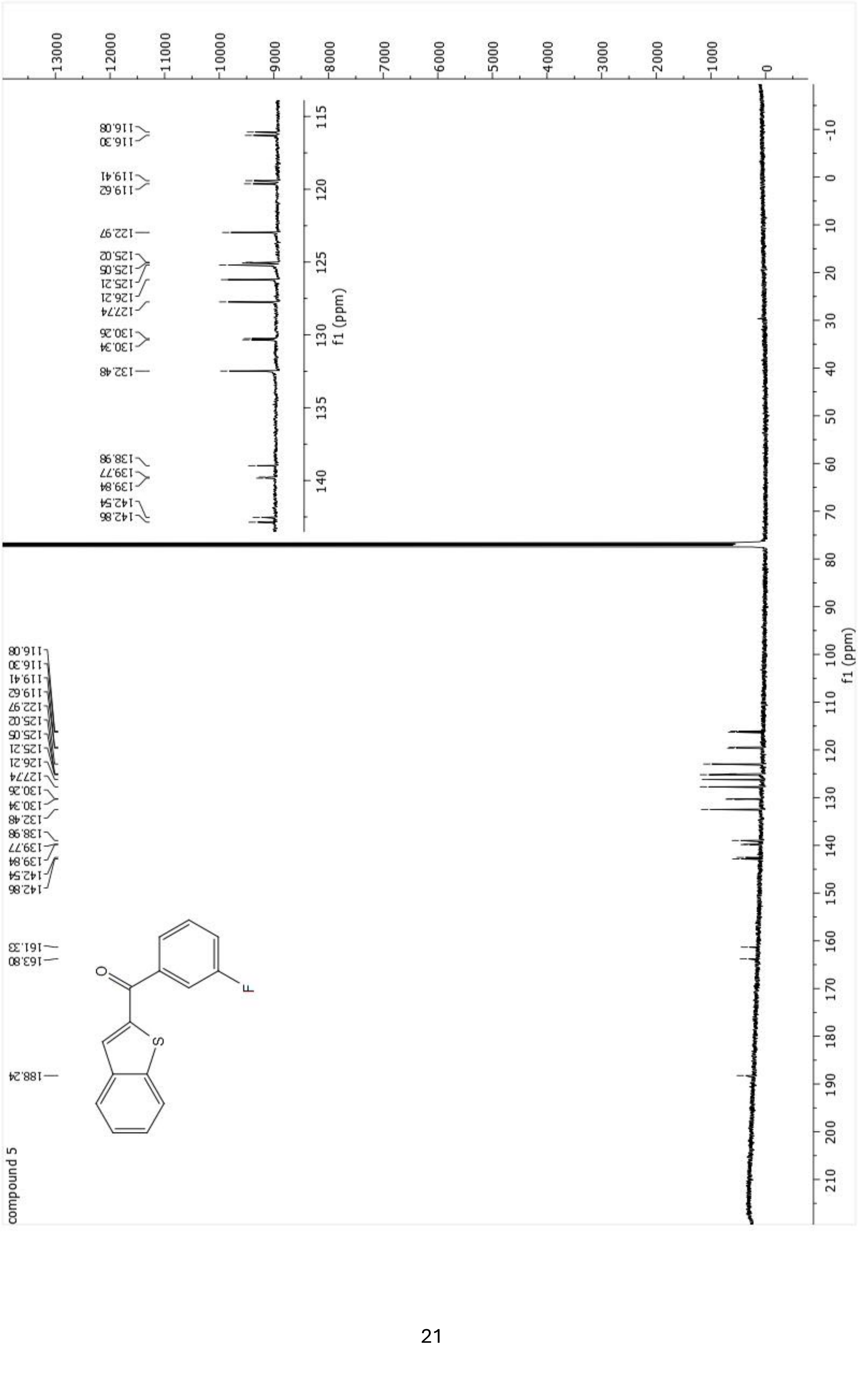

^{1}H NMR of compound 6

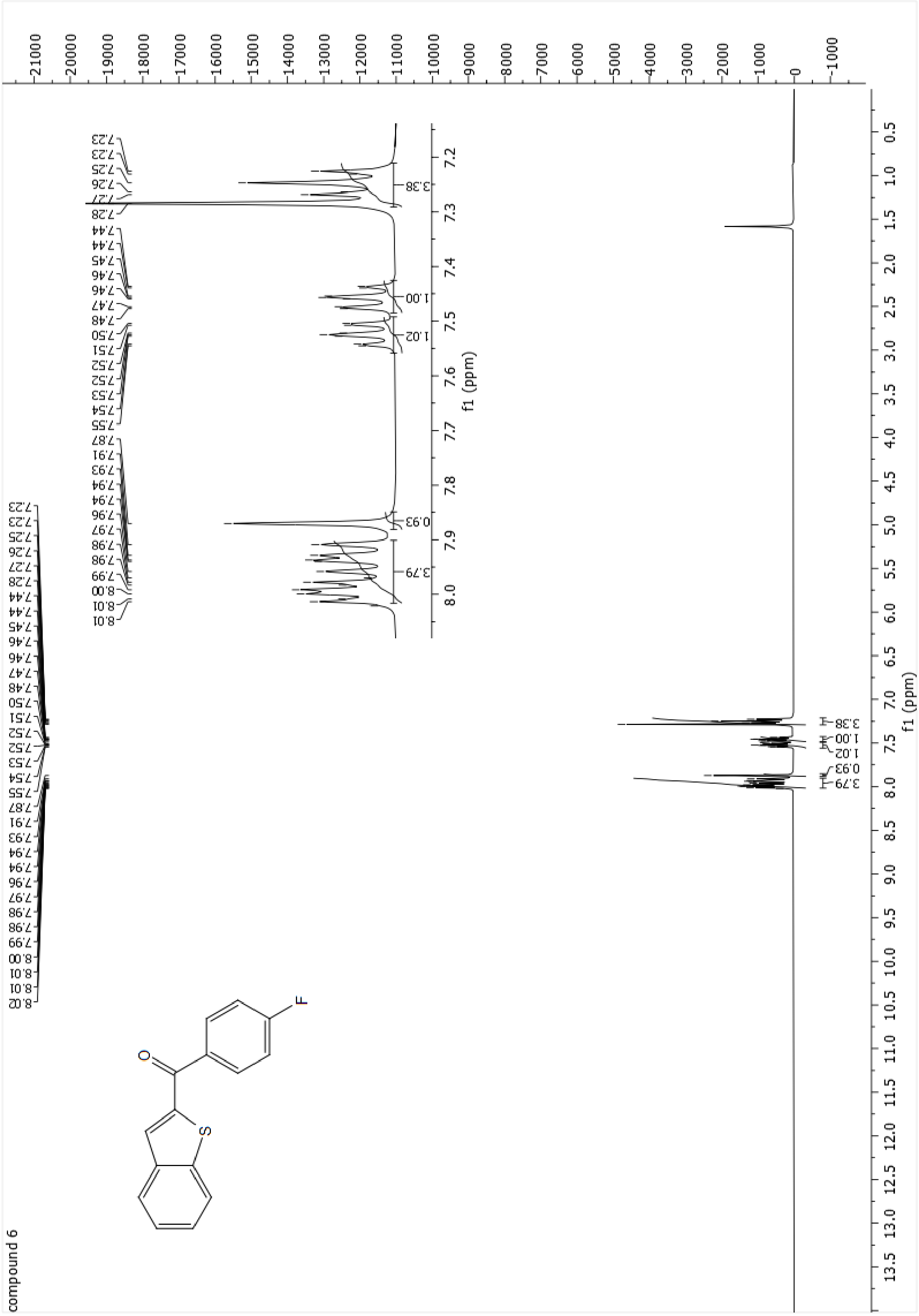

[13] C NMR of compound 6

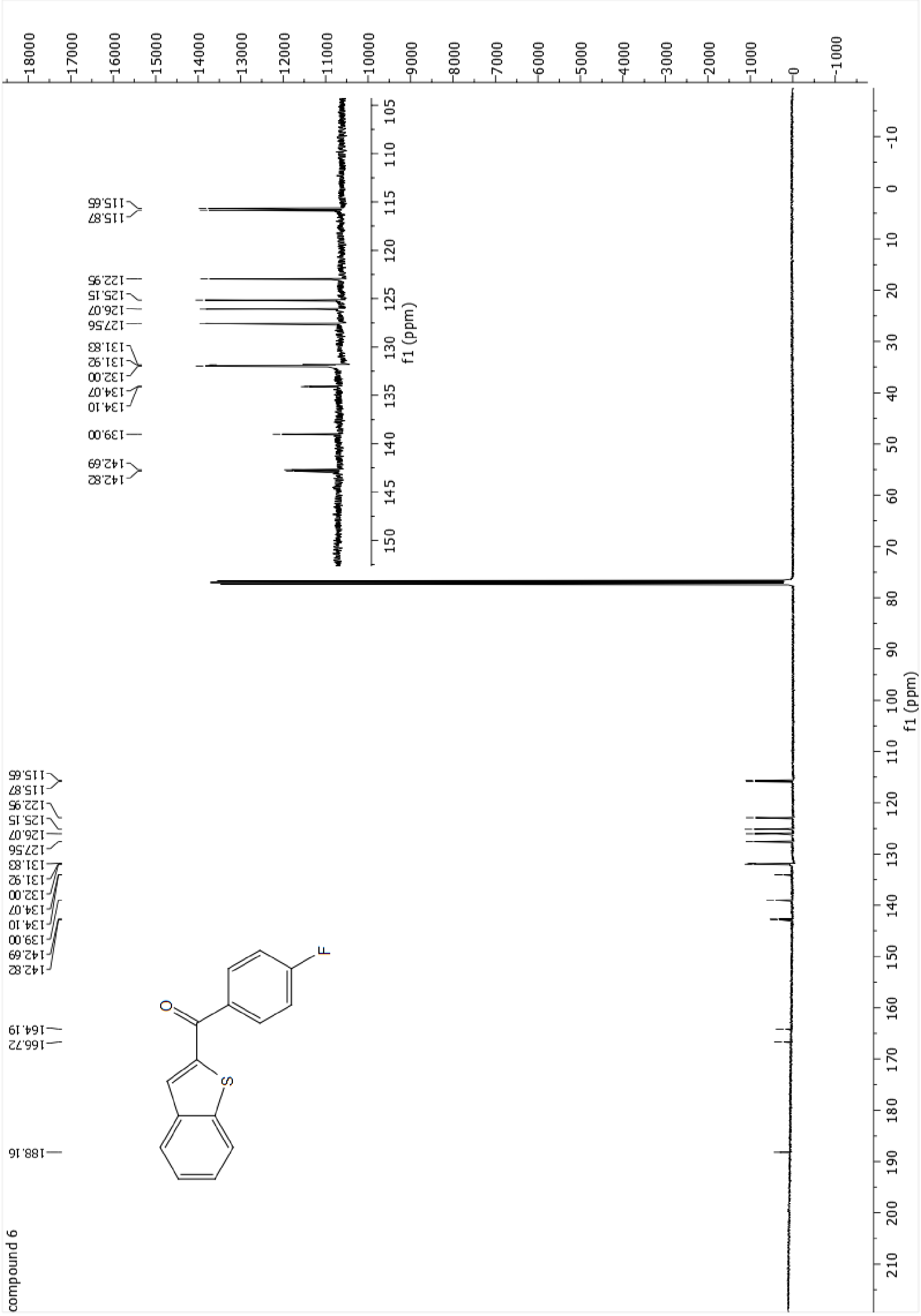

^{1}H NMR of compound 7

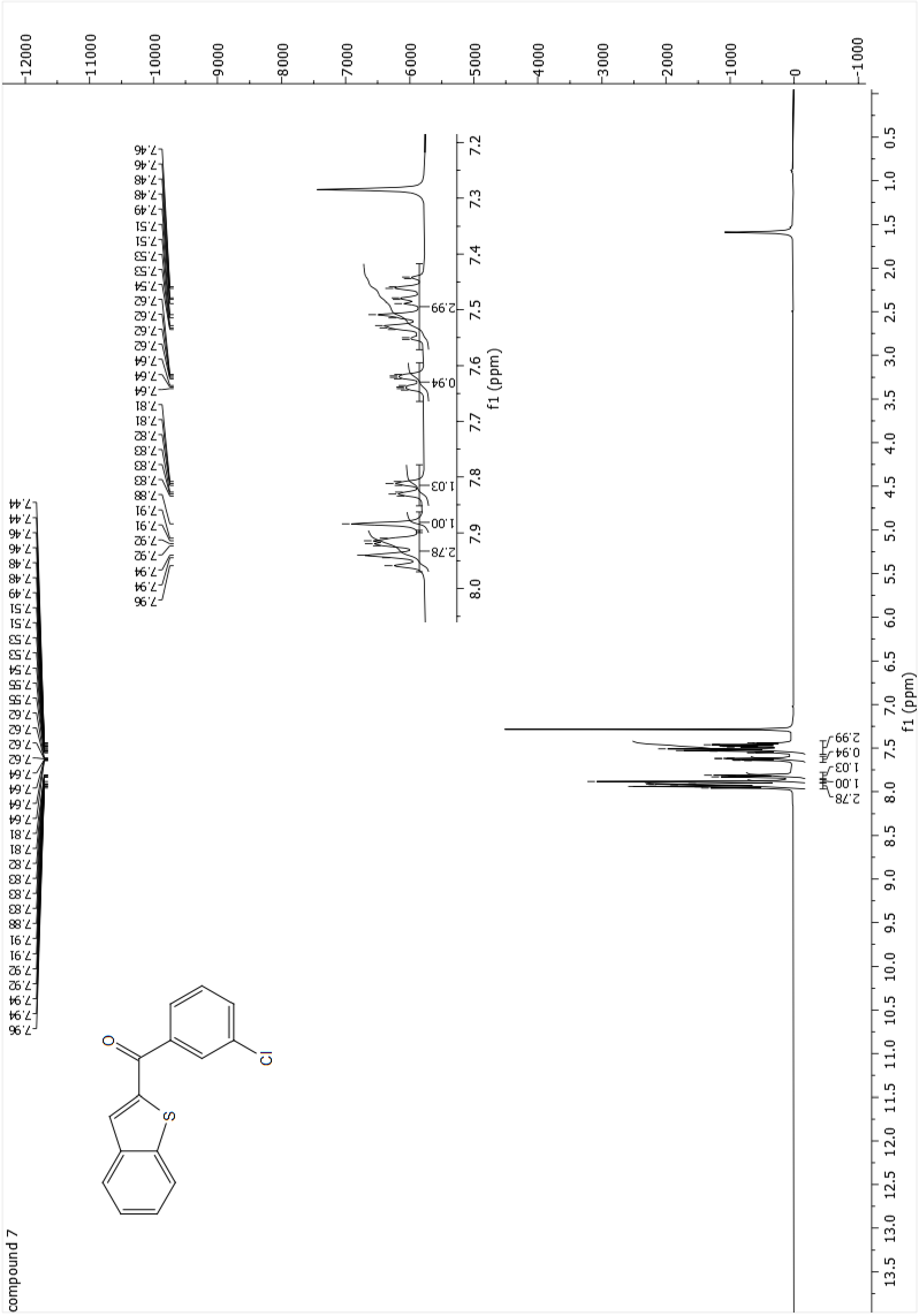

^{13}C NMR of compound 7

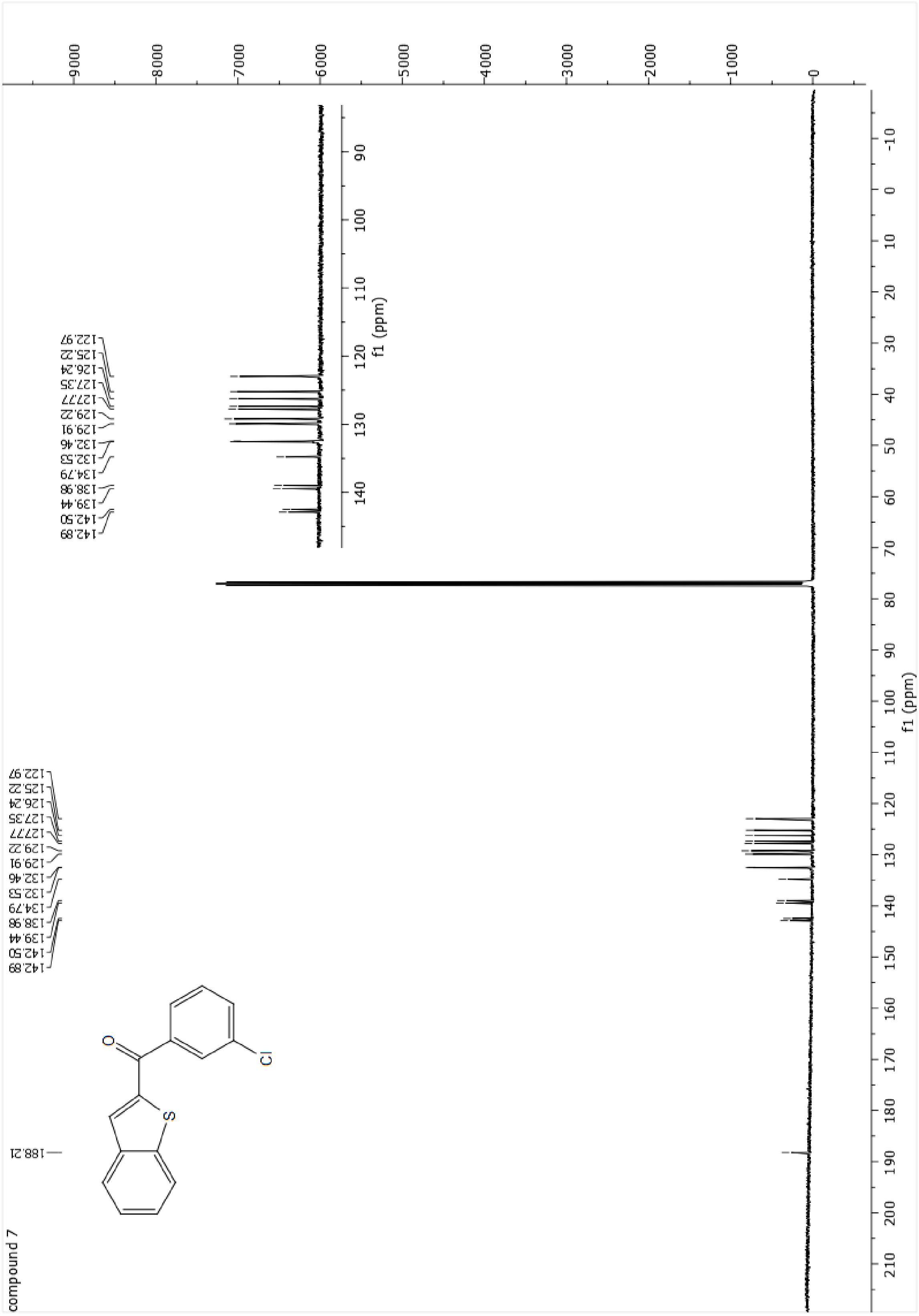

^{1}H NMR of compound 8

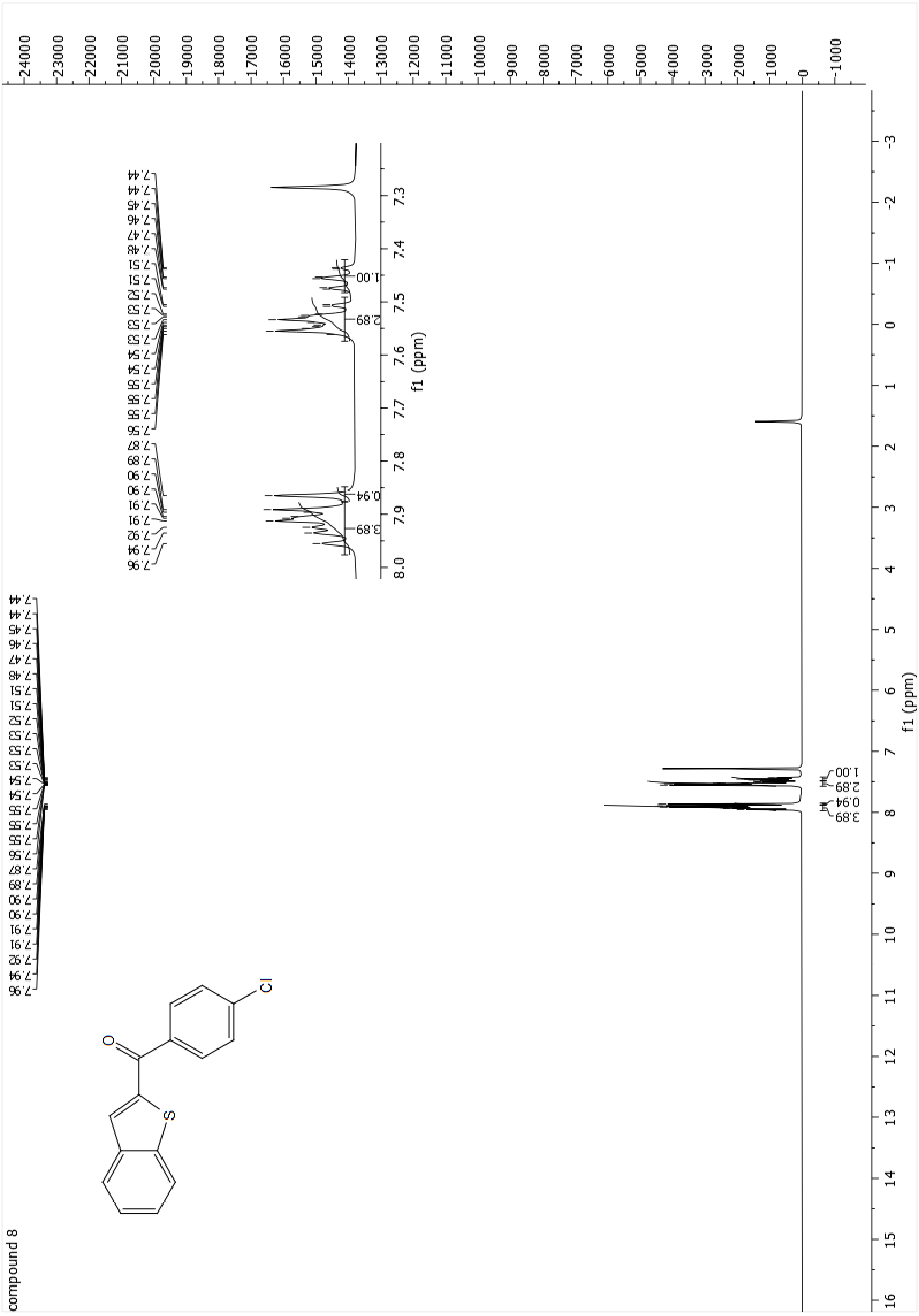

^{13}C NMR of compound 8

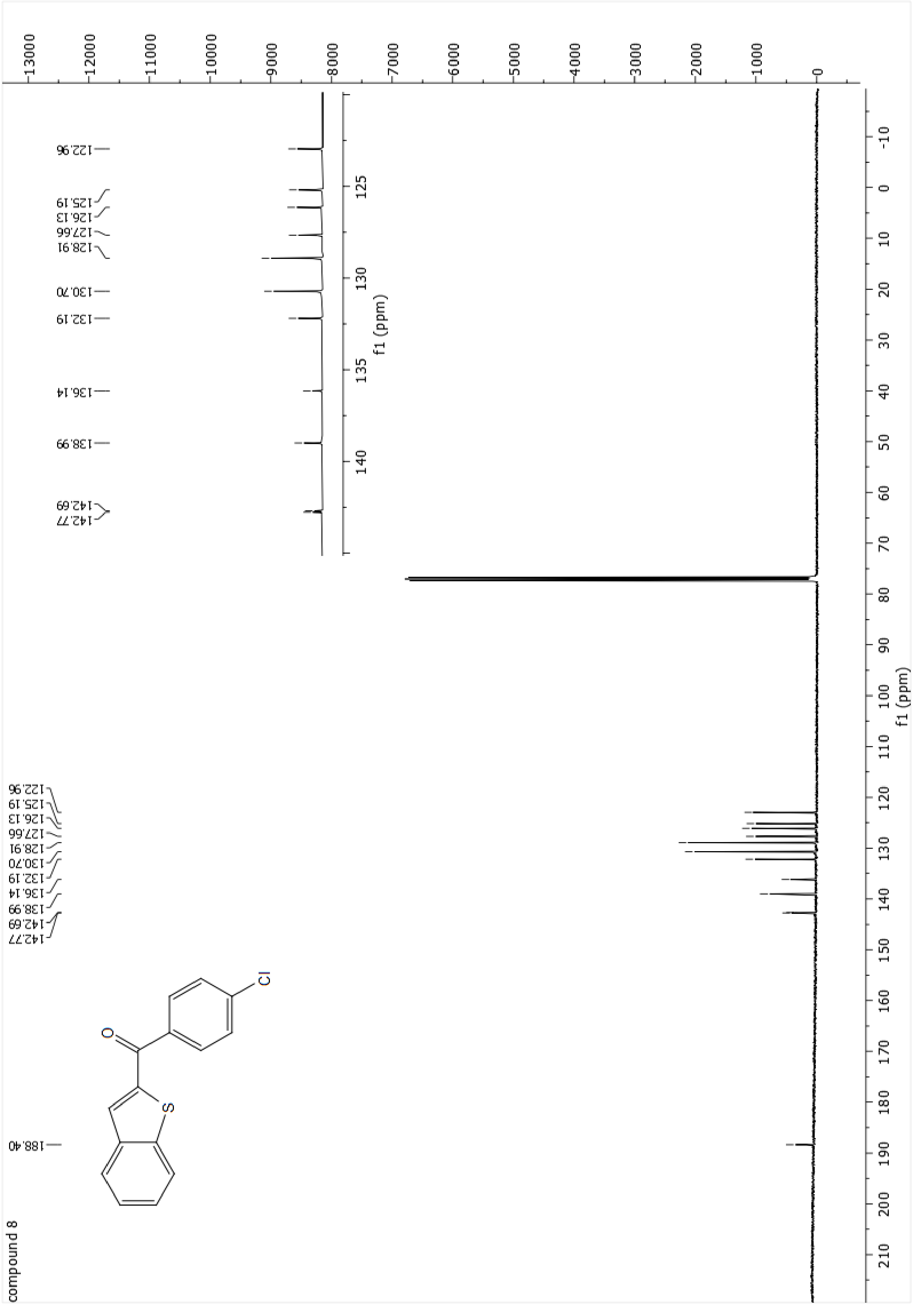

^{1}H NMR of compound 9

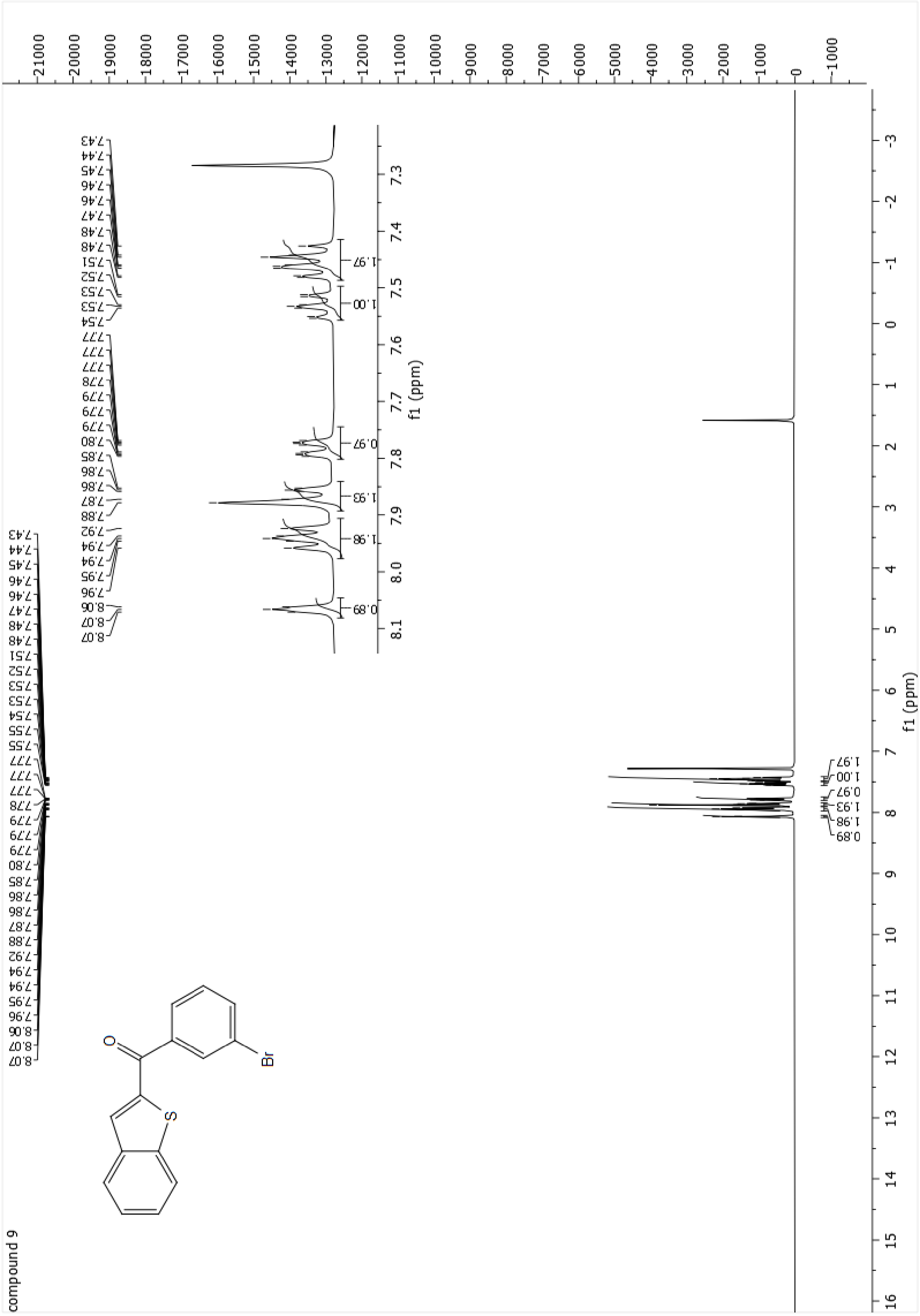

^{13}C NMR of compound 9

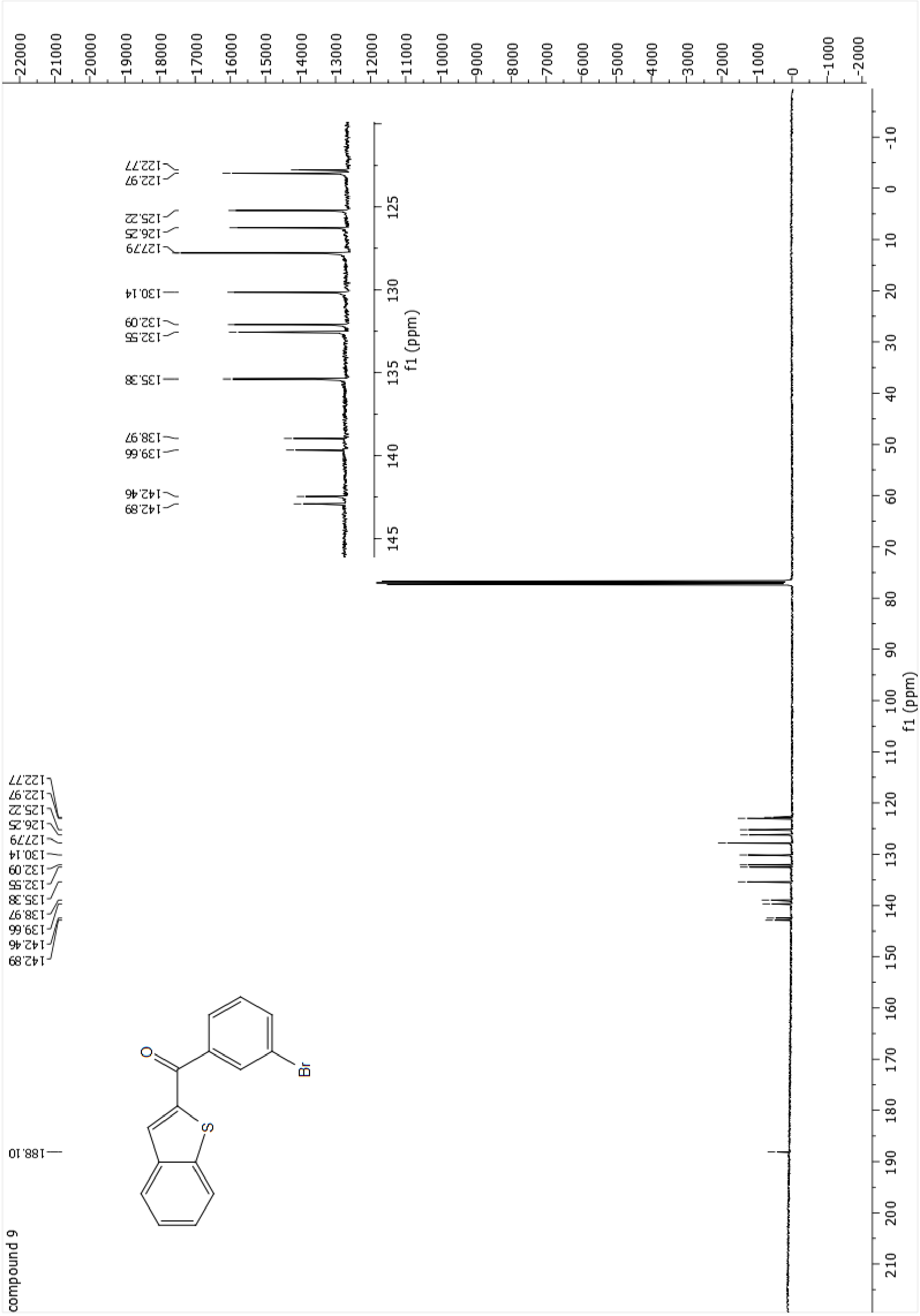

^{1}H NMR of compound 10

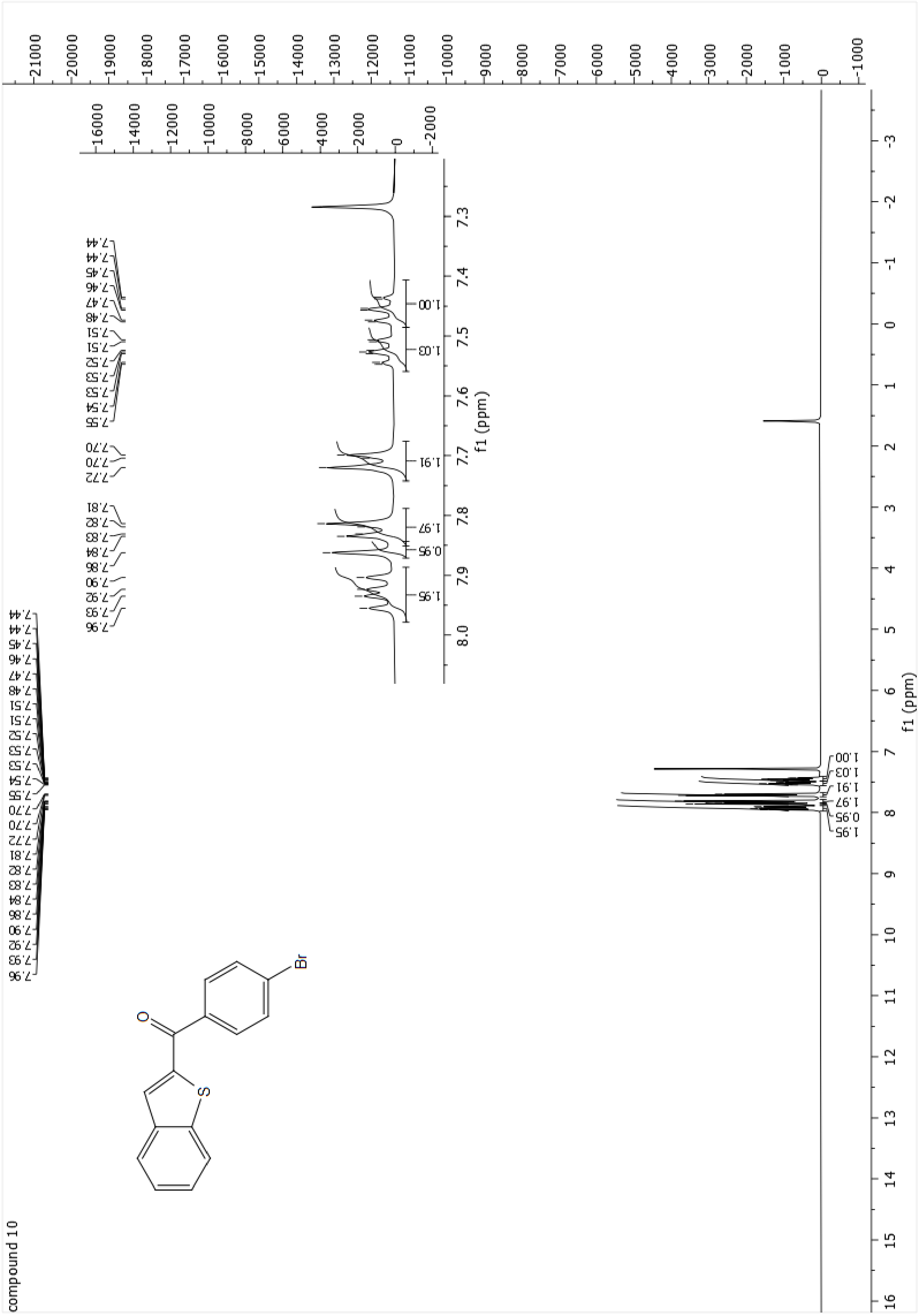

[13] C NMR of compound 10

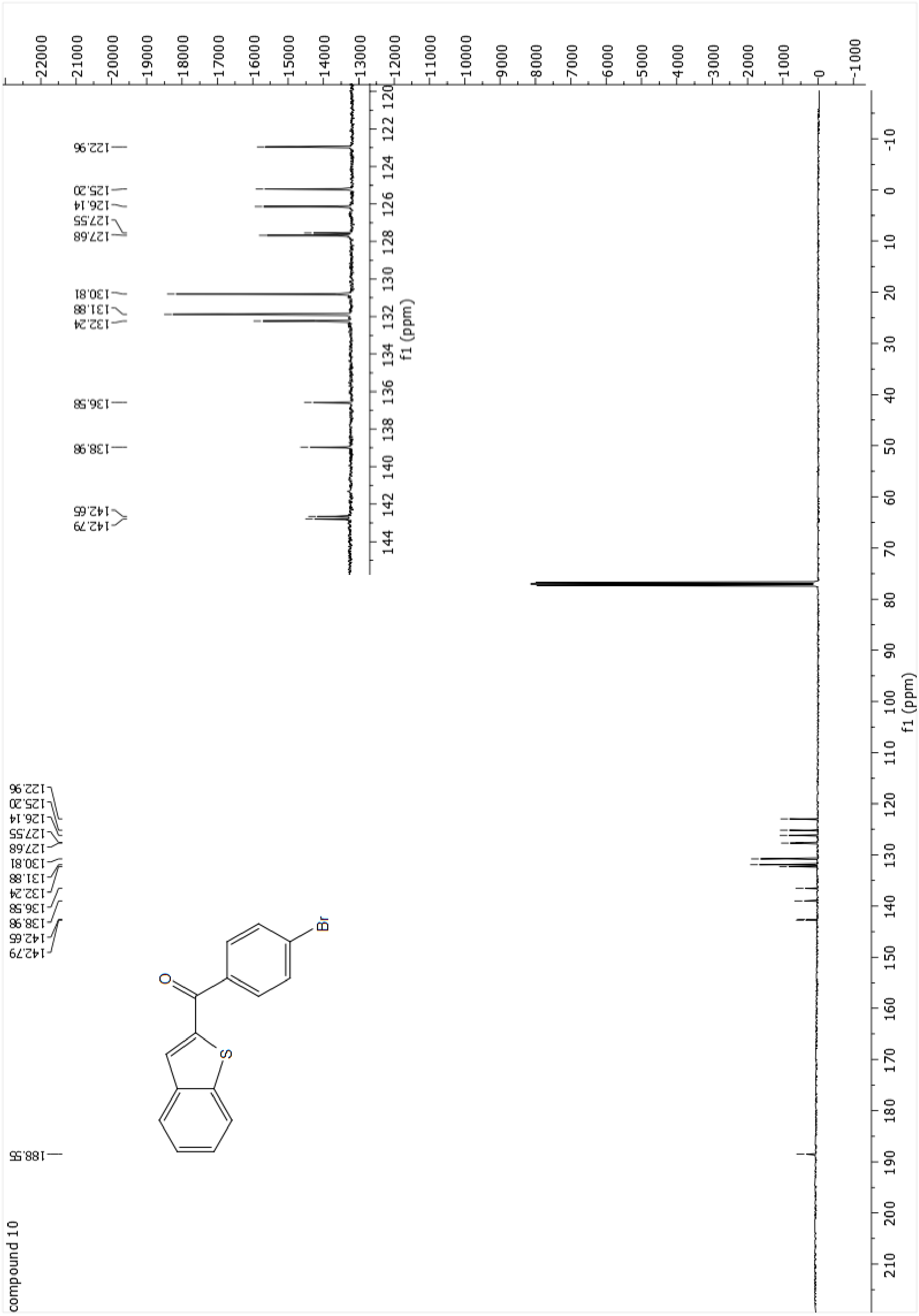

^{1}H NMR of compound 11

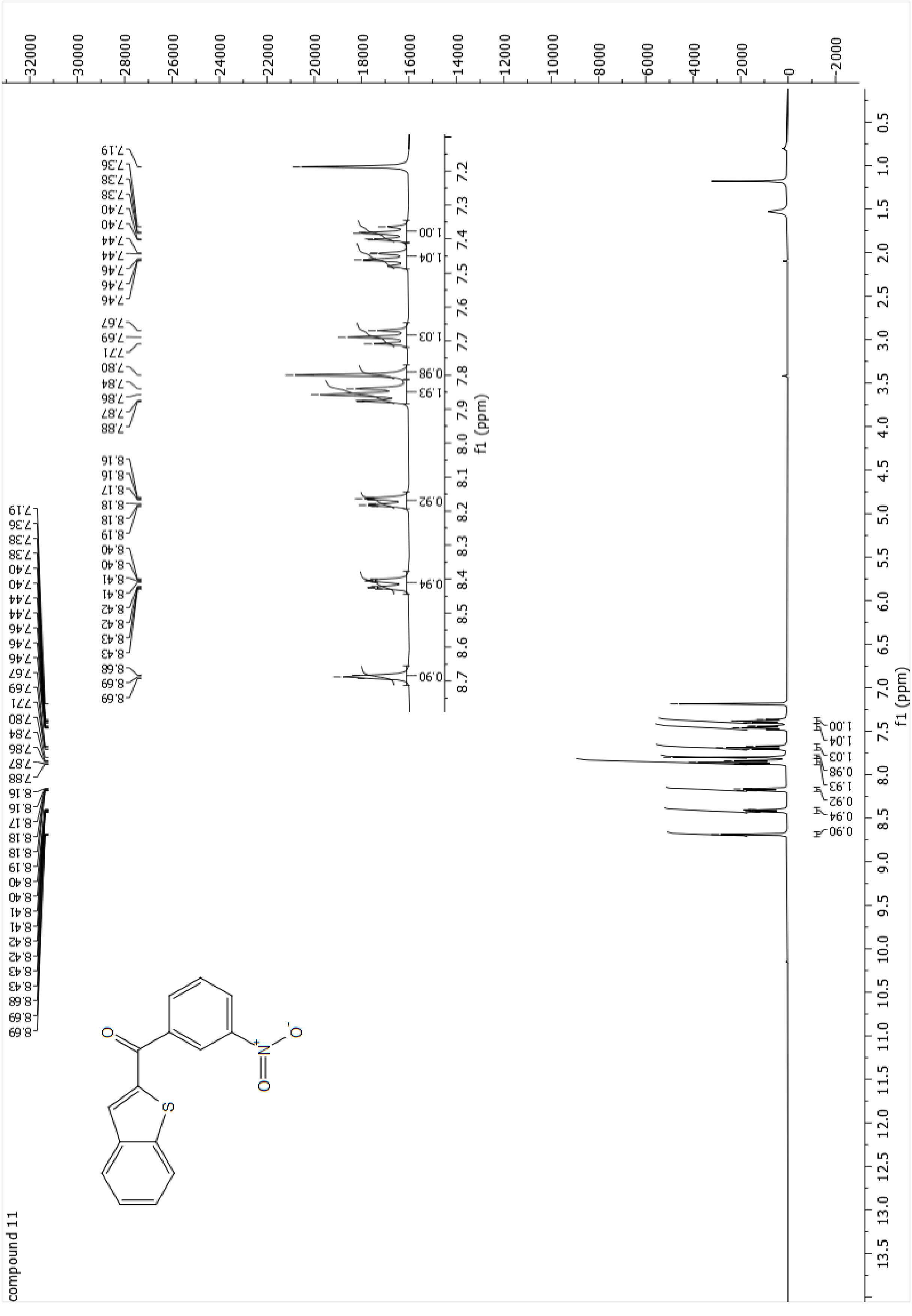

[13] C NMR of compound 11

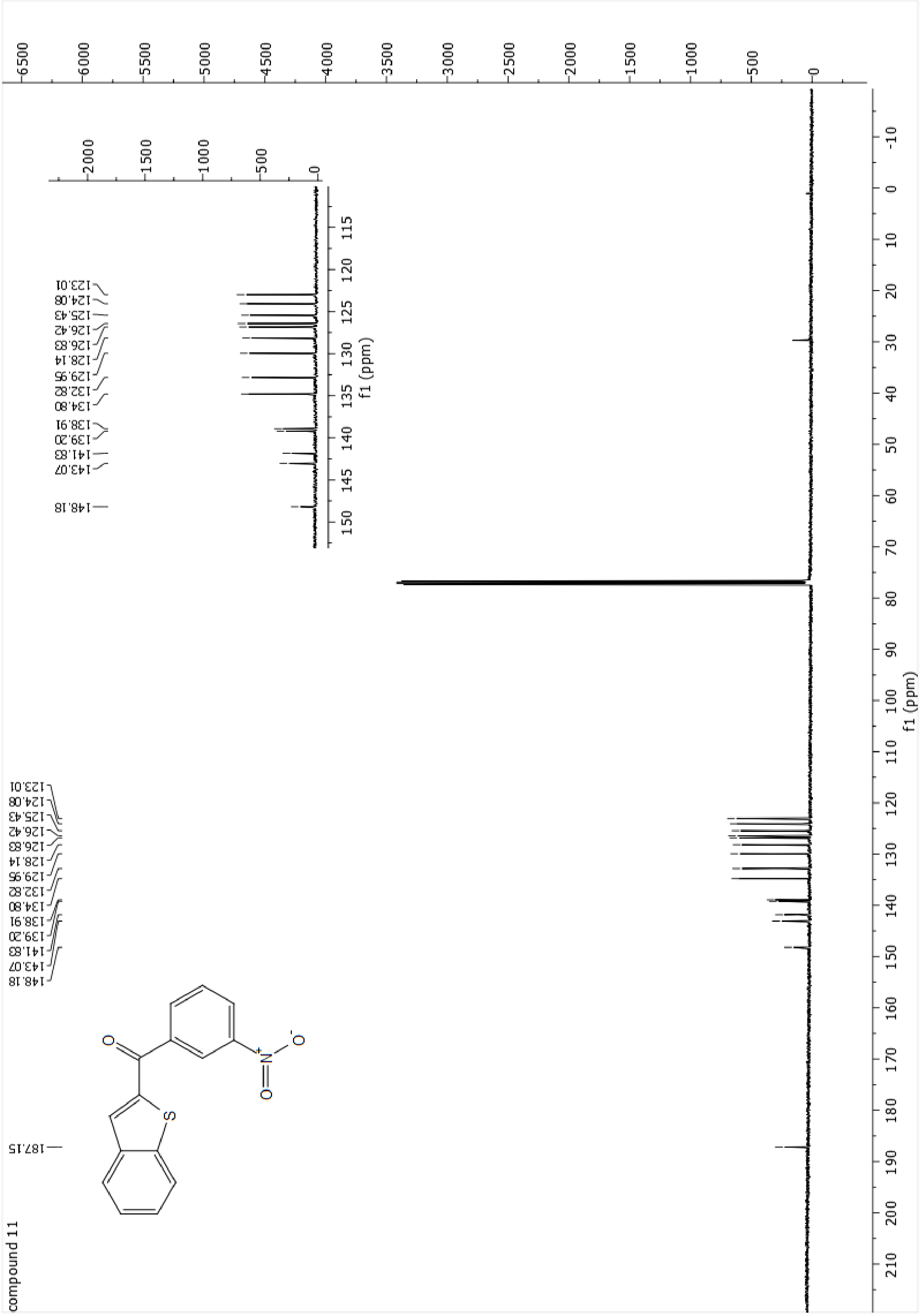

^{1}H NMR of compound 12

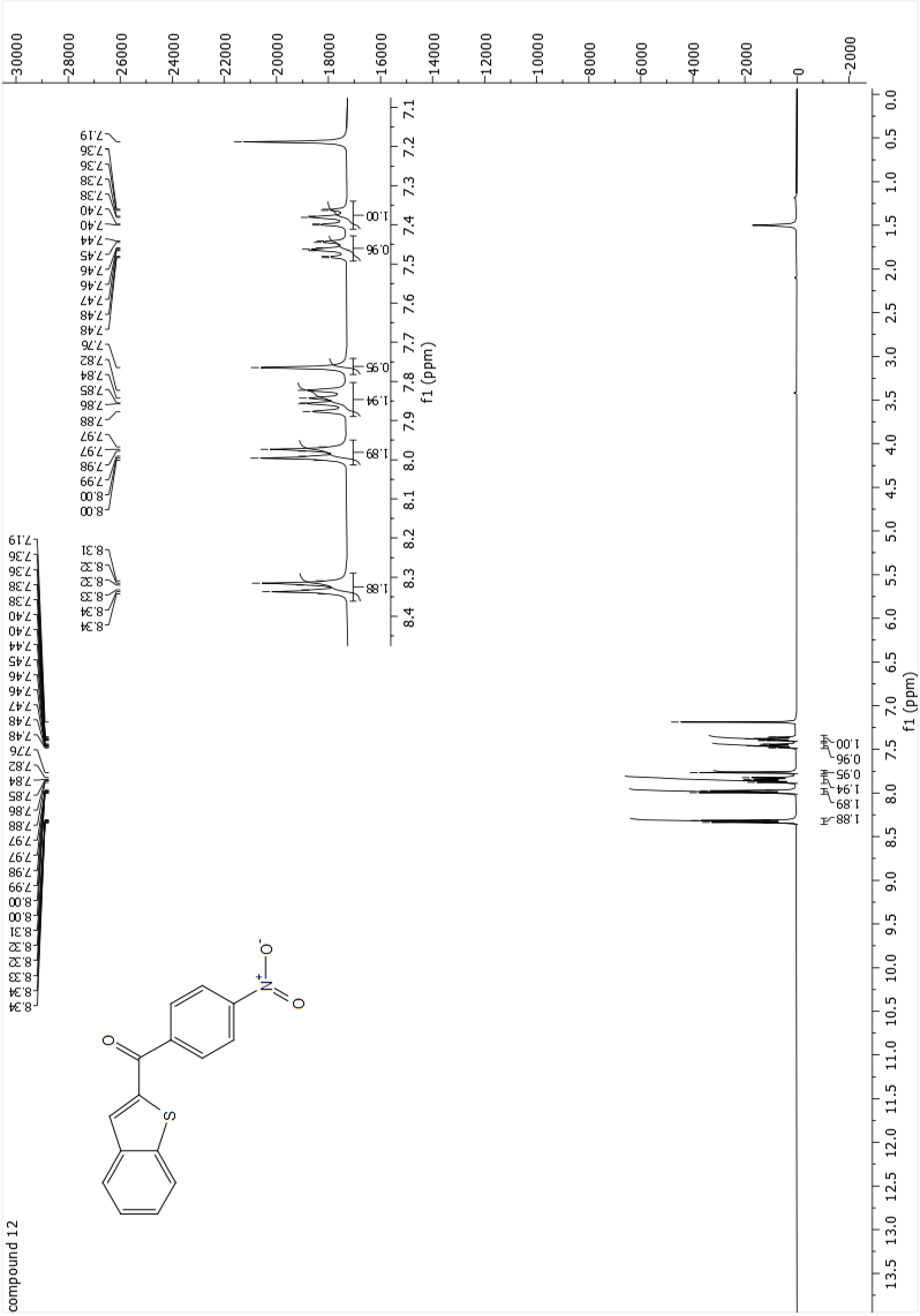

^{13}C NMR of compound 12

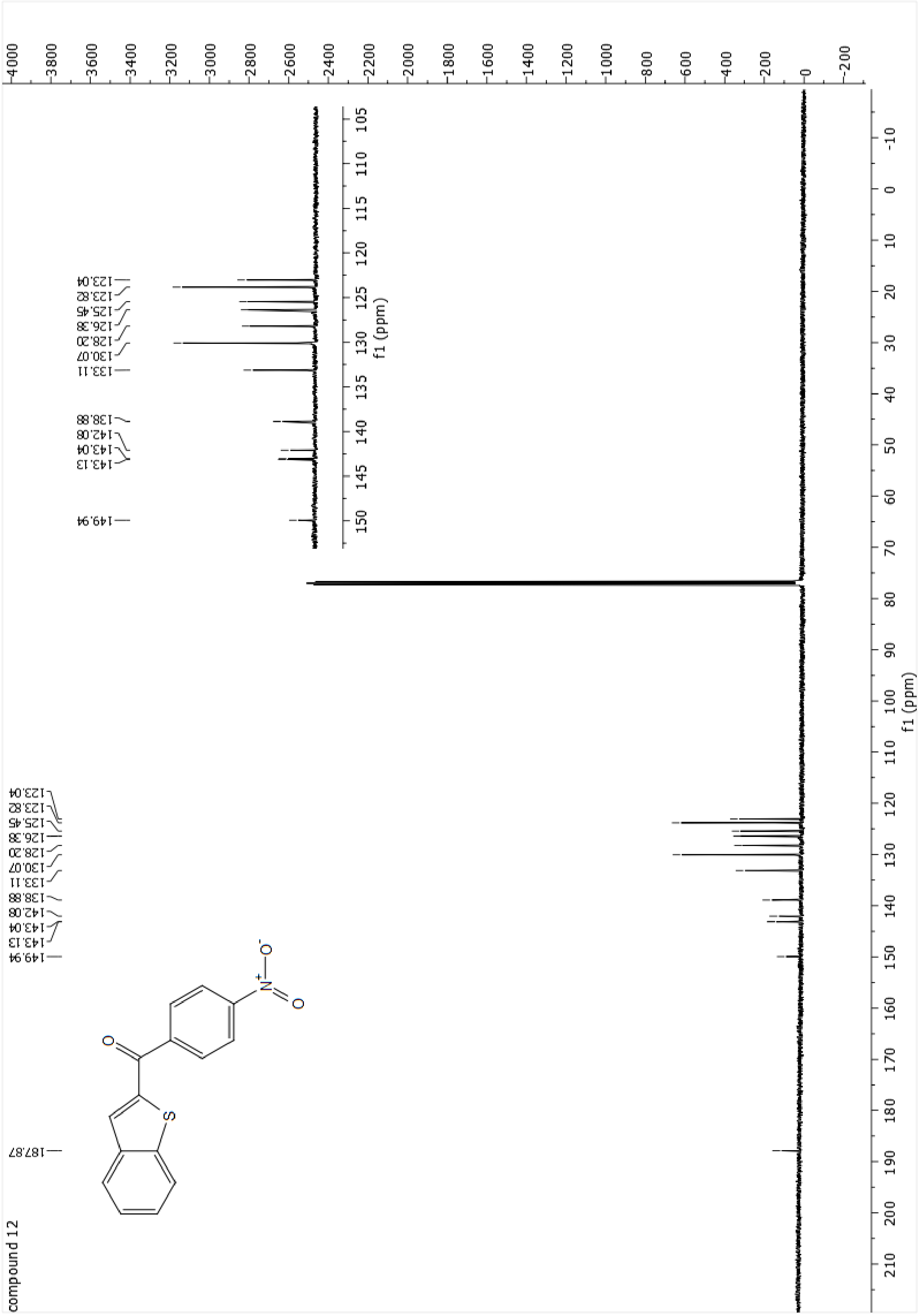

^{1}H NMR of compound 13

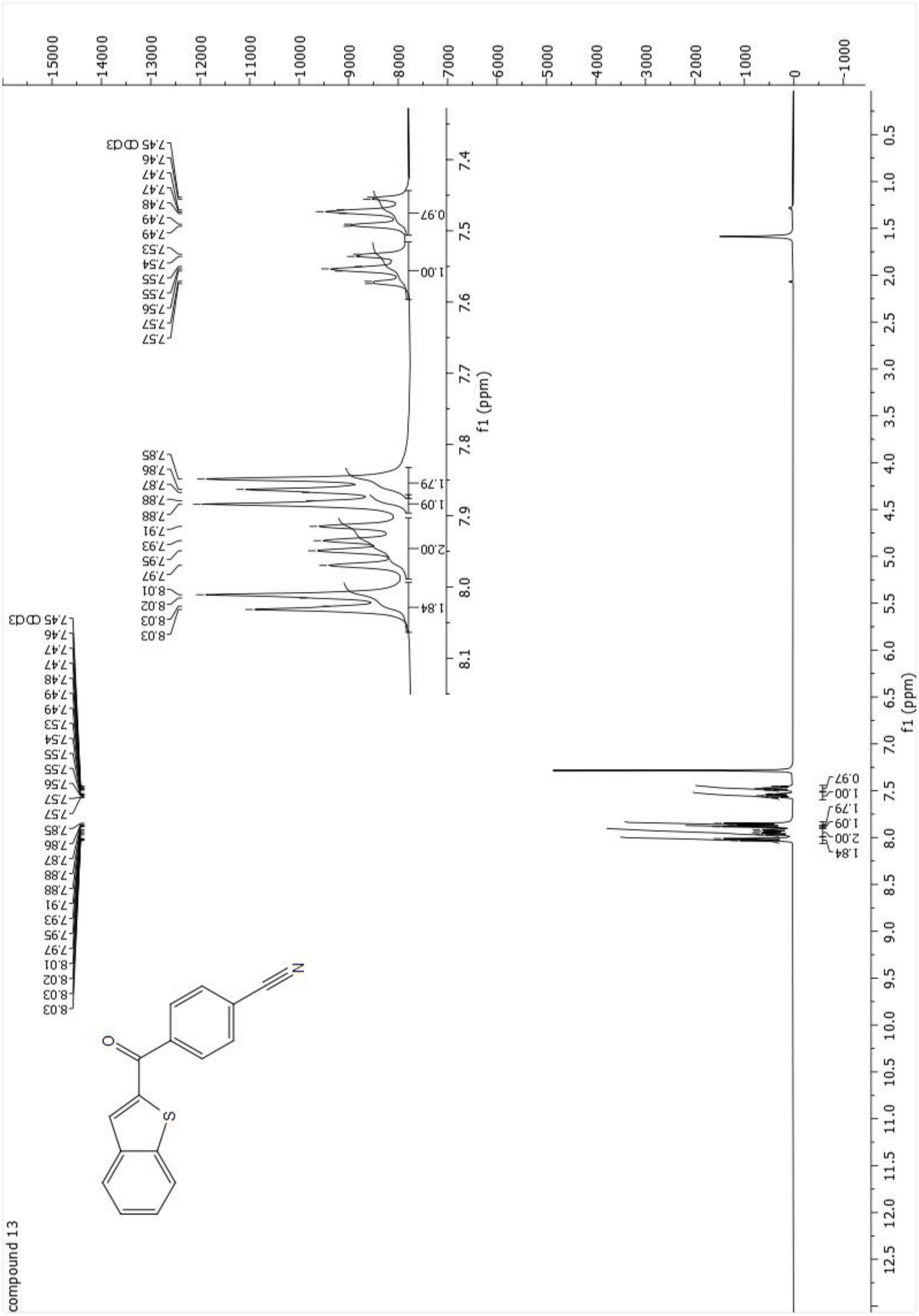

^{13}C NMR of compound 13

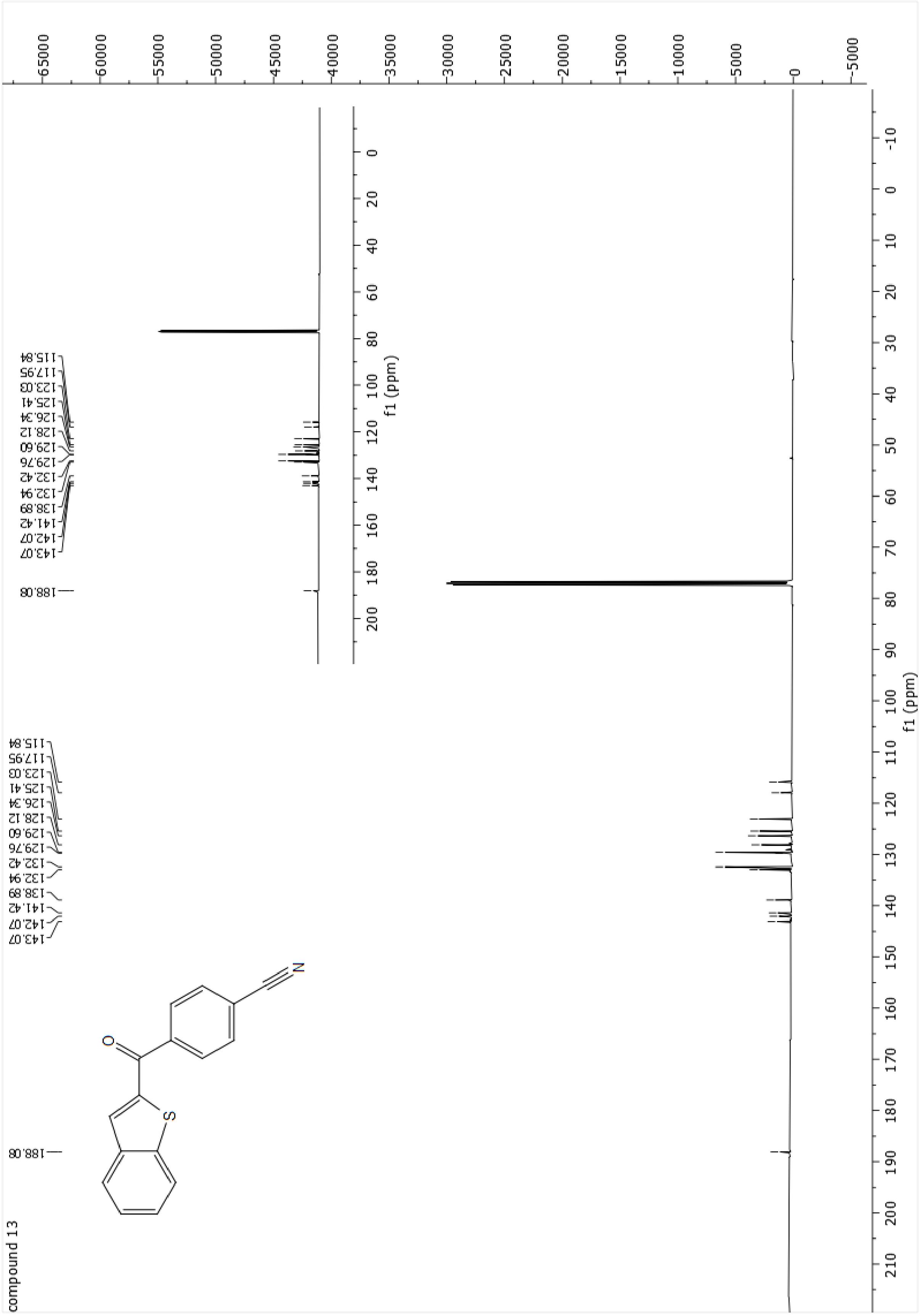

^{1}H NMR of compound 14

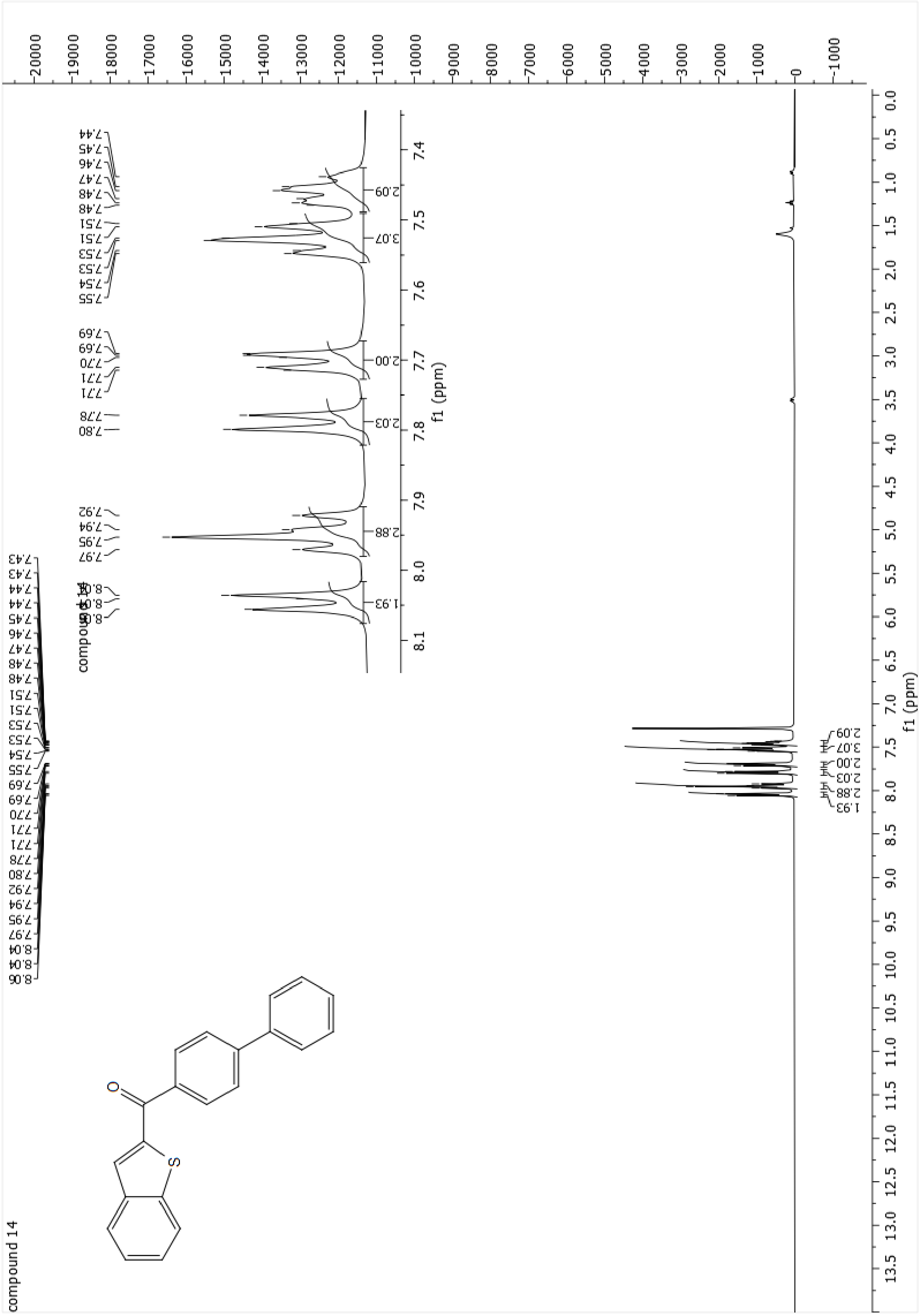

^{13}C NMR of compound 14

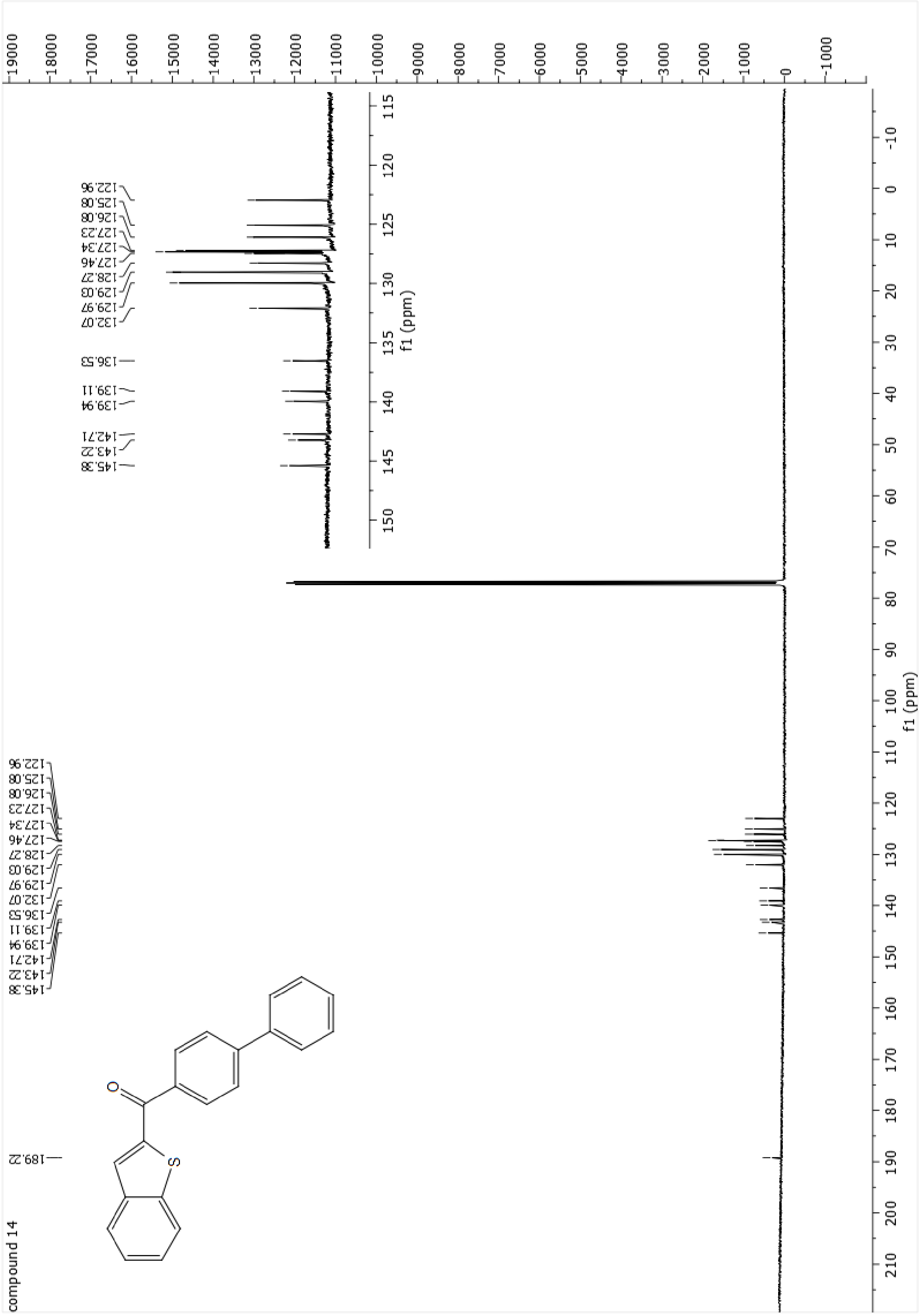

^{1}H NMR of compound 15

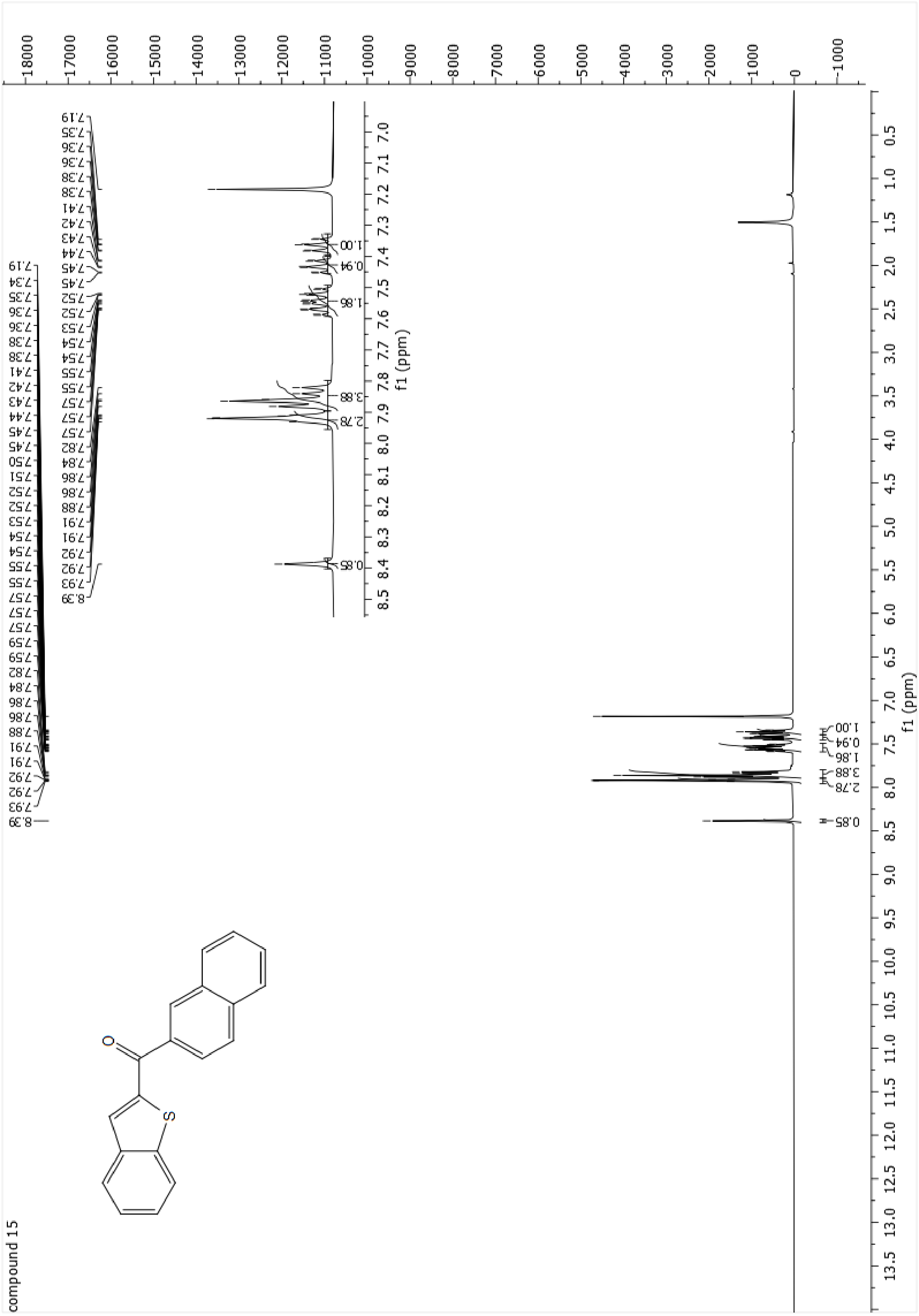

^{13}C NMR of compound 15

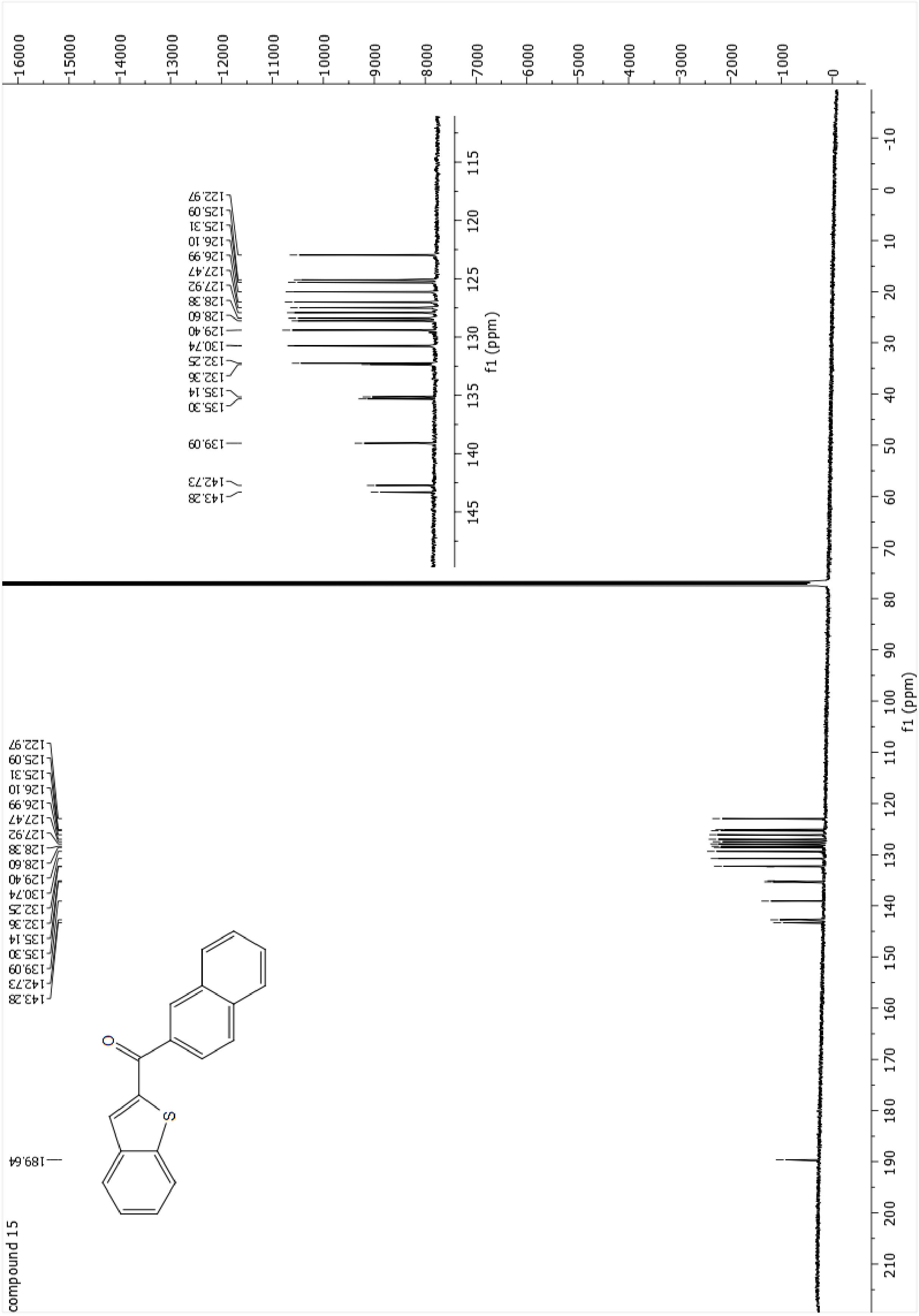

Mass spectrometric analysis

Compounds from **1** to **6**, **9**, **10**, **14**, and **15** were submitted to a direct flow injection analysis (FIA) by exploiting the autosampler and the pump modules of a Vanquish Core HPLC system (Thermo Fisher Scientific). In this configuration, the Orbitrap Exploris 120 was connected to the HPLC module and equipped with a high-flow H-ESI II source. Compounds were dissolved in acetonitrile at a concentration of 10^{-4} M and aliquots of 10 µL were injected into a continuous flow of acetonitrile containing 0.1% formic acid (v/v) at a flow rate of 0.200 mL/min. Typical ESI source parameters were as follows: spray voltage 3000 V, sheath gas (N_2) 35 a.u., auxiliary gas (N_2) 7 a.u., ion transfer tube temperature 320 °C, and vaporizer temperature 275 °C.

A second subset of samples, namely compounds **7**, **8**, and from **11** to **13**, was analyzed using an atmospheric-pressure matrix-assisted laser desorption/ionization (AP-MALDI) ultra-high-resolution ion source (AP-MALDI (ng) UHR, MassTech Inc., Columbia, MD, USA) coupled to the Orbitrap Exploris 120. Approximately 1 mg of each synthesized compound was suspended in 100 µL of acetonitrile/water (1:1, v/v), and 1 µL of the resulting suspension was deposited onto the wells of a MALDI target plate. The analytes were then overlaid with an equal volume of a 5 mg/mL solution of α-cyano-4-hydroxycinnamic acid (CHCA) dissolved in acetonitrile/ultrapure water/trifluoroacetic acid (50/50/0.1, v/v/v; LaserBio Labs, Valbonne, France), used as the MALDI matrix. After solvent evaporation, samples were irradiated with a 355 nm UV laser operating in spiral motion mode at a repetition rate of 1000 Hz and 1.5% laser power. The positive ionization voltage and ion transfer tube temperature were respectively set to 3000 V and 350 °C.

Mass Spectrum of Compound 1:

$[C_{15}H_{10}O^{32}S+H]^+$ - Theoretical mass: 239.05251 [Delta (ppm): 0.30].

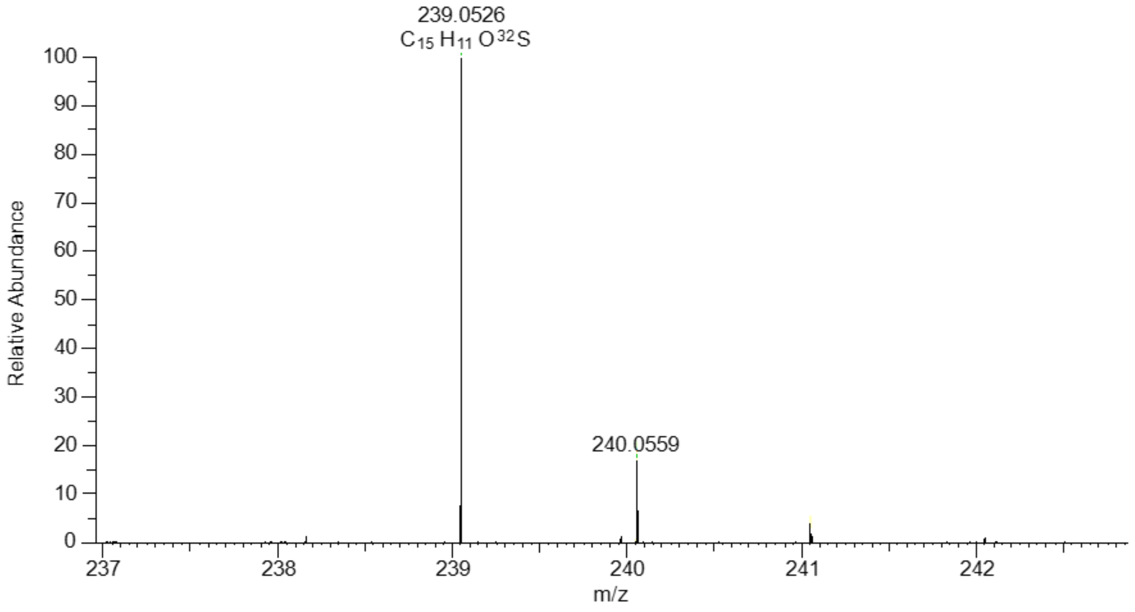

Mass Spectrum of Compound 2:

$[C_{16}H_{12}O^{32}S+H]^+$ - Theoretical mass: 253.06816 [Delta (ppm): 0.04].

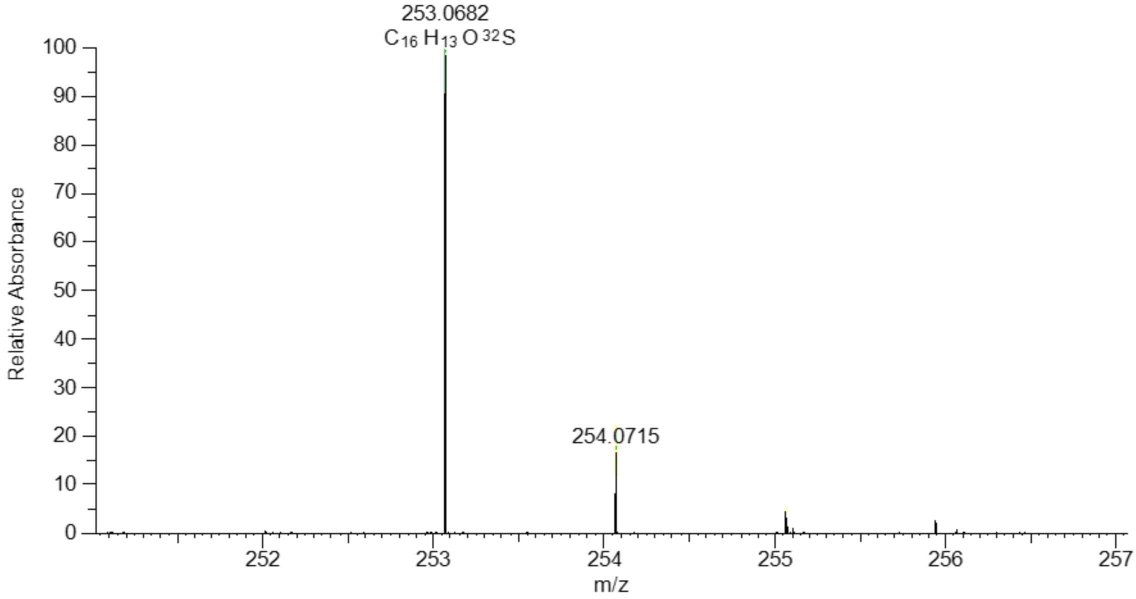

Mass Spectrum of Compound 3:

$[C_{16}H_{12}O_2{}^{32}S+H]^+$ - Theoretical mass: 269.06308 [Delta (ppm): 0.34].

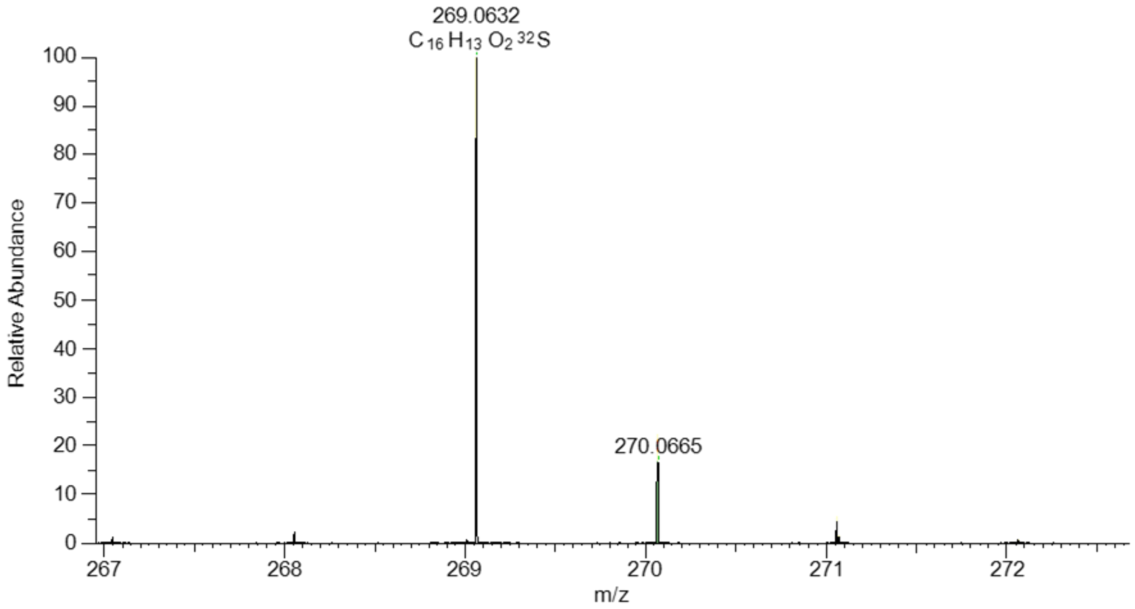

Mass Spectrum of Compound 4:

$[C_{16}H_{12}O_2{}^{32}S+H]^+$ - Theoretical mass: 269.06308 [Delta (ppm): 0.06].

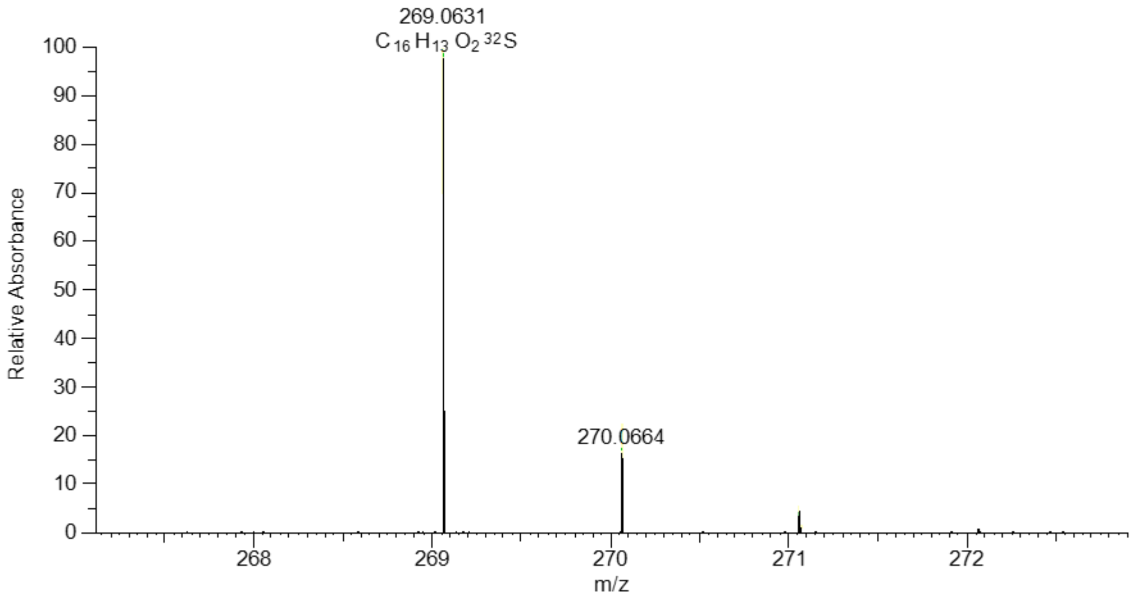

Mass Spectrum of Compound 5:

[$C_{15}H_9FO^{32}S+H]^+$ - Theoretical mass: 257.04309 [Delta (ppm): 0.24].

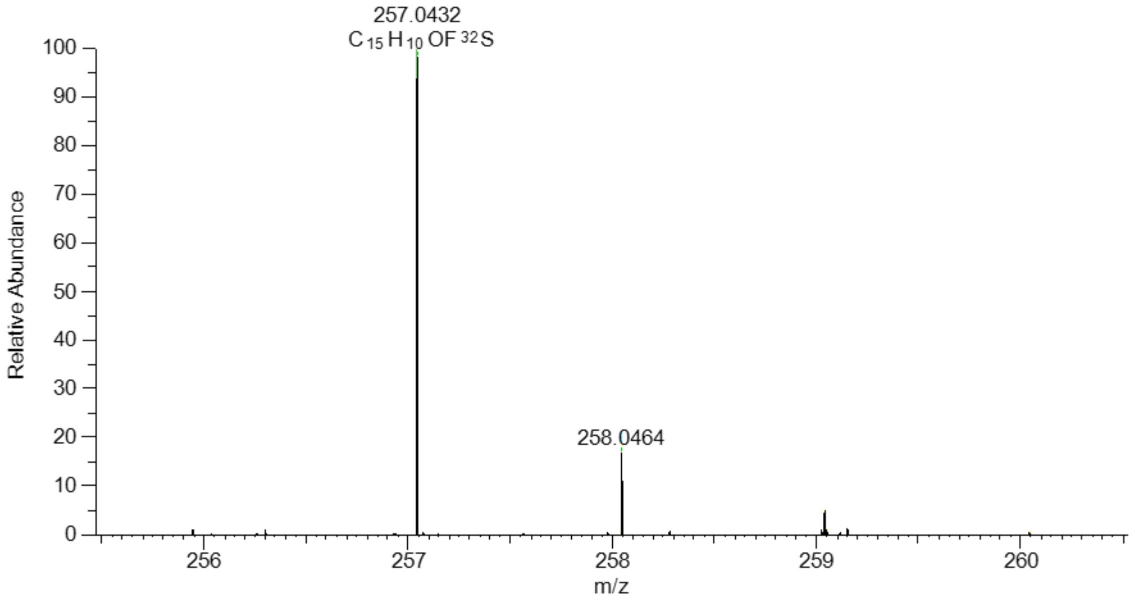

Mass Spectrum of Compound 6:

$[C_{15}H_{9}FO^{32}S+H]^{+}$ - Theoretical mass: 257.04309 [Delta (ppm): 0.12].

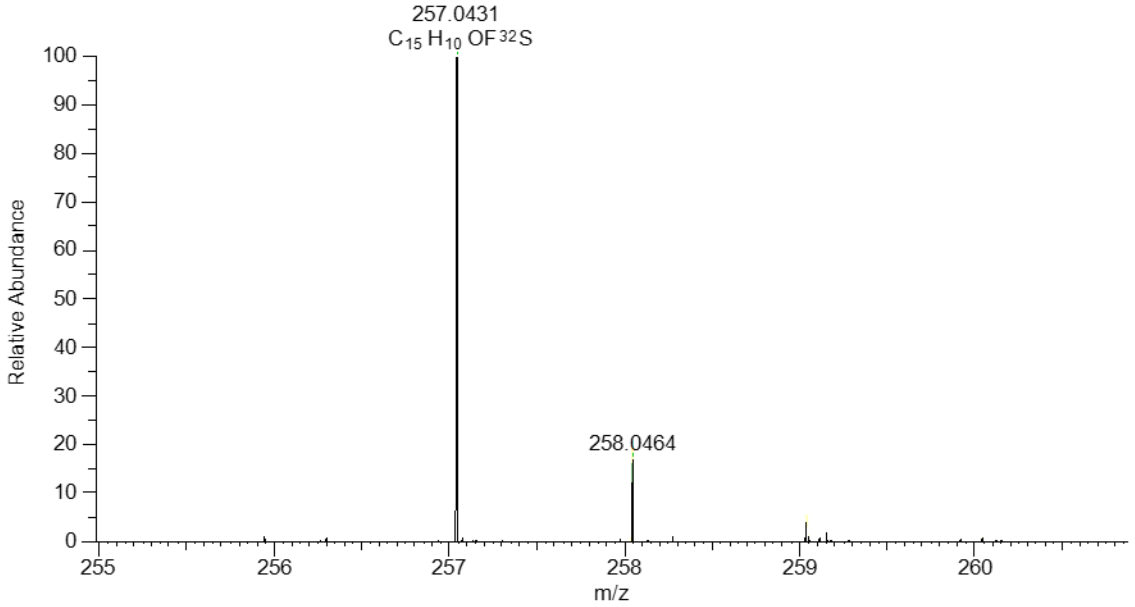

Mass Spectrum of Compound 7:

$[C_{15}H_{9}{}^{35}ClO^{32}S+H]^{+}$ - Theoretical mass: 273.01354 [Delta (ppm): -0.07]. The asterisks refer to the radical cations: $[C_{15}H_{9}{}^{35}ClO^{32}S]^{\cdot+}$ (Theoretical mass: 272.00572) and $[C_{15}H_{9}{}^{37}ClO^{32}S]^{\cdot+}$ (Theoretical mass: 274.00261).

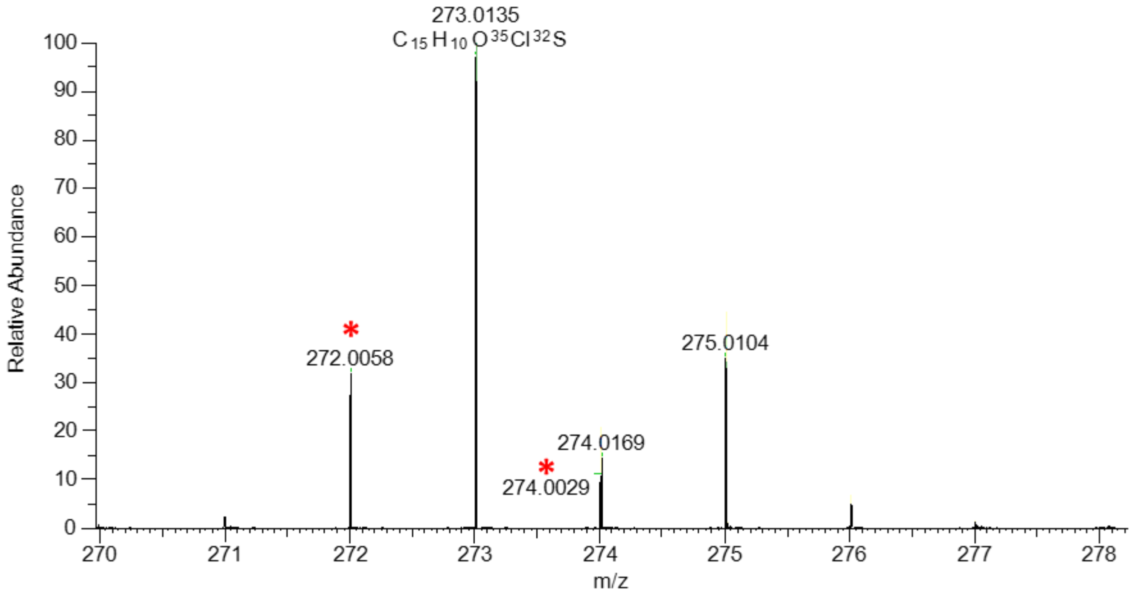

Mass Spectrum of Compound 8:

$[C_{15}H_{9}{}^{35}ClO^{32}S+H]^{+}$ - Theoretical mass: 273.01354 [Delta (ppm): 0.26]. The asterisks refer to the radical cations: $[C_{15}H_{9}{}^{35}ClO^{32}S]^{\cdot+}$ (Theoretical mass: 272.00572) and $[C_{15}H_{9}{}^{37}ClO^{32}S]^{\cdot+}$ (Theoretical mass: 274.00261).

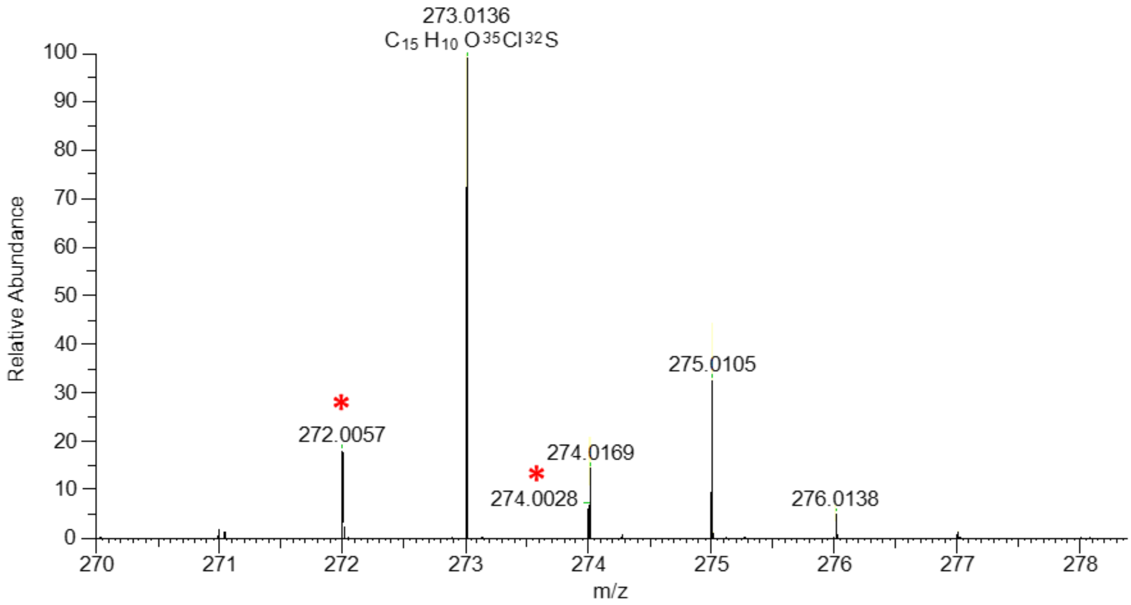

Mass Spectrum of Compound 9:

$[C_{15}H_9{}^{79}BrO^{32}S+H]^+$ - Theoretical mass: 316.96303 [Delta (ppm): 0.15].

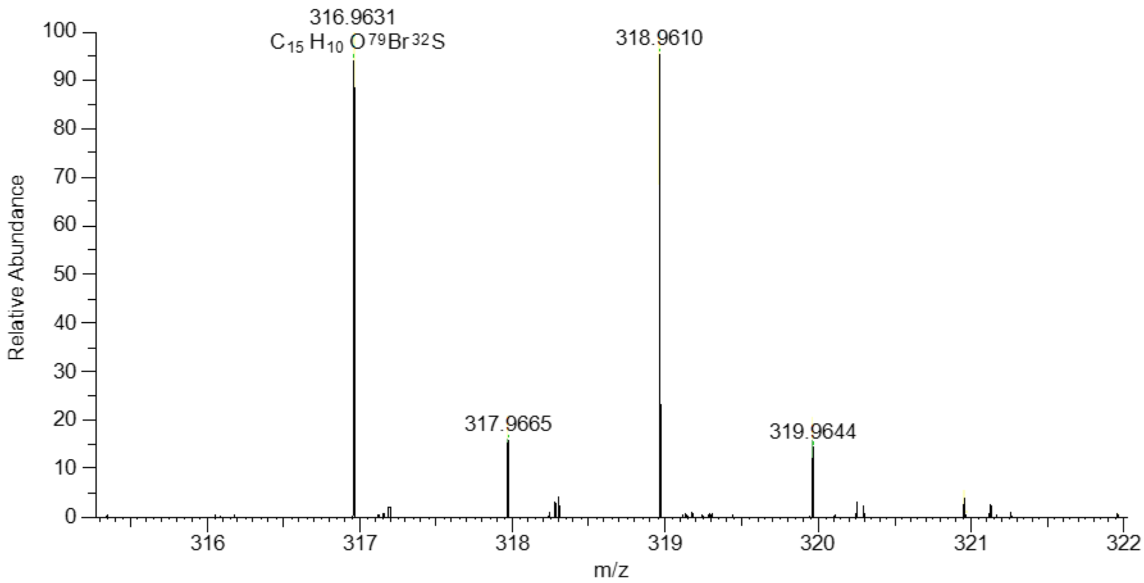

Mass Spectrum of Compound 10:

$[C_{15}H_{9}{}^{79}BrO^{32}S+H]^{+}$ - Theoretical mass: 316.96303 [Delta (ppm): 0.27].

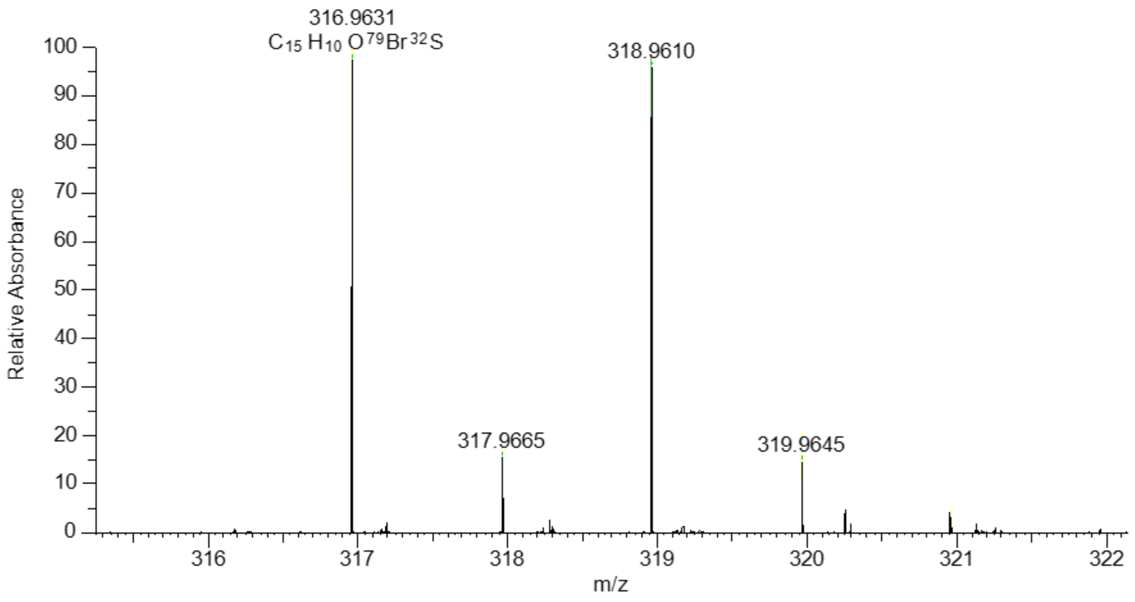

Mass Spectrum of Compound 11:

$[C_{15}H_9NO_3{}^{32}S+H]^+$ - Theoretical mass: 284.03759 [Delta (ppm): 0.13]. The asterisk refers to the radical cation: $[C_{15}H_9NO_3{}^{32}S]^{\cdot+}$ (Theoretical mass: 283.02977).

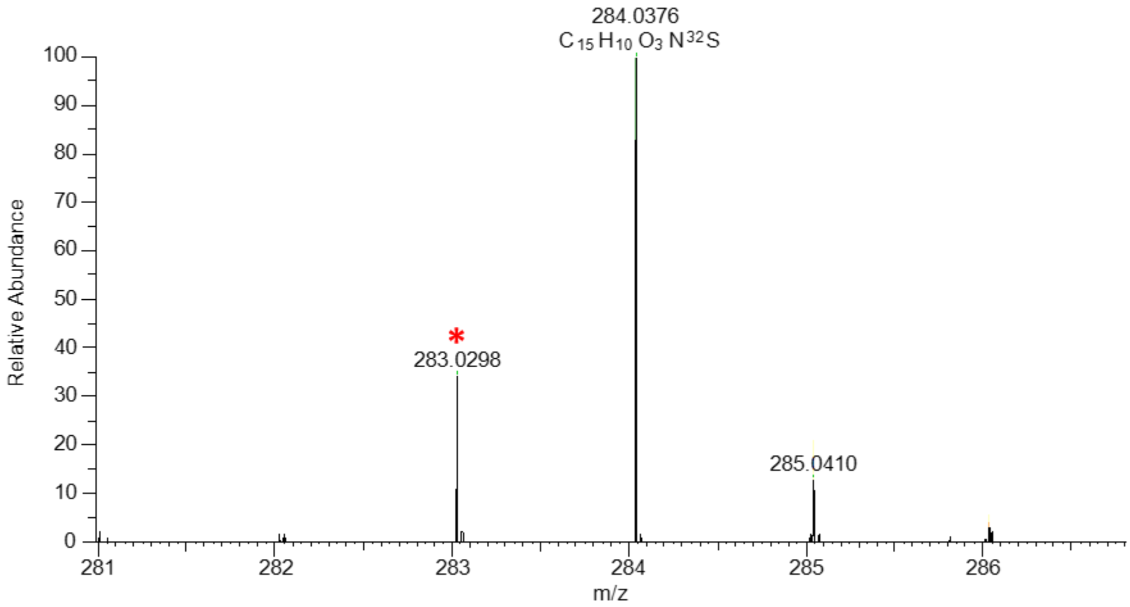

Mass Spectrum of Compound 12:

$[C_{15}H_9NO_3{}^{32}S]^{\cdot+}$ - Theoretical mass: 283.02977 [Delta (ppm): -0.15]. The asterisk refers to the protonated ion: $[C_{15}H_9NO_3{}^{32}S+H]^+$ (Theoretical mass: 284.03759).

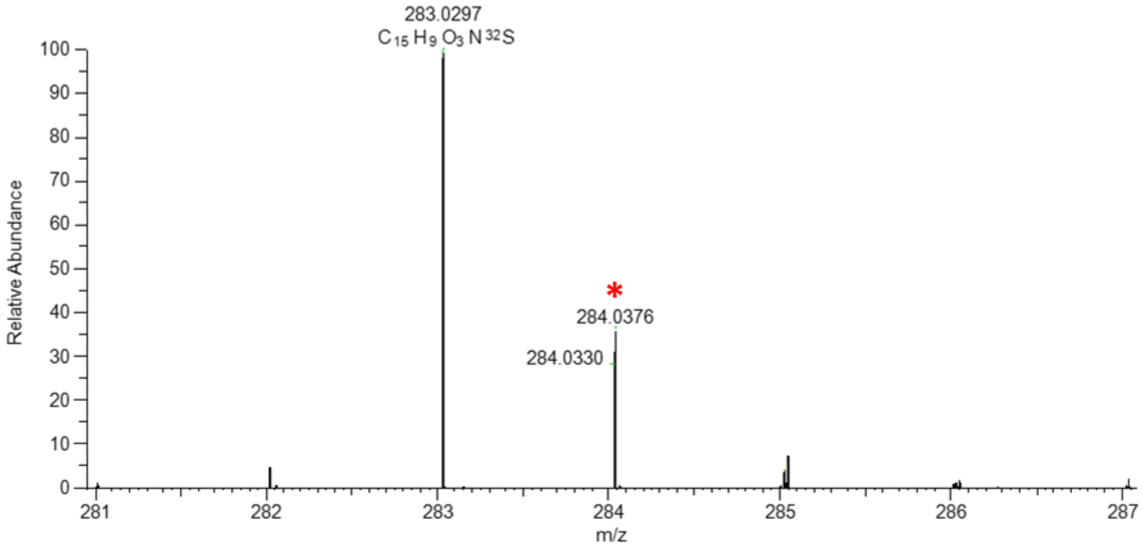

Mass Spectrum of Compound 13:

$[C_{16}H_9NO^{32}S]^{\cdot+}$ - Theoretical mass: 263.03994 [Delta (ppm): 0.28]. The asterisk refers to the protonated ion: $[C_{16}H_9NO^{32}S+H]^{+}$ (Theoretical mass: 264.04776).

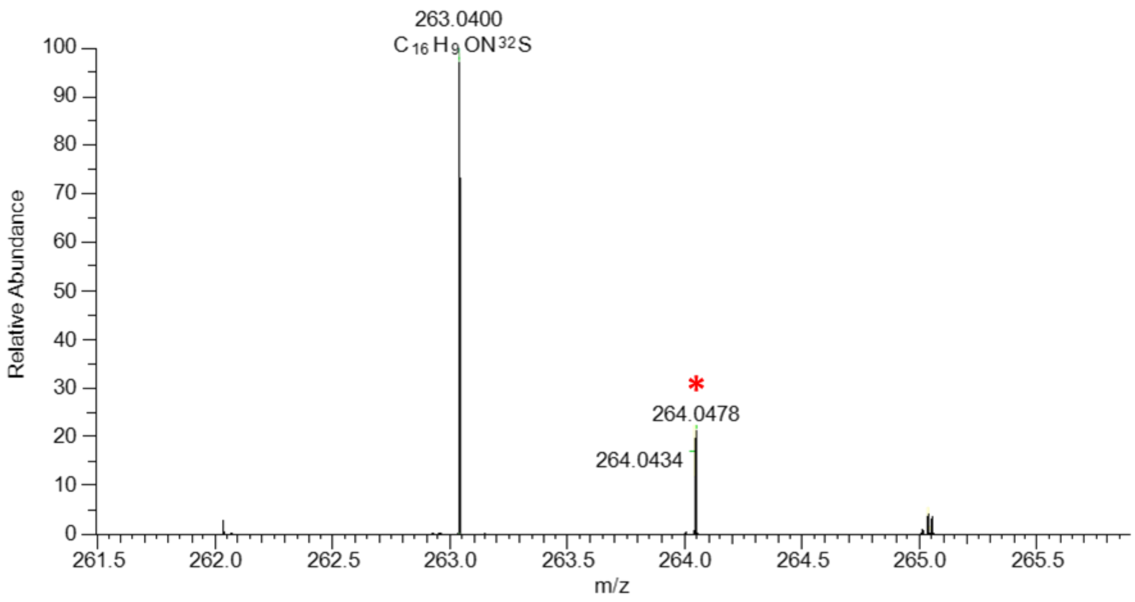

Mass Spectrum of Compound 14:

$[C_{21}H_{14}O^{32}S+H]^+$ - Theoretical mass: 315.08381 [Delta (ppm): 0.14].

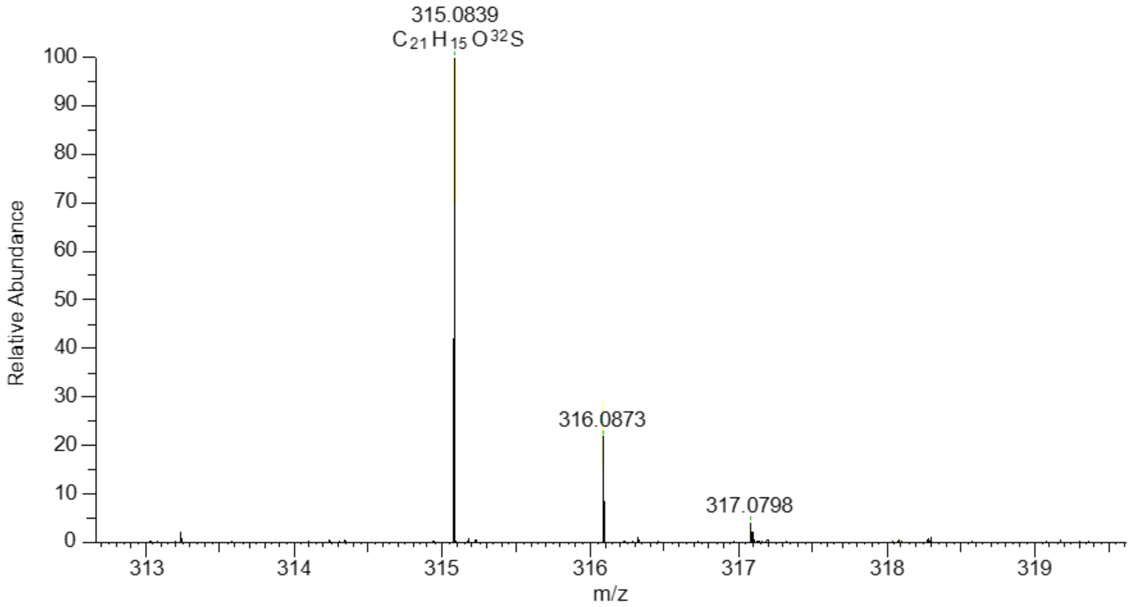

Mass Spectrum of Compound 15:

$[C_{19}H_{12}O^{32}S+H]^+$ - Theoretical mass: 289.06816 [Delta (ppm): 0.15].

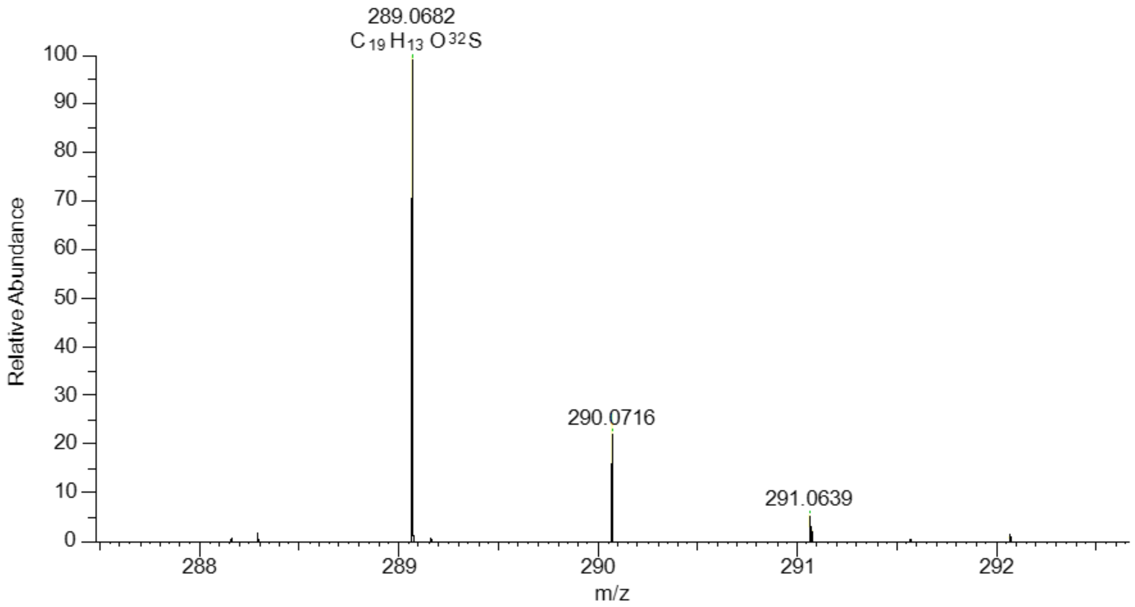

References

1. Guglielmi, P.; Secci, D.; Petzer, A.; Bagetta, D.; Chimenti, P.; Rotondi, G.; Ferrante, C.; Recinella, L.; Leone, S.; Alcaro, S.; et al. Benzo[b]Tiophen-3-Ol Derivatives as Effective Inhibitors of Human Monoamine Oxidase: Design, Synthesis, and Biological Activity. *J. Enzyme Inhib. Med. Chem.* **2019**, *34*, 1511–1525, doi:10.1080/14756366.2019.1653864.

2. Guglielmi, P.; Coluccia, M.; Marconi, G.D.; Ortuso, F.; Procopio, F.; Carradori, S.; Pizzicannella, J.; Arrighi, F.; Troiani, A.; Salvitti, C.; et al. Design, Synthesis, and Biological Activity of 2-Aroylbenzofuran-3-Ols and 2-Aroylbenzofuran Derivatives: A New Route towards HMAO-B Inhibition. *Eur. J. Med. Chem.* **2025**, *297*, doi:10.1016/j.ejmech.2025.117983.
